# Protective Efficacy of *Plasmodium vivax* Radiation-Attenuated Sporozoites in Colombian Volunteers: A Randomized Controlled Trial

**DOI:** 10.1371/journal.pntd.0005070

**Published:** 2016-10-19

**Authors:** Myriam Arévalo-Herrera, Juan M. Vásquez-Jiménez, Mary Lopez-Perez, Andrés F. Vallejo, Andrés B. Amado-Garavito, Nora Céspedes, Angélica Castellanos, Karen Molina, Johanna Trejos, José Oñate, Judith E. Epstein, Thomas L. Richie, Sócrates Herrera

**Affiliations:** 1 Malaria Vaccine and Drug Development Center (MVDC), Cali, Colombia; 2 Faculty of Health, Universidad del Valle, Cali, Colombia; 3 Asoclinic Inmunología LTDA, Cali, Colombia; 4 Centro Médico Imbanaco, Cali, Colombia; 5 Naval Medical Research Center, Malaria Department, Silver Spring, Maryland, United States of America; 6 Sanaria Inc, Rockville, Maryland, United States of America; 7 Caucaseco Scientific Research Center, Cali, Colombia; George Washington University School of Medicine and Health Sciences, UNITED STATES

## Abstract

**Background:**

Immunizing human volunteers by mosquito bite with radiation-attenuated *Plasmodium falciparum* sporozoites (RAS) results in high-level protection against infection. Only two volunteers have been similarly immunized with *P*. *vivax (Pv)* RAS, and both were protected. A phase 2 controlled clinical trial was conducted to assess the safety and protective efficacy of *Pv*RAS immunization.

**Methodology/Principal Findings:**

A randomized, single-blinded trial was conducted. Duffy positive (Fy+; *Pv* susceptible) individuals were enrolled: 14 received bites from irradiated (150 ± 10 cGy) *Pv*-infected *Anopheles* mosquitoes (RAS) and 7 from non-irradiated non-infected mosquitoes (Ctl). An additional group of seven Fy- (*Pv* refractory) volunteers was immunized with bites from non-irradiated *Pv*-infected mosquitoes. A total of seven immunizations were carried out at mean intervals of nine weeks. Eight weeks after last immunization, a controlled human malaria infection (CHMI) with non-irradiated *Pv*-infected mosquitoes was performed. Nineteen volunteers completed seven immunizations (12 RAS, 2 Ctl, and 5 Fy-) and received a CHMI. Five of 12 (42%) RAS volunteers were protected (receiving a median of 434 infective bites) compared with 0/2 Ctl. None of the Fy- volunteers developed infection by the seventh immunization or after CHMI. All non-protected volunteers developed symptoms 8–13 days after CHMI with a mean pre-patent period of 12.8 days. No serious adverse events related to the immunizations were observed. Specific IgG1 anti-*Pv*CS response was associated with protection.

**Conclusion:**

Immunization with *Pv*RAS was safe, immunogenic, and induced sterile immunity in 42% of the Fy+ volunteers. Moreover, Fy- volunteers were refractory to *Pv* malaria.

**Trial registration:**

Identifier: NCT01082341.

## Author Summary

Despite the advances in *Plasmodium falciparum* (*Pf*) vaccine development, progress in developing *P*. *vivax* (*Pv*) vaccines lags far behind. Immunization via mosquito bites with *Pf* radiation-attenuated sporozoites (RAS) has been the gold standard model for induction of sterile protection against malaria infection and has allowed the study of the complex mechanisms of immunity. The first trials using *Pf*RAS were performed in the late 1960’s, and thereafter greatly contributed to the development of vaccines against *Pf*. However, *Pv*RAS immunization in humans has only been carried out in two volunteers since 1974. To our knowledge, this is the first clinical trial using significant numbers of volunteers for *Pv*RAS immunization. Our findings confirm that immunization with *Pv*RAS is safe, immunogenic and induces sterile immunity in 42% of the volunteers. It demonstrates that it is possible to induce sterile protection with *Pv*RAS as seen with *Pf*RAS and confirms that immunity against the *Pv*CS protein (IgG1 levels) correlates with protection. Research findings and reagents generated in this study are expected to yield insights on key immune determinants of sterile protection against *Pv*, which may guide the development of a cost-effective vaccine against this parasite species.

## Introduction

Although there has been a decrease in malaria incidence globally during the past 15 years (~37%) [1], this infection remains a major public health problem with 214 million cases and 438,000 deaths estimated in 2015 [1]. *Plasmodium falciparum (Pf)* causes the greatest malaria burden particularly in Africa, and is the focus of most attention, including the search for a vaccine. Recently, a vaccine based on the *Pf* circumsporozoite (CS) protein (RTS,S) received a positive decision by the European Medicines Agency (EMA) for potential use in African children to reduce episodes of clinical malaria, based on the results of phase 3 studies, while the World Health Organization (WHO) recommended feasibility and pilot effectiveness implementations [2]. Protection afforded by RTS,S is limited to reduction of clinical disease in infants and young children; the vaccine is not intended for older children or adults, for use in Europe or the USA, or to block infection or prevent transmission. *Plasmodium vivax* (*Pv*) is the second most abundant malaria parasite, posing a serious threat in Asia, Oceania, and Latin America and also requires a specific and effective vaccine. Progress in developing *Pv* vaccines lags far behind that for *Pf*.

Acquisition of clinical immunity to malaria is a slow process and sterile immunity is never achieved under natural conditions, although it can be reproducibly induced by immunization via mosquito bite with radiation-attenuated sporozoites (RAS), the parasite stage transmitted by mosquitoes to humans [3–5]. This approach induces immune responses that block the sporozoite (SPZ) invasion of hepatocytes and subsequent schizogonic development in the liver, thereby preventing the pathogenic asexual blood stage infection that causes malaria disease. Such responses also prevent the development of gametocytes (sexual blood stages); thus, RAS immunization could serve as a vaccine to interrupt malaria transmission. Pre-erythrocytic stage vaccines such as RAS, therefore represent an ideal approach for vaccine development [6] as has been reported previously for *Pf* [7].

In the 1970s, sterile immunity against malaria was first demonstrated in humans vaccinated using RAS [3, 4, 8]. Since then, multiple studies have confirmed the high reproducibility of this vaccination model [9, 10]. Significant efforts are now being invested and good progress has been achieved in developing a parenterally injectable vaccine based on cryopreserved *Pf*RAS [7]. Several *Pf*RAS phase 1 and 2 trials have been conducted by Sanaria Inc. and collaborators, using a *Pf*SPZ vaccine, a GMP product consisting of aseptic, purified, radiation-attenuated, cryopreserved *Pf*SPZ. This vaccine has shown high-level efficacy in naïve adults [7]. Additionally, several parasite antigens found to be active in RAS immunization and possibly associated with protection have been the subject of intense research on the development of subunit vaccines (reviewed in [11]).

Despite the epidemiological importance of *Pv*, the *Pv*RAS model has not been reproduced since the early 1970s, when two volunteers were immunized by receiving >1000 mosquito infectious bites; both were protected from infectious *Pv* spz challenge [12]. This lag is partly explained by the lack of *Pv in vitro* culture methods, promoting the development of alternative, more complex infection methods that rely on obtaining fresh, gametocytemic blood from *Pv*-infected donors. *Anopheles* mosquito colonies have been established [13] and methods to routinely infect mosquitoes using blood from acutely ill *Pv* malaria patients have now been standardized [14], resulting in safe, reliable and reproducible infection of human volunteers through mosquito bites [15–17]. The purpose of the study described here was first to establish a solid proof-of-principle that humans could be protected by immunization via the bites of *Pv*RAS-infected mosquitoes and second, to obtain sera and cells to study the mechanisms of protective immunity and identify the antigenic targets of immune responses. A phase 2 trial was conducted in healthy adult Colombian volunteers without previous exposure to malaria.

## Methods

### Ethics statement

This trial was conducted according to ICH E-6 Guidelines for Good Clinical Practices [18]. Institutional Review Boards of the Malaria Vaccine and Drug Development Center (MVDC, CECIV), and Centro Médico Imbanaco (CEICMI), Cali, approved the protocol. Written informed consent (IC) was obtained from all volunteers, with a separate IC for HIV screening. The clinical trial was registered on clinicaltrials.gov, registry number NCT01082341. The protocol for this trial and supporting CONSORT checklist are available as supporting information (S1 Checklist and S1 Protocol).

### Study design and participants

**Fig 1:**
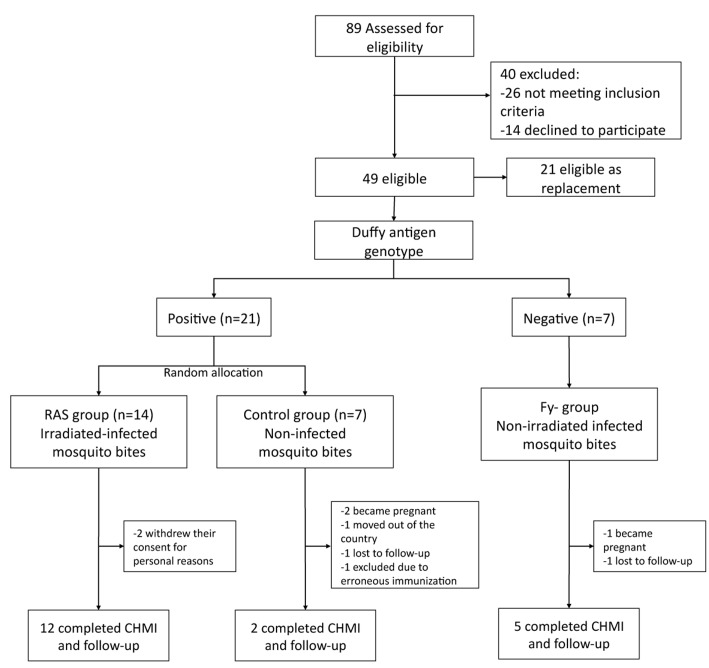
Trial flow diagram. Number of individuals in the screening, immunization, and CHMI steps.

A phase 2 controlled randomized, single-blinded clinical trial was conducted at the MVDC, Cali, Colombia. A total of 89 malaria-naïve volunteers (18–45 years old) were assessed for eligibility (Fig 1). Two approaches to immunization were used in this study. First, Duffy-positive (Fy+) individuals were assigned to RAS or mock-immunized control groups using a single-blinded design (volunteers but not investigators blinded) to assess the safety, tolerability, immunogenicity and protective efficacy of *Pv*RAS immunization. Second, taking advantage of the fact that Fy- erythrocytes are refractory to *Pv* invasion, a third group of Fy- volunteers was immunized with bites from infected non-irradiated mosquitoes to assess the impact of exposure to *Pv*SPZ developing fully in the liver (as opposed to arresting early in liver stage development, as in the case of RAS). Immunization was performed by direct exposure to bites of irradiated (Fy+ volunteers) or non-irradiated (Fy- volunteers) *Pv*-infected mosquitoes, and mock immunization by exposure to the bites of non-irradiated, non-infected mosquitoes. After the immunization schedule, volunteers were subjected to a *Pv* controlled human malaria infection (CHMI), carried out by exposing volunteers to the bites of non-irradiated, *Pv*-infected mosquitoes. Clinical outcome, parasitemia as measured by thick blood smear microscopy (TBS), and clinical laboratory and immunological parameters were assessed. Antimalarial treatment was provided to all volunteers becoming TBS-positive or completing the study to day 60 post-CHMI.

Volunteers were informed about the risks of participation and were provided sufficient opportunity to read the IC forms. Before signing the IC, volunteers had to pass an oral or written exam concerning the trial and its risks as described elsewhere [16]. In addition, all participants were informed about their right to voluntarily withdraw from the study at any time for any reason. Exclusion criteria included pregnancy, abnormal clinical hematology, and chemistry test results, glucose-6-phosphate dehydrogenase deficiency (G6PDd), and infectious diseases (syphilis, HIV, Chagas disease, HTLV 1–2, hepatitis B and hepatitis C; S1 Table, S2 Table, S1 text).

### Mosquito infection and irradiation

*Anopheles albimanus* mosquitoes reared at the MVDC insectary in Cali were infected with blood from *Pv-*infected patients (18–45 years old) recruited at outpatient clinics in malaria-endemic areas of Colombia. TBS was performed on all volunteers seeking care for malaria diagnosis as required by the National Malaria Control Program. Only volunteers who tested positive by this method were invited to participate in the study and were informed of the research aims, potential risks, and benefits. After signing the IC and before the antimalarial treatment, whole blood (35 mL) was collected by venipuncture. All samples were confirmed to be *Pv* malaria mono-infections by quantitative PCR (qPCR) and negative for other infectious agents (syphilis, HIV, Chagas disease, HTLV 1–2, hepatitis B and hepatitis C; S2 Table). Mosquitoes were membrane-fed with infected blood as described previously [19]. Batches with >50% mosquitoes harboring spz in their salivary glands were used for immunization and CHMI. For both procedures, individual screen-meshed boxes containing infected mosquitoes were used. Mosquitoes were allowed to feed on the volunteer for a 5–10 minute period as previously standardized [14]. After biting, all mosquitoes were dissected and microscopically examined to confirm the presence of blood meal and spz in the salivary glands. CHMI of all volunteers was carried out on the same day by exposing volunteers to bites of 2–4 mosquitoes infected with the same parasite isolate [15–17]. Infected bites were calculated as the number of fed mosquitoes times the percentage infected.

Sporozoite attenuation was performed by exposure of *Pv-*infected mosquitoes to 150 ± 10 cGy of gamma radiation using a Varian Clinac IX Series 927 linear accelerator at the radiotherapy unit of Hospital Universitario del Valle in Cali as previously described [20].

### Immunization, CHMI and blood sample collection

**Fig 2:**
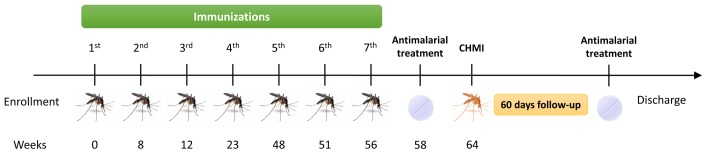
Study design. Immunization schedule for the three groups of volunteers (RAS, Ctl, and Fy-) who received seven immunizations and then were challenged with *P*. *vivax* field isolate infected mosquitoes.

The primary objective of the study was the immunization and CHMI of all volunteers using mosquitoes as described above. Fy+ volunteers were assigned to either RAS (n = 14) or Ctl (n = 7) groups, and Fy- volunteers to the Fy- group (n = 7). A total of seven immunizations were carried out using for each immunization a mean of 65 infectious mosquito bites. Two weeks after the last immunization, all volunteers were treated orally with curative doses of chloroquine (600 mg on day one and 450 mg on days two and three) and primaquine (30 mg daily for 14 days) to eliminate any subpatent malaria infections that may have developed during the immunization period, so that incident infections from CHMI could be accurately determined. Plasma levels of chloroquine and primaquine were measured by high-performance liquid chromatography (HPLC; [21]) two weeks prior to CHMI, to ensure drug clearance. Eight weeks after the last immunization, and one month after completing antimalarial treatment, all volunteers received CHMI using 2–4 *Pv*-infected mosquito bites. Physical examination, clinical laboratory, and immunological tests were performed after every immunization and CHMI (Fig 2). Adverse events (AE) were recorded, graded and classified according to FDA recommendations [22].

Whole blood was collected by venipuncture of the arm at inclusion (baseline), ten days after each immunization, before CHMI, and six months post-CHMI for clinical laboratory and immunological tests. After each immunization, volunteers were followed-up on days 1, 2 and 10 in person for a physical examination and by phone on days 7 and 14. Likewise, after CHMI, volunteers were followed up every day by phone until day 5 and malaria infection monitored daily in an outpatient clinic from day 6–28 post-CHMI; thereafter twice a week from day 29–60 post-CHMI for volunteers who did not develop fever or patent infection within 28 days post-CHMI as determined by TBS microscopy and qPCR. Additionally, volunteers were encouraged to visit the Centre for medical consultation at any moment if they developed any symptom or had any concern. Treatment was initiated immediately after parasitemia was confirmed by TBS and the volunteers followed-up until three consecutive TBS resulted negative. Afterward, volunteers had TBS assessed on days 7, 14, 21 and 45 post-treatment to confirm cure and absence of relapse [15–17]. Serum and plasma were stored at -20°C until use. Peripheral blood mononuclear cells (PBMC) obtained by Ficoll density gradient centrifugation were stored in liquid nitrogen until use.

Vaccine efficacy was assessed by prevention of patent parasitemia. Infection was diagnosed by TBS examination by two independent experienced microscopists, and parasitemia determined by counting the number of asexual *Pv* parasites per 400 white blood cells (WBC), assuming normal WBC counts (8,000 cells/μL). Samples were considered negative after observation of 200 microscopic fields and qPCR was performed subsequently for retrospective analyses. Clinical laboratory tests were periodically performed during immunizations and as required by clinical judgment after the CHMI to ascertain health status (same methods as recruitment screening tests, S1 Table).

### Antibody response

A secondary outcome was the evaluation of humoral immune responses. Specific antimalarial antibodies (Ab) were determined by enzyme-linked immuno-sorbent assay (ELISA). The presence of IgG to *Pv*CS (NRC and N peptides) and to merozoite surface protein-1 (*Pv*MSP-1) was assessed in sera diluted 1:200 as previously described [17]. *Pv*CS corresponded to a chimeric synthetic polypeptide composed of the amino (N) flank, the VK210 and VK247 natural repeat variants (R), and the carboxyl (C) flanking sequences of the protein [23]; *Pv*MSP-1 corresponded to a recombinant fragment from the N region of the protein, namely r200L [24]. IgG isotypes against *Pv*CS-NRC peptide were detected using mouse monoclonal Abs to specific to human IgG1, IgG2, IgG3 and IgG4 (Sigma-Aldrich), followed by HRP-conjugated anti-mouse. In all cases, the optical density (OD) was measured using a BioTek ELISA Reader (BioTek, Winooski, VT). Cut-off values were calculated as three SD above the mean OD value of negative control sera. Results were expressed as reactivity index (RI), defined as optical density (OD) values of test sample divided by the cut-off value. Immunofluorescence tests (IFAT) were used to assess the Ab reactivity with *Pv*SPZ.

### Ex-vivo Interferon-γ (IFN-γ) ELISpot

To determine the frequency of T cells responding to *P*. *vivax* antigens, IFN-γ production was quantified using an ELISpot assay. Briefly, the assay was performed in multiscreen 96-well plates (MAHAS 4510, Millipore) coated with anti-human IFN-γ capture antibody (1-D1K; Mabtech AB). Fresh PBMC collected 12 days previous to the CHMI were plated into duplicate wells at 4 x 10^5^ cells in complete RPMI-1640 medium (cRPMI; Gibco, Invitrogen) supplemented with 10% FBS. The PBMC were stimulated for 40 h at 37°C with 10 μg/mL of *Pv*SPZ lysate, *Pv*CS-NRC or *Pv*TRAP (thrombospondin-related adhesive protein). cRPMI medium-only and PHA controls were used in all assays. Biotinylated anti-IFN-γ antibody (7-B6-1; Mabtech AB) was added followed by alkaline phosphatase-streptavidin conjugate (Mabtech AB). Spots were visualized by adding BCIP/NBT (Sigma-Aldrich), scanned and counted using the AID ELISpot reader (AID Autoimmun Diagnostika GmbH, Germany) to determine the number of spots/well. Results were expressed as spots per 10^6^ PBMC, normalized by the antigen-stimulated spots less cRPMI medium.

### Statistical analysis

Data were collected and managed using REDCap (Nashville, TN, USA) electronic data capture tools, analyzed using SPSS version 16.0 software (SPSS Inc., Chicago, IL, USA), and plotted using GraphPad Prism version 6.0 (GraphPad Software, San Diego, California, USA). We estimated a sample size of 21 Fy+ individuals (2:1, RAS to Ctl) at a 5% significance level and 80% power to assess the protective efficacy of immunization. Nominal variables were analyzed using descriptive statistics. Mann-Whitney U or the Kruskal-Wallis tests were used as needed. Fisher's exact test was used to compare proportions. Spearman’s rank correlation (r_s_) was used to assess the correlation between numeric variables. Incubation and pre-patent periods were determined by TBS and qPCR and visualized using Kaplan–Meier estimator. A p value < 0.05 was considered statistically significant.

## Results

### Immunization schedule

A total of 28 of the screened volunteers were enrolled and began the immunization schedule between Sept 26, 2013, and Feb 15, 2014. However, only 19 completed the schedule (Fig 1 and Fig 2). Mean age at enrollment was 30, 29 and 25 years, and the male/female ratio was 5:9, 5:2, 0:7 for the RAS, Ctl, and Fy- groups, respectively (Table 1).

**Table 1:** Baseline characteristics of volunteers, total dose of received mosquito bites and CHMI results.

Group	Code	Gender	Age at enrollment	Number of immunizations	Total number of bites^a^	Infected after the CHMI	Incubation period (days)	Pre-patent period (days)		Parasite density at diagnosis (parasites/μL)	
								TBS	qPCR	TBS	qPCR
RAS											
	001^b^	F	24	7	440	No	58	66	66	2000	12300
	005	M	30	7	418	Yes	9	13	11	110	1,050
	006	M	40	7	497	Yes	13	13	8	425	233
	007	F	21	7	362	No	-	-	-		
	008	F	35	3	164	WBC	-	-	-		
	009	F	33	7	458	Yes	11	13	10	80	14
	010	F	25	7	460	No	-	-	-		
	011	M	38	7	423	Yes	10	13	8	400	361
	012	F	37	7	428	No	-	-	-		
	013	F	24	4	314	WBC	-	-	-		
	017	M	35	7	386	Yes	9	13	9	655	29
	021	M	22	7	442	Yes	9	12	8	179	120
	025	F	21	7	403	No	-	-	-		
	026^c^	F	36	7	440	Yes	-	12	8	145	220
Ctl											
	002^d^	M	27	7	758	Excluded	-	-	-		
	003	F	28	4	557	WBC	-	-	-		
	004	M	23	4	534	WBC	-	-	-		
	015	M	39	7	895	WBC	-	-	-		
	020	M	41	7	945	Yes	10	13	8	2,950	12.5
	049	F	22	3	385	WBC	-	-	-		
	065	M	23	7	963	Yes	8	13	11	80	1,042
**Fy-**											
	038	F	24	7	478	No	-	-	-		
	058	F	21	7	487	No	-	-	-		
	062	F	19	4	261	WBC					
	066	F	37	7	358	No	-	-	-		
	069	F	28	4	292	WBC					
	075	F	19	7	476	No	-	-	-		
	084	F	25	7	412	No	-	-	-		

^*a*^RAS group: number of bites from infected-irradiated mosquitoes; Ctl group: non-infected and non-irradiated mosquitoes; and, Fy- group: infected and non-irradiated mosquitoes.

^*b*^Volunteer 001 developed patent parasitemia at day 66, after the follow-up had been finished.

^*c*^Volunteer 026 developed patent parasitemia by TBS but developed malaria symptoms only after antimalarial treatment.

^*d*^Volunteer 002 was erroneously immunized with 41 RAS containing mosquitoes in the fifth immunization and was excluded.

Abbreviations: RAS, radiation-attenuated sporozoites; Ctl, control; Fy, Duffy; F, female; M, male; WBC, withdrew before the CHMI; TBS, thick blood smear.

**Fig 3:**
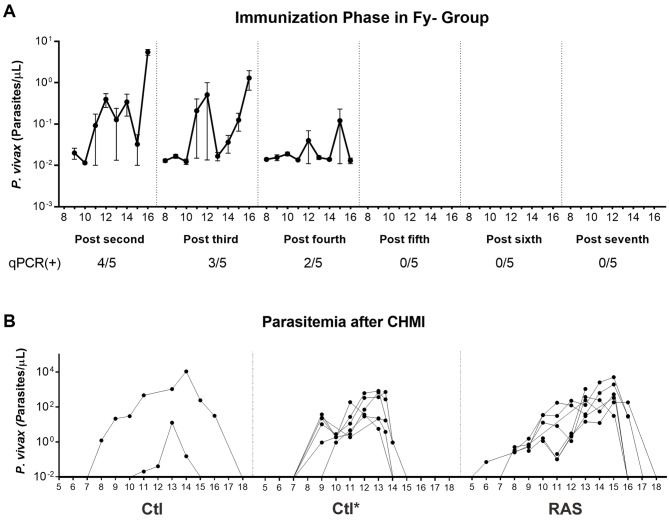
Parasitemia determined by qPCR. **A**. Number of parasite DNA copies per μL determined during the immunization phase in the Fy- group. **B**. Parasitemia after the CHMI in Ctl and RAS groups. Ctl* corresponds to the parasitemia dynamics determined by qPCR during a previous CHMI experiment that included naïve Fy+ individuals using the same procedures for comparison. Each point represents mean ± SEM of parasites/μL (Log_10_).

A total of seven immunizations were carried out at mean intervals of nine weeks (range 3–25 weeks) in volunteers who then continued to complete the CHMI. The RAS and Fy- groups received a median of 434 (range 362–497) and 476 (range 358–487) total infective bites over the seven immunizations, respectively, whereas the Ctl group received 954 (range 945–963) non-infective (placebo) bites during the immunization protocol. The total number of infective bites, non-infective bites, fed mosquitoes, and spz in salivary glands per volunteer were determined by post-feeding salivary gland dissection and microscopy examination (S3 Table). No volunteer developed clinical malaria or parasitemia by TBS during the immunization phase, although low levels of parasite DNA were detected in peripheral blood by qPCR from day 8–16 after immunizations in the Fy- group, which declined after every subsequent immunization (Fig 3A). At the time of the CHMI, all volunteers had cleared both primaquine and chloroquine in plasma, although two volunteers in the RAS group had low detectable levels of chloroquine two weeks prior to the CHMI. Notably, both volunteers developed malaria infection.

### Clinical follow-up and adverse events (AE)

**Fig 4:**
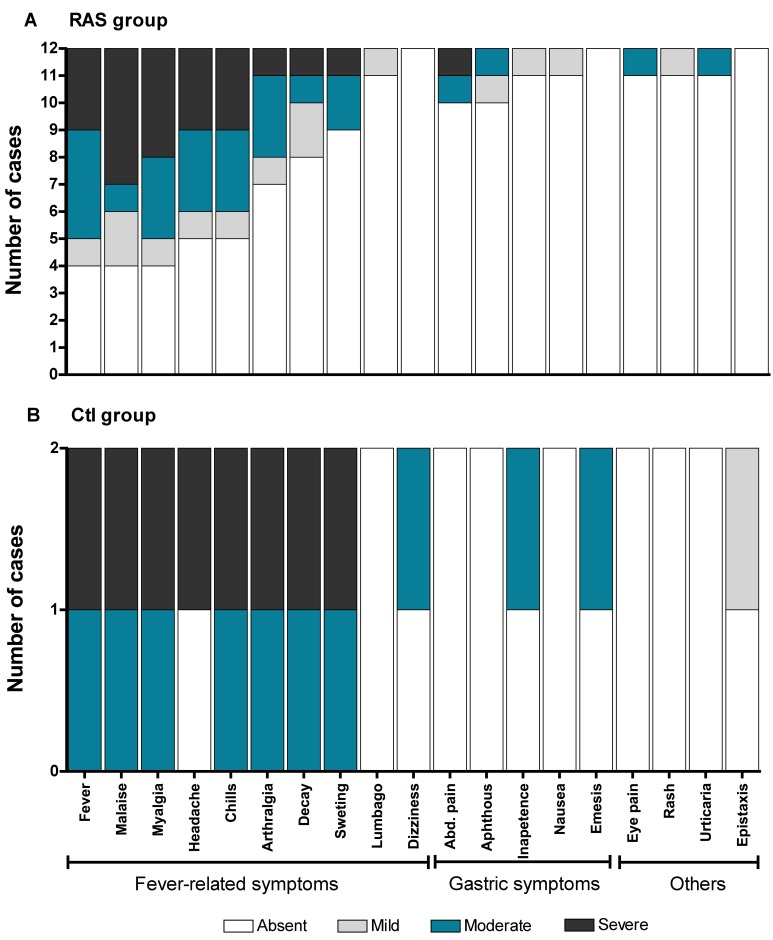
Frequency and intensity of adverse events after the CHMI. The adverse events graded according to FDA recommendations [22] and grouped as fever-related symptoms; gastric symptoms; and, others in RAS (n = 12; **A**) and Ctl group (n = 2; **B**) are shown. No AE after the CHMI were observed in the Fy- group (n = 5). Abbreviations: Abd, abdominal pain; rash, generalized rash; aphthous, aphthous stomatitis.

Seven to nine days after the first immunization, 1/14 and 5/7 volunteers of the RAS and Fy- groups, respectively, developed fever, chills, headache and profuse sweating consistent with malaria, which lasted 1–2 days. All five symptomatic Fy- volunteers had negative TBS but positive qPCR that resolved spontaneously, whereas the RAS volunteer was negative by TBS and qPCR. Headache and local reaction in the immunization site were the most common AE during initial immunizations with decreasing frequency throughout the immunizations (S4 Table). After CHMI headache, chills, fever, and malaise were common AE (Fig 4). In the RAS and Ctl groups, a mean of 11 and 16 AE per individual were reported after CHMI, respectively. In contrast, in the Fy- group a mean of two AE was reported. No serious AE related to immunizations were observed, although one female developed severe elevation of hepatic transaminases after CHMI (>10 times upper limit of normal [xULN]) and lactic dehydrogenase (2.5xULN) with abdominal pain, nausea, and vomiting during *Pv* malaria mono-infection. This patient was observed in the emergency room and completely recovered without sequelae. No alternative etiologies for the elevated transaminases were identified (volunteer was negative for hepatitis C, hepatitis B, HIV and hepatitis A, and she was not consuming any medications).

At day 60 post-CHMI (last day of follow-up), the total protective efficacy in the RAS group was calculated at 42% (5/12 RAS, 0/2 Ctl) as determined by TBS and confirmed by qPCR; all protected subjects were women (Table 1). All malaria-positive volunteers presented with low parasitemia, with median density values lower by TBS than by qPCR (TBS: 140 parasites/μL; IQR 95–210, and qPCR: 220 parasites/μL; IQR 29.2–361). Mean incubation period was 9.9 days (range 8–13); mean prepatent period was 12.8 days (range 12–13) by TBS; and 9.0 days (range 8–11) by qPCR. No significant differences were found between the Ctl and positive RAS subjects in prepatent period or density of parasitemia by TBS or qPCR (Fig 3B). However, survival analysis showed a significantly greater incubation period in RAS than in Ctl volunteers (S1 Fig). These results were compared with the parasite dynamics of a previous CHMI trial carried out in naïve volunteers using the same infection protocol. Those volunteers who did not develop malaria were followed up until day 60 post-CHMI after which antimalarial treatment was administered. Volunteer 001 of the RAS group developed malaria-related symptoms at day 58 post-CHMI, but parasitemia was only detected on day 66 by TBS. This prepatent period was considered as partial protection induced by vaccination.

### Antibody response

**Fig 5:**
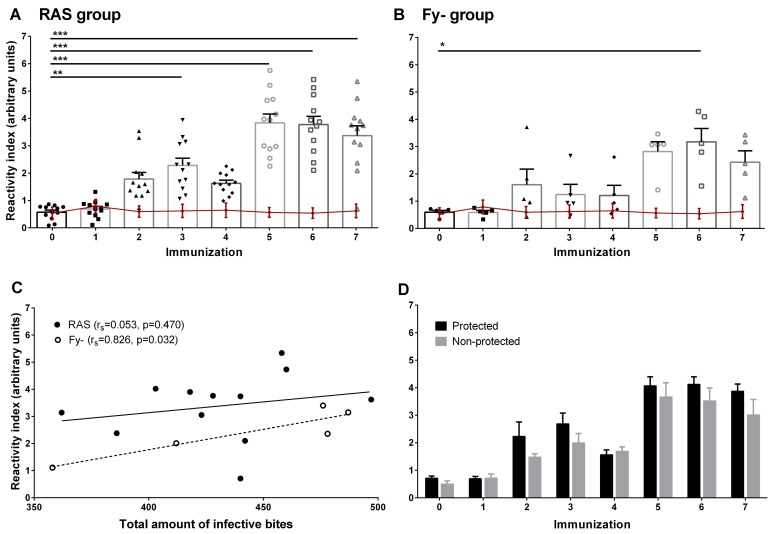
Antibody response against *Pv*CS*-*NRC peptide. Total IgG response determined by ELISA in the RAS group (n = 12; **A**), Fy- group (n = 5; **B**) and Ctl group (n = 2; red line in **A** and **B**). Values are expressed as reactivity index (RI) defined as sample OD at 1:200 serum dilutions divided by the cut-off value. Mean ± SEM are shown. **C**. Correlations between total received dose of infective bites and RI at seventh immunization for RAS and Fy- volunteers. Spearman’s rank correlation (r_s_) and p values are shown. **D**. Mean ± SEM of RI for protected and non-protected volunteers after every immunization. * p < 0.05; ** p < 0.01; *** p < 0.001.

**Fig 6:**
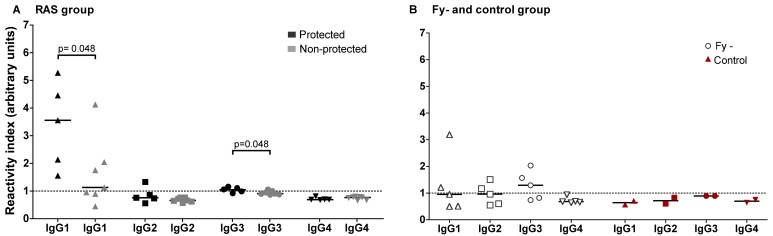
IgG isotype response against *Pv*CS*-*NRC peptide. Antibody IgG isotype levels determined by ELISA in the RAS group (n = 12; **A**), Fy- group (n = 5; **B**) and Ctl group (n = 2; **B**) at seventh immunization are shown. Values are expressed as reactivity index (RI) defined as sample OD divided by the cut-off value. Horizontal bars indicate median values. p value using the Mann-Whitney U test between protected and non-protected are shown.

Seroconversion using the *Pv*CS-NRC peptide was observed in all 12 RAS volunteers, mostly after the second immunization (10/12) and in all Fy- volunteers between the second and fifth immunizations. In both groups, IgG reactivity was low (RI < 6); all Ctl volunteers remained seronegative during the immunization phase (Fig 5A and 5B). A positive correlation between the RI for *Pv*CS-NRC and number of infective bites was observed for the Fy- group but not for the RAS group (Fig 5C). No significant association between total anti *Pv*CS-NRC RI and protection was found (Fig 5D); however, the specific IgG1 response was significantly higher in protected individuals (Fig 6A and 6B). All Fy- volunteers and one in RAS group developed anti-*Pv*MSP-1 IgG response after seven immunizations. In contrast, all Ctl volunteers remained negative for all antigens tested (S2 Fig). After immunization, 11/12 of RAS and 4/5 of Fy- volunteers had IFAT Abs to *Pv* spz, respectively, but no association with protection was found (S5 Table). Moreover, all RAS and Fy- sera recognized *Pv*CS by Western blot (S3 Fig).

### Ex-vivo IFN-γ production

**Fig 7:**
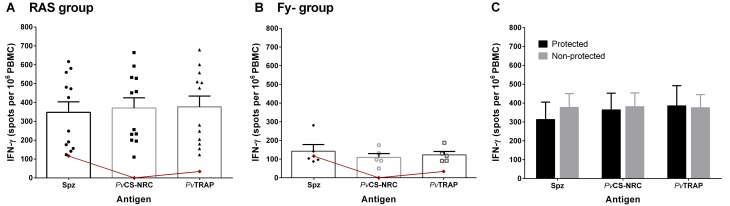
IFN-γ production to individual malaria antigens before the CHMI. Ex-vivo IFN-γ ELISpot responses in the RAS group (n = 12; **A**), Fy- group (n = 5; **B**), and Ctl group (n = 2; red line in **A** and **B**) previous to the CHMI. PBMC were stimulated with *Pv* spz lysate, *Pv*CS-NRC, and *Pv*TRAP. Mean ± SEM are shown. **C**. Mean ± SEM of spots per 10^6^ PBMC for protected and non-protected volunteers.

After seven immunizations and before to CHMI, PMBCs of the RAS group were able to produce IFN-γ after stimulation with the tested antigens *Pv* spz lysate, *Pv*CS-NRC, and *Pv*TRAP (Fig 7A) at significantly higher levels than the other two groups (p <0.05 for all antigens). In the Fy- group, *Pv*CS-NRC and *Pv*TRAP induced IFN-γ production but was not significantly higher than the observed in the Ctl volunteers (Fig 7B). No significant differences were observed between protected and not protected volunteers in the RAS group (Fig 7C).

## Discussion

This trial has allowed the establishment of the *Pv*RAS immunization model with protection in an unprecedented number of volunteers. To our knowledge, only two volunteers had been previously reported to be protected from CHMI by *Pv*RAS immunization [12]. As is true for *Pf*RAS, immunization by mosquito bite with the *Pv*RAS is safe, immunogenic and able to induce sterile protection. A series of clinical trials conducted with *Pf*RAS indicated high protective efficacy (~90%) and protection lasting up to 42 weeks with a dose-dependent efficacy based on ten immunization sessions and a total of ~1000 RAS mosquito bites [3–5, 8, 9, 12]. This study could not reproduce those conditions due to the difficulty of obtaining *Pv*RAS, which include the need of regular *P*. *vivax* infected donors from malaria endemic areas, willing to participate and complying with all inclusion criteria. In addition, not all *P*. *vivax* samples are successfully infective to *An*. *albimanus* mosquitoes due to numerous biological factors [25]. Nevertheless, seven immunization sessions provided a median of 434 *Pv*RAS bites for an efficacy of 42%. This is similar to what has been found with *Pf* immunization, where the protective efficacy against CHMI in volunteers receiving < 1000 infectious bites was 40% [9]. Despite the high number of volunteers that withdrew from the Ctl group, the two remaining showed a trend in the parasite dynamics similar to that observed in a total of 29 naïve Fy+ volunteers infected with 2–4 *An*. *albimanus* mosquito bites in three previous CHMI trials [15–17]. In one of these trials [17], even semi-immune volunteers from endemic areas previously exposed to natural malaria infection developed similar parasite patency, indicating the relevance of the sterile protection induced here by *Pv*RAS immunization. Therefore, given that both controls turned positive following CHMI, we are confident that *Pv*RAS immunization induced sterile protection as described in RAS group. A summary of parasitological data for naïve volunteers participating in previous CHMI carried out in our Centre, which demonstrate the consistency of this procedure, is shown (Table 2). Since no detectable levels of chloroquine were observed in most of RAS volunteers, and the two volunteers with low detectable levels had undetectable levels immediately previous to CHMI and developed malaria infection, we concluded that lack of parasitemia was not dependent of chloroquine antiplasmodial activity.

**Table 2:** Naïve Fy+ volunteers participating in previous CHMI carried out at the MVDC.

Study and group^*a*^	Code	Gender	Age	Mosquito bites^*b*^	Pre-patent period (days)^*c*^		Parasite density (parasites/μL)
					TBS	PCR	
**CHMI 1 [15]**							
	A: 3 ± 1 infective bites (n = 6)						
	206	F	41	4	11	11	152
	207	F	22	4	9	9	298
	208	M	32	4	9	9	144
	221	F	23	4	12	12	93
	222	F	46	2	13	10	280
	226	F	20	4	13	13	56
**CHMI 2 [16]**							
	Isolate A, 3 ± 1 infective bites (n = 6)						
	1	F	28	2	13	ND	160
	2	M	40	2	15	ND	160
	4	M	21	2	16	ND	160
	5	M	20	2	13	ND	160
	6	M	27	4	12	ND	480
	8	F	19	2	15	ND	400
	Isolate B, 3 ± 1 infective bites (n = 6)						
	1	F	43	3	10	ND	160
	2	M	21	2	16	ND	80
	3	M	33	2	10	ND	80
	4	M	31	2	10	ND	80
	6	M	32	2	9	ND	80
	7	M	18	3	10	ND	160
	Isolate C, 3 ± 1 infective bites (n = 5)						
	1	M	41	3	12	ND	160
	2	F	25	3	10	ND	80
	3	M	24	3	10	ND	80
	4	M	26	2	10	ND	80
	6	F	23	2	12	ND	320
**CHMI 3** [17]							
	Malaria-naïve, 3 ± 1 infective bites (n = 7)						
	302	M	29	4	13	10	34
	304^*d*^	M	26	2	NA	NA	NA
	306	M	38	3	13	9	95
	310	M	31	3	13	9	110
	314	F	34	4	12	9	10
	317	M	33	4	11	9	6
	319	M	22	4	13	9	38

^*a*^ CHMI 1 aim: determine the minimal effective doses of infective mosquito bites required to cause clinical malaria. CHMI 2 aim: assess the reproducibility of the experiment using *P*. *vivax* isolates from different donors. CHMI 3 aim: compare the reproducibility, effectiveness, and clinical outcomes in naïve and semi-immune individuals.

^*b*^ Number of infective mosquitoes bites.

^*c*^ Parasitemia measured at the pre-patent day.

^*d*^ volunteer remained negative for malaria by TBS during the duration of the study.

TBS, thick blood smear; PCR, polymerase chain reaction; NA, Not applicable; ND: no data.

Significant progress has been achieved regarding the development of a practical approach for *Pf* immunization based on whole SPZ [7]. Intravenous administration of aseptic, purified, cryopreserved, radiation-attenuated *Pv*SPZ [7] has shown the highest efficacy, protecting up to 100% of study subjects. Based on the results of our study, we can anticipate that a similar *Pv* product would be equally protective, and could potentially be combined with *Pf*spz to induce potent immunity to the two major species of human malaria. The use of the whole SPZ approach may therefore be an effective route to solving the malaria problem, given that subunit vaccines appear to have a longer development trajectory.

The RTS,S vaccine, the most advanced *Pf* malaria subunit vaccine, has been assessed as meeting EMA standards, setting the stage for potential licensure in African countries, and has subsequently been recommended by WHO for testing in pilot implementations in Africa [2]. Several other vaccine candidates are also under development [26]. RTS,S is to be licensed for reducing the incidence of clinical malaria but not preventing malaria infection, and is insufficiently potent for use in elimination campaigns. Progress in subunit vaccines for *Pv* has been especially limited due to the lack of an *in vitro* culture method and the scarce funding. In order to fill this gap, we approached the development of *Pv*RAS by accessing to fresh gametocytemic blood from patients to assess the *Pv*RAS model’s feasibility and reproducibility under controlled conditions, and to generate immune reagents to determine correlates of protection.

Additionally, this study took the novel approach of immunizing Fy- volunteers by repeated exposure to viable *Pv*SPZ. Because most Fy- individuals are refractory to blood infection by *Pv*, this allowed evaluation of immune responses elicited specifically against liver-stage parasites. Although there have been reports from Madagascar and Cameroon-endemic areas that some of these subjects may develop the blood cycle when infected by *Pv* [27, 28], this did not happen in our study with different natural parasite isolates. To our knowledge, this was the first time that Fy- volunteers have been used as a model for a better understanding of the immune responses to *Pv* liver stages. The presence of symptoms in Fy- following only the first immunization, and the diminishing qPCR positivity as immunizations continued, indicate that the Fy- volunteers developed sterile immunity to *Pv* infection based on immunity targeting the pre-erythrocytic stages.

Reagents generated in this study allow the use of both classic and high throughput methods to analyze the immune response to *Pv*RAS, and comparison of responses to the early liver stages in the *Pv*RAS and Fy- groups. Sera and cells are currently being studied using high throughput systems in an attempt to determine correlates of immune protection.

Interestingly, all protected volunteers were women, whereas all men developed malaria despite receiving similar parasite doses. No covariates, such as numbers of immunizing bites, were identified to explain this finding. This is consistent with other studies where women mounted a more vigorous immune response than men (reviewed in [29]), although this was not evident here at least for the parameters evaluated, which may or may not serve as correlates of protection. We achieved the doses necessary to protect almost all challenged women (5/7) but not men (0/5).

Immunization with both *Pv*RAS and viable *Pv* spz induced a measurable although weak ELISA antibody response to *Pv*CS, and there was no association between total IgG Ab levels to *Pv*CS and protection. Nonetheless, the protected volunteers had a greater IgG1 response against *Pv*CS-NRC peptide, which is in agreement with studies describing associations between higher levels of IgG1 and IgG3 Abs and protection against severe *Pf* malaria episodes [30] as well as predominant markers for exposure to *Pv* malaria [31]. However, the borderline p value (p = 0.048) for the association between *Pv*CS-NRC peptide ELISA titer and protection was possibly due to the relatively small number of individuals tested. All Fy- individuals and only one *Pv*RAS developed Ab levels against *Pv*MSP-1 protein after seven immunizations. This appears to be in agreement with the fact that *Pv* is able to completely develop the liver cycle and release merozoites into circulation in Fy- volunteers as demonstrated by qPCR, whereas RAS appears to arrest development in early phases of the liver cycle [32]. It is also consistent with the fact that Fy- volunteers developed fever and other malaria symptoms during the first immunizations and the fact that parasite DNA was detected up to the fourth immunization. The decrease in anti-CS Ab levels after the third and fourth immunizations, when there was a pause of several months in immunizations, indicates that these Abs are short-lived, although memory cell responses were present as demonstrated by the rapid boosting of specific Abs after the fifth immunization.

This trial confirms the reproducibility of the RAS vaccination model in *Pv* malaria. Despite the lack of correlation between protection and the tested immune responses, high throughput analyses of cells and sera, i.e., transcriptomics and anti-parasite Ab microarray profiles, may offer a better understanding of the parasite targets involved and the immune effector mechanisms associated with protection.

## Supporting Information

S1 Checklist CONSORT checklist. (DOC)

S1 Protocol (PDF)

S1 Fig Kaplan-Meier analysis of pre-patent and incubation period after *P*. *vivax* sporozoite CHMI. Days after CHMI to detect parasites by microscopy (**A**) or RT-qPCR (**B**) and onset of symptoms (**C**).
(TIF)

S2 Fig Antibody response against *Pv*CS-N peptide and *Pv*MSP-1. ELISA antibody response in RAS group (n = 12; **A** and **C**) and in Fy- group (n = 5; **B** and **D**) as well as in Ctl group (n = 2; red line in **A** to **D**) against *Pv*CS*-*N (**A-B**) and *Pv*MSP-1 (**C-D**) are shown. Values are expressed as reactivity index (RI) defined as sample OD at 1:200 serum dilutions divided by the cut-off value. Mean ± SEM are shown.
(TIF)

S3 Fig *Plasmodium vivax* CS protein recognized by immunized volunteers. Western blot analysis of *P*. *vivax* sporozoites lysate separated on 12% SDS-PAGE under non-reducing conditions. Sera from RAS group (n = 12), Fy- group (n = 5), and Ctl group (n = 2) are shown. Negative (naïve volunteers) and positive (volunteers immunized with *Pv*CSP) controls are also shown. Relative size standards are indicated on the left in KDa.
(TIF)

S1 Table Clinical and laboratory exclusion criteria, assessment techniques and excluded volunteers. (DOC)

S2 Table Screening of common infectious agents for recruited volunteers. (DOC)

S3 Table Total number of mosquito bites received during immunizations. (XLSX)

S4 Table Adverse events during immunizations and after CHMI. (XLS)

S5 Table Indirect immunofluorescence using *Plasmodium vivax* sporozoites. (DOC)

S1 Text Additional methods. (DOC)
