# Supplementary material for: Protective Efficacy of Plasmodium vivax Radiation-Attenuated Sporozoites in Colombian Volunteers: A Randomized Controlled Trial
Source: PLoS Negl Trop Dis. 2016 Oct 19;10(10):e0005070. doi: 10.1371/journal.pntd.0005070 (PMC5070852; doi:10.1371/journal.pntd.0005070)
Supplement: S1 Protocol — (PDF) [file pntd.0005070.s002.pdf]

Phase 1 and 2a Clinical Trial: Immunization of human volunteers with *P. vivax* irradiated sporozoites

Protocol CIV – 01 – 042009

Sponsored by:
National Heart, Lung and Blood Institute (NHLBI, NIH, USA)
DMID Funding Mechanism: R01HL 086488 A

Principal Investigator
Sócrates Herrera Valencia, MD

Version Number: 5.0

15th October 2014

STATEMENT OF COMPLIANCE

The study will be carried out in compliance with Good Laboratory Practices (GLP) as required by the ISO 9000, 2001 guidelines. This study will be approved by the Institution Review Board (IRB) of the MVDC (CECIV; Comité de Ética Centro Internacional de Vacunas). This protocol contains informed consent forms (Appendix 1) that include the information about the guarantees for the volunteers participating in the study.

Volunteer's recruitment and study activities will begin after approval of the protocol by the local IRBs and NHLBI/NIH. All aspect of the protocol involving human subject participation will be carried out in accordance to the NHLBI clinical terms of awards, and the ICH/GCP guidelines.

SIGNATURE PAGE

The signatures below constitute acknowledgment of the protocol and the attachments, and provide the necessary assurances that this clinical study will be conducted according to all stipulations of the protocol, including all statements regarding confidentiality and according to local legal and regulatory requirements and to the principles outlined in applicable ICH guidelines.

Principal Investigator – *Name of Site*:
Signed: Date: Oct, 15th , 2014

Sócrates Herrera
MD

Table of Contents

LIST OF ABBREVIATIONS **5**

Protocol Summary **6**
Schematic overview of the study design **7**

1. Key Roles **11**

2. Introduction **13**
2.1 Background Information and Scientific Rationale **13**
2.1.1 Current significance of the proposed model. 13
2.1.2 Former studies on malaria irr-spz models. 13
2.1.3 Sporozoite challenge system and testing the irr-spz vaccine. 14
2.1.4 Studies on the immune response induced by irradiated sporozoite. 15
2.1.5 Significance of a reproducible *P. vivax* spz challenge model and advantages for conducting research in an endemic area. 16

3. Study hypothesis **17**

4. Potential risks and benefits **17**
4.1 Potential Risks to volunteers immunized with irradiated spz and challenge **17**
4.2 Known potential benefits to volunteer **17**
4.3 Potential risks to infected blood donors **18**
4.4 Known Potential Benefits to infected blood donors **18**

5. Objectives **18**
5.1 Primary **18**
5.2 Secondary **18**

6. Study Design **19**
6.1 Steps of the study **19**
6.1.1 Step 1, Parasite infected blood donation 19
6.1.2 Step 2, Sporozoite immunization 20
6.1.3 Step 3, Sporozoite challenge 20

7. Study population **20**
7.1 Selection and recruitment procedures of participants **20**
7.1.1 Step 1. Parasite infected blood donation. 20
7.1.2 Step 2 and 3. Sporozoite immunization and challenge 21

8. Enrollment/randomization/masking procedures **22**
8.1 Step1 Enrollment Procedures **22**
8.2 Step 2 and 3 Enrollment, Allocation and Masking Procedures **22**

9. Study Procedures/Evaluation **23**

9.1 Step 1. Parasite infected blood donation 23
9.1.2 Preparation of sporozoites 25
9.1.3 Criteria to excluded batches to volunteers' immunization 26
9.1.4 Irradiation 26
9.2 Study Procedures for steps 2 and 3 26
9.2.1 Step 2. Irradiated sporozoite immunization 26
9.2.2. Malarial treatment after immunization 30
9.2.3 Last follow-up before step 3 30
9.3 Study Procedures for step 3 30
9.3.1 Step 3. Challenge to infected mosquitoes 30
9.3.2 Post-challenge evaluation 30
9.3.3 Malarial treatment 31
9.3.4 Follow up after initiation of antimalarial treatment 31
9.3.5 Follow up for relapses or recrudescence 31
9.4 Proposed phone follow-up post treatment 32

10 Clinical Evaluations 32
10.1 Clinical evaluations for Step 1 32
10.1.1 Recruitment 32
10.1.2 Follow-up after malarial treatment 33
10.2 Clinical evaluations for Step 2 33
10.2.1 Recruitment 33
10.2.2 Pre-immunization 33
10.2.3 Immunization 33
10.2.4 Malaria treatment after immunization 34
10.3 Clinical evaluations for Step 3 34
10.3.1 Pre-Challenge 34
10.3.2 Challenge 34
10.3.3 Post-challenge follow up 34
10.3.4 Follow up after antimalarial treatment for infection 34
10.3.5 Proposed phone follow-up post treatment 34
10.3.6 Concomitant Medications/Treatments 35

11 Laboratory Evaluations 35
11.1 Specimen Collection, Preparation, Handling and Shipping 35
11.1.1 Step 1: parasite donors 35
11.1.2 Step 2: immunized volunteers 35
11.1.3 Step 3 36

12 Assessment of scientific Objectives 36
12.1 Specification of the appropriate outcome measures 36

12.1.1 Primary outcome measurement 36
12.1.2 Secondary outcome measures 37
12.2 Methods and timing for assessing, recording and analyzing outcome measures ... 37
12.2.1 Protective efficacy measurement 37
12.2.2 Safety evaluation 37
12.3 Modification and discontinuation of study intervention product for a participant 38

13 Assessment of safety 38
13.1 Adverse events (AE) 38
13.2 Serious adverse event (SAE) 39

14 Reporting procedures 39
14.1 Serious adverse event detection and reporting 39
14.2 Reporting of subject death 39
14.3 Procedures to be followed in the event of abnormal findings 39
14.4 Type and duration of the follow-up of subjects after AE 40
14.5 Reporting of pregnancies 41

15 Clinical monitoring structure 41
15.1 Site monitoring plan 41

16 Statistical considerations 41
16.1 Overview and study objectives 41
16.2 Study population 41
16.3 Study outcome measures 41
16.4 Sample size considerations 41
16.5 Participant enrollment and follow-up 44
16.6 Variables 44
16.6.1 Dependent variables 44
16.7 Independent variables 44
16.8 Covariables 44
16.9 Analysis plan 45

17 Potential pitfalls 46

18 Ethics/protection of human subjects 47
18.1 Declaration of Helsinki 47
18.2 Institutional review board 47
18.3 Informed Consent Process 47
18.4 Subject Confidentiality 48
18.5 Future use of stored specimens 48

19 Data handling and record keeping 49
19.1 Data Collection 49

19.2 Data entry **49**
19.3 Data queries **49**
19.4 Timing/Reports **50**
19.5 Study records retention **50**

20 Protocol Deviations **50**

21 Publication policy **50**

22 References **50**

LIST OF ABBREVIATIONS

AE	Adverse events
βHCG	β-human chorionic gonadotropin (pregnancy test)
CBC	Complete blood count
CECIV	IRB of the MVDC (Comité de Ética Centro Internacional de Vacunas)
CIH	International Conference on Harmonization
CRF	Case Report Form
DPB	Duffy binding protein
Fy	Duffy blood group
GCP	Good Clinical Practice
ICF	Informed Consent Form
IFAT	Indirect Immunofluorescence antibody test
IFN-g	Interferon gamma
Irr-spz	Irradiated-sporozoites
IRB	Institutional Review Board
MFA	Membrane Feeding Assay
MVDC	Malaria Vaccine and Drug Development Center
N	Number (typically refers to subjects)
MVDC	Malaria Vaccine and Drug Development Center
N	Number (typically refers to subjects)
NHLBI	National Heart, Lung and Blood Institute
NIAID	National Institute of Allergy and Infectious Diseases, NIH, DHHS
NIH	National Institutes of Health
PBMC	Peripheral blood mononuclear cell
PCR	Polymerase chain reaction
PI	Principal Investigator
PvCS	*Plasmodium vivax* Circumsporozoite protein
PvMSP-1	*Plasmodium vivax* merozoite surface protein
RBC	Red Blood Cells
SOP	Standard Operating Procedure
SPZ	sporozoites
TB	Transmission Blocking
TBS	Thick blood smear
TMRC	Tropical Medicine Research Center
WRAIR	Walter Reed Army Institute for Research
WHO	World Health Organization

Protocol Summary

Phase 1 and 2a Clinical Trial: Immunization of human volunteers with *P. vivax* irradiated-sporozoites (Short name: irradiated-SPZ)

**Study population**	28 healthy adult volunteers, males and non-pregnant females, between 18-45 years of age, who fulfill inclusion and exclusion criteria (described below) as determined by clinical history and serological tests. Donor parasites volunteers will be required to infect *Anopheles* female mosquitoes. Volunteers will be immunized via bites from irradiated *P. vivax* infected mosquitoes (500 infected mosquitoes during 10-12 months). After this period of time, volunteers received a challenge with non-irradiated *P. vivax* infected mosquitoes to study protective efficacy due to the vaccination.
**Number of sites**	2
**Study duration**	2 years
**Subject duration**	Step 1: 1-2 hours for blood donors.<br>Step 2: 2 years for immunized volunteers.
**Study objectives**	**Primary objective**<br>To assess protective efficacy and safety of *P. vivax* irradiated sporozoite vaccination.<br><br>**Secondary objectives**<br>• To determine the immune response and duration elicited by the *P. vivax* challenge in human Fy(+) volunteers previously immunized with irr-spz, as compared with non-immunized human volunteers.<br>• To determine the immune response and duration elicited by irr-spz immunization in human Fy(+) volunteers; comparing it with the same immunization scheme but with non irr-spz in Fy(-) participants, and also with controls exposed to the same mosquito bite scheme as that of immunization but without parasite infection.<br>• To study new antigens potentially useful for pre-erythrocytic protection against *P. vivax* malaria infection.

Protocol Summary

Schematic overview of the study design
Steps 1, 2 and 3

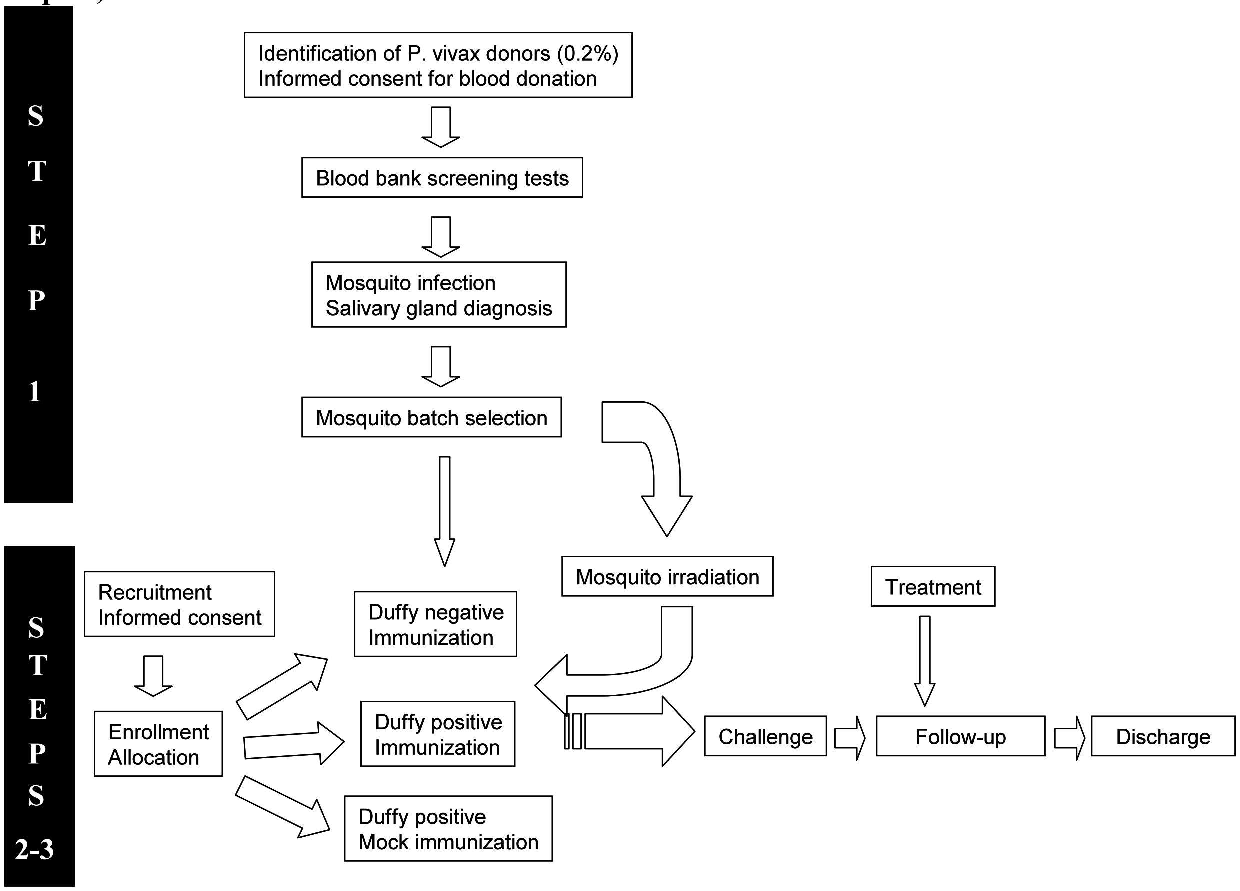

Protocol Summary

Schematic Study Design
Steps 2 and 3

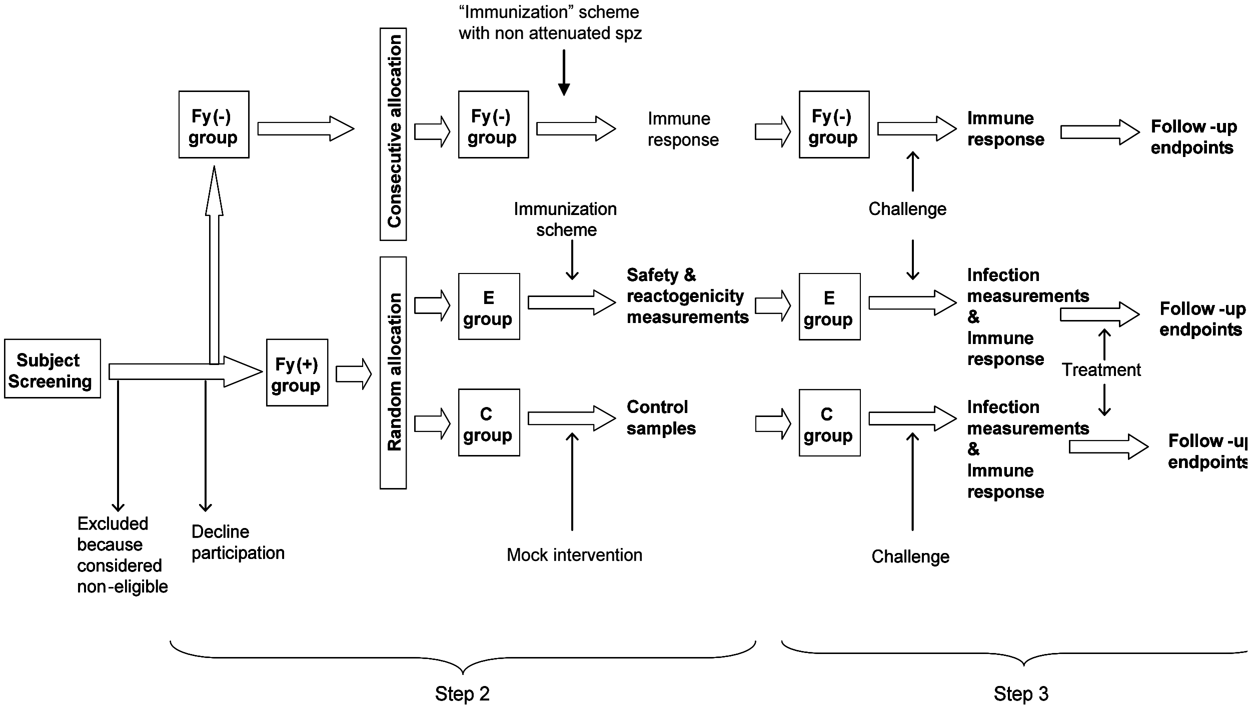

Protocol Summary

Schematic design of the immunization schedule
Step 2

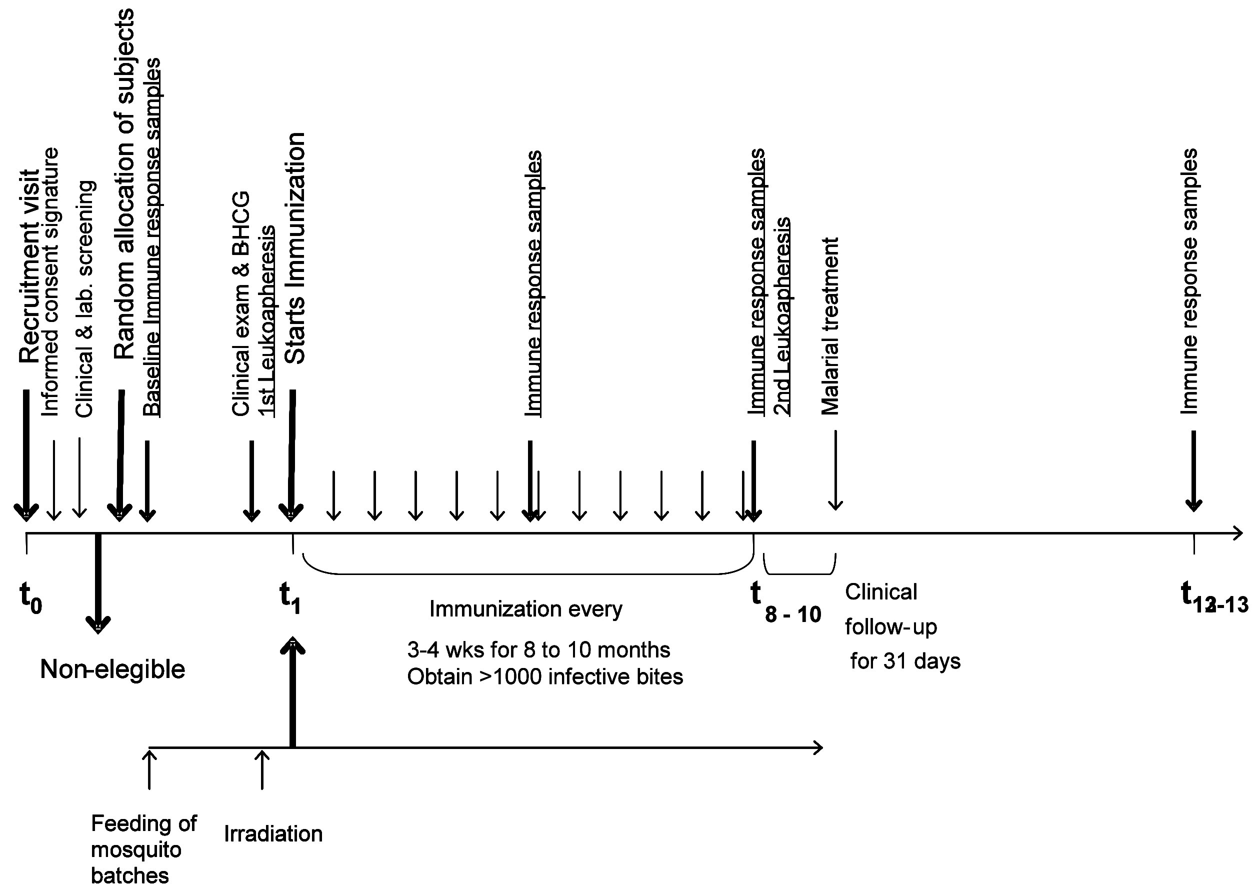

Protocol Summary

Schematic design of the immunization schedule
Step 3

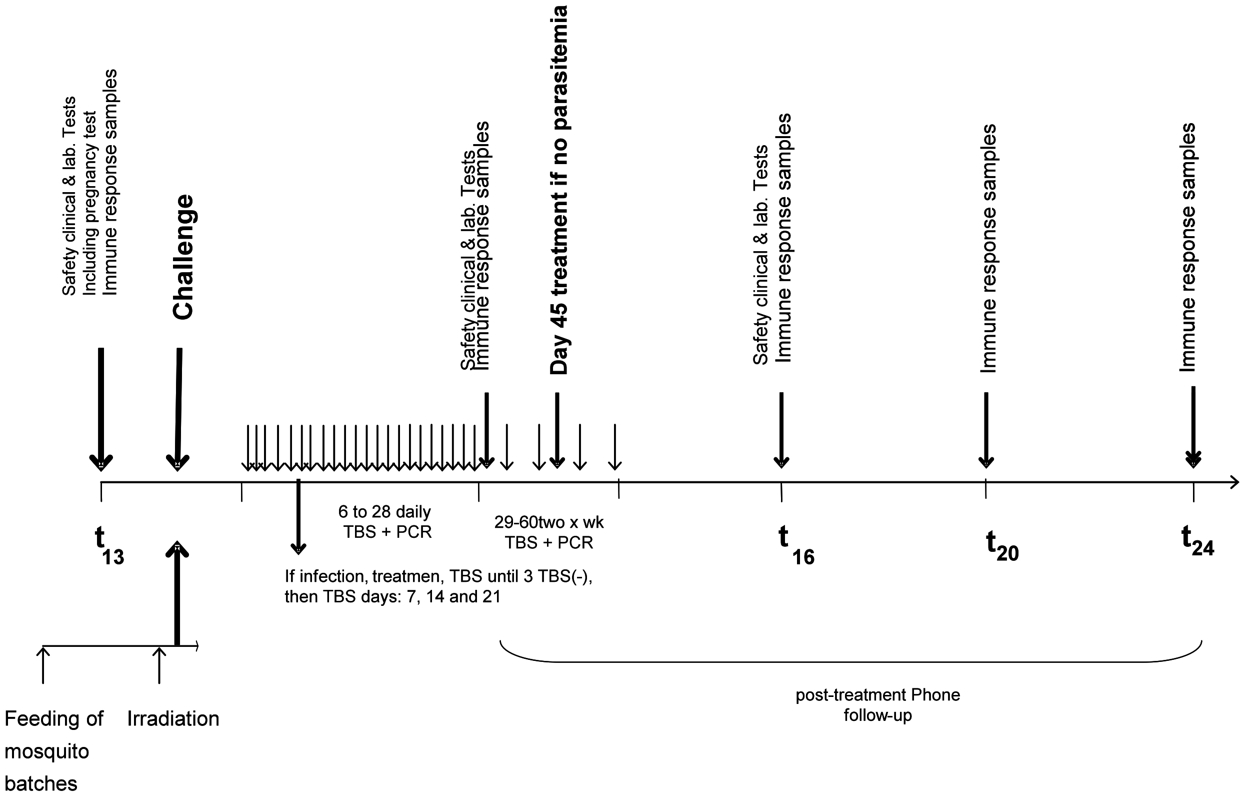

1. Key Roles

Institutions:	**Malaria Vaccine and Drug Development Center**<br>Corregimiento El Hormiguero, Km 6 vía Cali - Puerto Tejada.<br>Cali, Valle del Cauca, Colombia.
Principal Investigator:	**Sócrates Herrera, MD**<br>Malaria Vaccine and Drug Development Center<br>Corregimiento El Hormiguero, Km 6 vía Cali - Puerto Tejada.<br>Cali, Valle del Cauca, Colombia.<br>Phone: (+57) 317 5170552, (+572) 5216228<br>e-mail: sherrera@inmuno.org
Co-Investigator:	**Myriam Arevalo-Herrera, PhD**<br>Malaria Vaccine and Drug Development Center<br>Corregimiento El Hormiguero, Km 6 vía Cali - Puerto Tejada.<br>Cali, Valle del Cauca, Colombia.<br>Phone: (+57) 317 5170557, (+572) 5216228<br>e-mail: marevalo@inmuno.org
Co-Investigator	**Jose Millán Oñate, MD**<br>Centro Médico Imbanaco<br>Carrera 38 A No. 5 A 100<br>Phone: (+572) 6821000<br>Cali, Valle dell Cauca, Colombia
Clinic monitor:	**Ricardo Palacios, MD, PhD**<br>Meridional R&D<br>Rua Fernão Dias, 128 / 34A<br>São Paulo, SP, Brasil<br>CEP 05427-000<br>Phone: (+5511) 939 40670<br>e-mail: rpalacios@meridionalrd.com
Ethics committee:	Comité de Ética, CIV (CECIV)<br>IRB# IRB00007039 - IRB00007040<br>FWA: FWA00016072<br>Phone: (+572) 5185677 Fax: (+572) 554284<br><br>Comité de Ética, Centro Médico Imbanaco (CMI)<br>Carrera 38 A No. 5 A 100 Cali, Colombia.<br>Phone: (+572) 6821000<br>Fax (+572) 5186000

Clinical Laboratory:	ASOCLINIC Inmunologia Ltda.<br>Cra 37 2 Bis 5E-08 Templete Cali, Colombia<br>Phone: (+572) 5574929
Radiotherapy units:	**Centro Médico Imbanaco (CMI)**<br>Carrera 38 A No. 5 100 Cali, Colombia.<br>Phone: (+572) 6821000<br>Fax (+572) 5186000<br><br>Hospital Universitario del Valle<br>Calle 5 No. 36 - 08 San Fernando Cali, Colombia<br>Call Center: (+572) 6206275 - Conmutador: (+572) 6206000<br>Web: http://www.huv.gov.co

2. Introduction

2.1 Background Information and Scientific Rationale

2.1.1 Current significance of the proposed model.

The field of malaria vaccine development has changed since we first submitted this proposal. First of all, a number of the asexual erythrocytic *Plasmodium falciparum* vaccines have failed in field studies, and there is now an increasing emphasis on pre-erythrocytic stage vaccines. Second, the work of Sanaria Inc. (Rockville, MD) to develop an attenuated whole sporozoite *P. falciparum* vaccine (preerythrocytic) that completely prevents infection, has been increasingly embraced by the malaria vaccine development community as feasible and the best, most short term approach to yielding a highly effective malaria vaccine.

In this context, our proposed set of studies to establish that protective immunization with irradiated *P. vivax* sporozoites can be obtained in humans, are critical as the first step towards developing preerythrocytic *P. vivax* vaccines. The development of an attenuated *P. vivax* sporozoite vaccine will require either the development of a continuous culture system to produce *P. vivax* gametocytes, or a method of obtaining, and cryopreserving large number of *P. vivax* gametocytes. Both of these strategies will require significant investment of research funds. To our current knowledge, only one individual has thus so far been fully immunized, and protected against *P. vivax* by immunization with irradiated *P. vivax* sporozoites. Therefore, it is critical that a proof-of-principle can be established for immunization with irradiated *P. vivax* sporozoites, as it has been demonstrated for *P falciparum*.

Thus, we would like the study section to view our proposal as having one overriding primary mission, which is to establish that humans are protected against *P. vivax* infection by immunization with irradiated *P. vivax* sporozoites. The secondary objective is providing serum and cells to understand the mechanisms of protective immunity and the antigenic targets so as to develop an effective subunit vaccines are still important, but of secondary importance as compared to our primary objective of establishing a solid proof of principle of protection as a foundation for development of an attenuated *P. vivax* sporozoites vaccine.

2.1.2 Former studies on malaria irr-spz models.

Although acquisition of immunity to malaria in endemic areas is a slow process and is never sterilizing, there is substantial evidence that vaccines against malaria, in particular, vaccines capable of targeting the pre-erythrocytic stages of malaria are feasible [4, 5]. Among the various stages of malarial infection, sporozoite (spz) invasion and schizogonic development in the liver cell appear to be ideal therapeutic targets. This is because immune responses' killing these parasite forms halts its development during the non-pathogenic liver stages, prior to the initiation of the pathogenic blood stage infection and, therefore preventing all manifestations of infection. Immunization of humans and experimental hosts with radiation-attenuated spz (irr-spz) can induce sterile immunity by killing the pre-erythrocytic stages of the malaria parasite, providing proof-of-concept for this approach. Studies have established that immunization with *P. berghei* and *P. yoelii* irr-spz protects rats and mice against spz challenge [6-11]. Similarly, immunization with *P. cynomolgi*, *P. knowlesi* and *P. vivax* irr-spz partially or totally protects monkeys [12-15] and immunization with *P. falciparum* or *P. vivax* irr-spz protects humans [3, 16-22]. In 1973, Clyde and colleagues at the University of Maryland [17-20] and Rieckmann, Beaudoin and

colleagues at the Naval Medical Research Center (NMRC) [21-23] reported the first studies of human protection using irr-spz.

Mosquitoes with high-grade spz infections were exposed to 15,000 to 20,000 cGy of gamma-irradiation, and then allowed to feed on malaria-naive volunteers. After multiple immunizations over the course of several months, with a total of 200 to over 1000 infective bites, human volunteers were challenged with mosquitoes harboring non-attenuated, infectious spz. Volunteers immunized with >1000 bites from irradiated mosquitoes harboring either *P. falciparum* or *P. vivax* spz were consistently protected against challenge with non-irradiated spz from the same malaria species. Protection appeared to be species-specific, as volunteers immunized with *P. falciparum* were fully susceptible to challenge with *P. vivax* spz but protection was not strain-specific in that immunized volunteers were protected against challenge with heterologous strains of the same species. Additional studies established that this protective immunity was stage-specific, directed against sporozoite and/or liver stages, because immunized animals or volunteers remained susceptible to blood-stages challenge.

While the original studies, conducted in the late 1960s and early 1970s, have been extended during the last fifteen years to confirm the efficacy in humans of the *P. falciparum* model, the *P. vivax* model has not been reproduced since 1975, and prior to then it was only studied in 5 volunteers in two independent studies. Only one of these received >1000 infectious bites, and was protected from the challenge with infectious sporozoites. Given the importance of the irr-spz model and the epidemiological relevance of *P. vivax* that continues to affect millions of people in endemic countries, it is therefore essential to establish again the attenuated *P. vivax* sporozoite vaccination system.

In order to study the *P. vivax* irr-spz model, a standardized method for producing infectious *P. vivax* spz that provides material for immunization and for experimental challenge was needed. In the past, *P. vivax* sporozoite production and challenge were performed routinely when *P. vivax* infection was used as malaria-therapy for patients with neurosyphilis, but when better methods to treat syphilis were developed, this procedure was abandoned. It has been possible to continue the production of *P. falciparum* spz because of the ease to maintaining this species in culture and produce gametocytes *in vitro,* to feed mosquitoes via membrane feeding. However, because of the difficulty of maintaining *P. vivax* in culture, there is currently no option for producing spz outside of endemic areas where infected patients are available, and can serve as donors of gametocyte-infected blood in order to initiate the sporogonic cycle in the mosquito vector and thereby produce infective sporozoites.

2.1.3 Sporozoite challenge system and testing the irr-spz vaccine.

In a 1970 study, Powell *et al* assessed the efficiency of 1 to 5 bites as compared to >10 bites by *P .falciparum*-infected mosquitoes fed with blood from patients, for inducing malaria infection in healthy volunteers. After the establishment of the *P. falciparum* NF54 isolate in continuous culture, a parasite that produces gametocytes *in vitro,* it has been routinely used to infect *A. stephensi* mosquitoes. Rieckmann demonstrated that malaria was transmitted to only half of the volunteers after exposure to 1-2 sporozoite-carrying mosquitoes, whereas Church [30] found that exposure to the bites of 5 infected mosquitoes induced blood infections in all exposed volunteers.

Infectivity however did not correlate with the numbers of spz in the mosquito salivary glands, but a negative correlation was observed between the length of the pre-patent period and the number of bites by infective mosquitoes. A 5 bites challenge regimen is currently used at the NMRC for inducing reliable *P .falciparum* infection, and also by other institutions maintaining the *P. falciparum* sporozoite model, including the Walter Reed Army Institute of Research (WRAIR), Oxford University and the University of Nijmegen.

P. vivax gametocytes, on the other hand, can only be obtained from fresh specimens of parasitemic blood taken from individuals with active infections. If mature gametocytes are present in these samples, spz can usually be generated via artificial-membrane feeds. This process has to be repeated every time spz are needed, and is therefore totally dependent upon the availability of malaria-infected humans. During the last few yeas, in the context of a NIAID sponsored TMRC program, we at MVDC have been setting up the conditions to establish again the *P. vivax* irr-spz model in primates and human in Colombia (South America). First we established a continuous supply of *P. vivax*-infected patients in outpatient clinics in Buenaventura town (endemic area) and in Cali city (non-endemic), and we set up two *A. albimanus* colonies for regular sporozoite production. These facilities have been used to conduct a pilot clinical trial in which 18 malaria naive human volunteers were exposed to decreasing doses of *P. vivax*-infected mosquito bites: 9±1 6±1 and 3±1. Seventeen of the 18 volunteers became infected, with prepatent periods ranging from 9 to 13 days (mean 11 days). There was no correlation between prepatent period and number of infective bites given. All individuals cleared their parasitemia within 24-36 hour of treatment with chloroquine and primaquine.

In order to determine the reproducibility of the minimal spz challenge dose, more recently we conducted a second challenge trial in which, once more, 18 malaria naïve human volunteers were allocated to three groups of six volunteers and each group was exposed to the bite 3±1 mosquitoes infected with parasites from a different donor (a volunteer declined his participation). Eleven volunteers received only two mosquito bites and all 17 volunteers became infected with prepatent periods of 9-16. All volunteers cleared parasitemia rapidly after treatment and none developed serious adverse events related to the mosquito bites, the infection or the treatment (See Preliminary studies, Development of a *P. vivax* spz challenge model for human volunteers). In addition, we have standardized the irradiation of infected mosquitoes and have carried out preclinical studies in which *Aotus* monkeys have been immunized 10, 5, and 2 times by intravenous inoculation of 105 irr-spz obtained by dissection of infected irradiated *A. Albimanus* mosquitoes. Primates immunized five and 10 times developed significant immune antibody and IFN-γ responses to both spz and to the *P. vivax* circumsporozoite (PvCS) protein and displayed dose dependent protection [37-39]. These preliminary studies have demonstrated the feasibility of the process for preparation of radiation-attenuated parasites, as well as the possibility to safely challenge human volunteers.

2.1.4 Studies on the immune response induced by irradiated sporozoite.

Mice represent the most extensively studied model for vaccination with irr-spz. These studies indicate that the induced immunity is complex and multifactorial, with several humoral and cell mediated mechanisms capable of contributing to protection. *First,* passive transfer of Mabs to the repeated central region of the CSP protects mice and monkeys from sporozoite infection,

indicating that anti-spz antibodies play a role in protection. *Second,* the role of CD8+, CD4+ and γδ-T cells in malaria protection has been conclusively documented. Adoptive transfer experiments of both CD8+ T cells clones [44] and CD4+ T cells and γδ T-cell clones have conferred protection to mice [45, 46]. However, protection of CD95/CD95L deficient mice against spz challenge indicated that the perforin and receptor/ligand systems were not involved in the T-cell killing of malaria infected hepatocytes.

On the other hand, CD4+ T-cell restricted epitopes that correlated with protection associated to IFN-γ have been identified in the CSP. *Third,* systemic inoculation of recombinant IFN-γ in both mice and monkeys protects them from spz infection while this protection could be abolished by treatment with anti-IFN-γ antibodies. *Fourth*, IL-12 could also completely protect mice and monkeys against spz infection and protection could also be achieved by *in vivo* administration of N-monomethyl-L-arginine that inhibits nitric oxide synthesis. Together these results may indicate that IL-12 derived from macrophages, dendritic or Kupffer cells induces CD8+ T cells and natural killer cells to produce IFN-γ that then induces infected hepatocytes to produce nitric oxide that kills the intra-hepatic parasite.

Several *P.falciparum* vaccine candidates have been identified using reagents generated through human irr-spz studies, as well as cells and sera of individuals from malaria endemic areas. Two proteins CS and TRAP/SSP2 were identified two decades ago, and several liver stages antigens (LSAs), more recently. Extensive characterization, mainly of the CS protein has been performed. Such analysis suggested that CS & TRAP may be presented to the immune system differently from the LSAs, since mice and human immunized with irrad-spz do not develop recall proliferative responses to the CS, whereas they do respond to LSA-1 stimulation, and in natural conditions humans develop recall responses to both antigens. On the other hand, natural exposure to the parasite produces much shorter lived immune responses than exposure to irr-spz does, which may indicate the persistence of irr-spz induced CD8+ CD45RBlo CD44hi in the liver. Moreover, in humans, naive T cells differentiate to memory T cells by expression of the CD45RO isoform. CD45RO+ T cells expand rapidly after activation by specific antigens and produce large amounts of IL-4, or IFN-γ and IL-2. CD4+D45RO+ T cells producing IL-4 play a role in the protective immunity against *P. falciparum* infection. Among the CD4+D45RO+ T cell subsets, CD27+ is considered a marker of immature memory T cells. The loss of the CD27 marker is irreversible and defined as a marker of mature memory T cells. A significance of CD27+ has been shown in long-term protection against *P. falciparum* infection. It would be of great importance to address the B-cell memory [CD19(+) CD38(-) CD27(+)] to specific antigens such as CS and TRAP derived from spz in the vaccinated volunteers and its association with Toll-like receptor expression (TLRs)

2.1.5 Significance of a reproducible *P. vivax* spz challenge model and advantages for conducting research in an endemic area.

Standardization of the sporozoite challenge model is now expected to greatly accelerate the development and testing of *P. vivax* vaccines since several *P. vivax* antigens are already being tested in preclinical and clinical trials by our group and other groups worldwide. This is particularly true for the circumsporozoite (CS) protein, which is the main surface protein of the sporozoite, and has already been tested in Phase I clinical trials indicating reproducible safety,

tolerability and immunogenicity and was demanding the availability of a proven challenge system. Similarly, the development of *P. vivax* vaccines against asexual blood forms such as the Duffy Binding Protein (DBP), and Merozoite Surface Protein 1 (MSP-1) has literally been arrested due to the absence of a *P. vivax* challenge model. Fortunately during the period this project has been under evaluation, we have been able to develop a safe, well-tolerated and reproducible *P. vivax* sporozoite challenge model that is now available to test these vaccines, and to develop the irr-spz vaccine model. The success achieved so far, is expected to have immediate bearing on the development of *P. vivax* vaccines as several vaccine leading groups worldwide are now preparing to test experimental subunit vaccine.

A critical condition to develop the *P. vivax* sporozoite challenge model has been the proximity to the malaria endemic area and the permanent availability of infected human blood for regular production of *P. vivax* sporozoites in Buenaventura and in Cali (non-endemic city). It also facilitated the establishment of the irr-spz model in non-human primates that has allowed us to conduct preliminary studies on the immune response in *Aotus* monkeys and has permitted to get valuable training on the logistically challenging process of regularly producing irr-spz for serial vaccinations. The successful establishment of the challenge model is encouraging particularly for the reproducibility already achieved with four different *P. vivax* isolates. Therefore, the plan for the irr-spz vaccine model in humans and producing the reagents to characterize the protective immune responses seems highly likely to succeed. Most techniques have already been put in place and together the experience and expertise of our group in Cali and the highly qualified collaborators there are the most favorable conditions for optimal use of the reagents generated in these trial(s). Moreover, the sporozoite challenge system already developed by our group may also allow in near future the study of novel drugs for *P. vivax* prophylaxis as well as the exploration of the potential use of cytokines such as IFN-γ for the elimination of hypnozoites in relapsing patients, which would complement the information provided by the irr-spz vaccination model.

3. Study hypothesis

It is possible to safely protect human volunteers immunized with *P. vivax* irradiated porozoites from *P. vivax* challenge with live sporozoites.

4. Potential risks and benefits

4.1 Potential Risks to volunteers immunized with irradiated spz and challenge

We consider as the most frequent potential risks associated with immunization with *P. vivax* irradiated, non-irradiated sporozoites, and challenge with *P. vivax* viable sporozoites to include:

- Local inflammatory reactions to mosquito bites
- Risk of anaphylactic reaction
- Possible transmission of other infectious agents through mosquito bite
- Drug side effects associated with antimalarial drugs.
- *P. vivax* malarial infection
- Venipuncture risk.

4.2 Known potential benefits to volunteer

There are no direct benefits to volunteers for participating in this study. However, the volunteers could receive an indirect benefit given that they will be screened for multiple infectious diseases as part of the blood bank screening. If they are found to be positive for any infectious diseases other than *P. vivax*, including HIV, they will be referred to their health care providers as described above for counseling and further medical attention.

4.3 Potential risks to infected blood donors

We consider as potential risks to infected blood donors, those associated with blood drawing including:

- Pain and discomfort at the moment of the venipuncture
- Swelling, bruising, and/or infection at the blood drawing site
- Lightheadedness or fainting associated to venipuncture

Venipuncture will be performed using standard sterile technique and sterile disposable supplies. A physician will be present to administer first aids, e.g., treatment of vasovagal (fainting) episodes. A short delay (15-30 min) in receiving the first dose of anti-malarials may be associated with the blood drawn for infected donors; however, this risk should not significantly affect the volunteer's recovery. Although the bleeding procedure is usually very short, all efforts will be made to expedite procedures so that anti-malarials can be started as soon as possible.

4.4 Known Potential Benefits to infected blood donors

A physician will perform a complete physical examination and potential participants will receive counseling and referral if a medical condition other malaria is suspected or diagnosed. These subjects will be referred to their social security service (EPS: Empresa Promotora de Salud). Physical examination is not usually performed at malaria diagnostic posts and therefore, represents a potential benefit for potential volunteers. There are no other foreseen direct benefits for the participants.

There are no other foreseen direct benefits to volunteers for participating in this study. Participants will not have expenses derived of their participation in the study and as requirement from the Ministry of Health there must be no monetary compensation. Treatment with curative doses of antimalarials according to the standard therapeutic protocol recommended by the Ministry of Social Protection of Colombia will be given for free for those infected at the moment of examination. Treatment will consist of primaquine 15 mg/day for 14 days plus chloroquine 600 mg/day and 450 mg/day for 3 days. Light refreshments will be provided after the blood donation.

5. Objectives

5.1 Primary

To assess protective efficacy and safety of *P. vivax* irradiated sporozoite vaccination.

5.2 Secondary

- To determine the immune response elicited by the P. vivax challenge in human Fy(+) volunteers previously immunized with irr-spz, as compared with non-immunized human volunteers.

- To determine the immune response elicited by irr-spz immunization scheme in human Fy(+) volunteers; comparing it with the same immunization scheme but with non irr-spz in Fy(-) participants, and also with controls exposed to the same mosquito bite scheme as that of immunization but without parasite infection.

- To study new antigens potentially useful for pre-erythrocytic protection against *P. vivax* malaria infection

6. Study Design

This is a randomized, single center, single blinded study, with three parallel arms, that will be developed in 2 consecutive steps, involving healthy adult outpatient participants. It will also include a preliminary step with malarial patients to obtain sufficient amounts of infected mosquitoes for the trial.

Batches of 10,000 *Anopheles albimanus* mosquitoes will be infected by artificial membrane feeding from *P. vivax* infected donor. Mosquitoes will be checked for oocyst production on day 7 and sporozoite production by day 14. Once mosquitoes are positive for sporozoites in their salivary glands they will be irradiated at 15000 cGy and kept in boxes until use. *P. vivax* infected blood will be tested for blood bank laboratory test.

After informed consent, 14 Fy(+) human volunteers in the experimental group will be immunized with 500 *P. vivax* irradiated sporozoites bites each. Seven Fy(+) volunteers in the control group will be exposed to non-infected mosquito bites. Seven Fy(-) volunteers will be exposed to infective mosquito bites. For the challenge both the immunized and control group will be exposed to 3 ± 1 infective mosquito bites. Volunteers will be closely monitored post infection and will be treated as soon as infection becomes patent in peripheral blood. In addition, volunteers will be bled before immunization and challenge for both humoral and cellular immune evaluation.

6.1 Steps of the study

The study will be divided in three successive steps:

6.1.1 Step 1, Parasite infected blood donation

In this step we will obtain infected malaria patients that will donor parasitized blood for mosquitoes infection. Volunteers will be recruited from among the patients who present with active *P. vivax* infection to the laboratory for malaria diagnosis at the MVDC/Asoclinic in Cali (non-endemic region) and to the MVDC/Instituto de Inmunología del Valle in Buenaventura (endemic region) as determined by thick smear. After free and willing written informed consent, a total of 35 ml of whole blood will be obtained by venipuncture from each donor, and will be distributed into 5 ml for infectious diseases screening, and a 30 ml fraction that will be used to feed batches of adult colonized *Anopheles albimanus* mosquitoes.

6.1.2 Step 2, Sporozoite immunization

Twenty one Fy(+) volunteers will be randomized into two groups, allocation 2:1. One, named "experimental group" will receive immunization through the bites of infected and irradiated (15K – 20K cGy) mosquitoes. The other, named "control group" will be exposed to the bites non-infected non-irradiated mosquitoes. Details about recruitment, and study procedures are described below. A third group with 7 Fy(-) volunteers will be sequentially allocated to receive infective non-irradiated mosquito bites.

6.1.3 Step 3, Sporozoite challenge

Volunteers in the experimental and control groups from step 2 will be challenged with viable sporozoites by infective mosquitoe bites. The objective of this step is to determine the protection efficacy against *P. vivax* infection elicited by immunization with irradiated sporozoites. We expect that at least 10 volunteers in the experimental group and 5 in the control group will successfully finish step 2 and continue to participate in step 3. They will be challenged with the bites of 3 ±1 *P. vivax* viable sporozoites infected mosquitoes. Volunteers will be closely monitored post-infection, and will be treated as soon as infection becomes patent in the peripheral blood. The comparison of two groups will help us to assess the induction of protective immunity following immunization.

7. Study population

7.1 Selection and recruitment procedures of participants

7.1.1 Step 1. Parasite infected blood donation.

7.1.1.1 Subject selection for step 1

This step will recruit outpatient participants with *P. vivax* disease, for mosquito infection. They will be recruited from the patients presenting to the MVDC or Asoclinic in Cali, and to the Instituto de Inmunología del Valle in Buenaventura. These institutions are certified by the Colombian government for performing malaria diagnosis. These patients are examined by a physician on site and a blood sample is taken for TBS and thin smears. Volunteers who have been identified to have high levels of *P. vivax* gametocytemia ($\geq$0.1%), with no evidence by blood smear of other *Plasmodium* species, will be informed about this study and, if interested, will be asked to provide a free and willing informed consent for screening, participation and HIV test. If the volunteer is a minor (15 – 17 years old), the study will be explained to both, the volunteer and the minor's parents.

7.1.1.2 Inclusion and exclusion criteria for participants in step 1

Subjects must meet all of the following inclusion criteria in order to be eligible for participation in this study:

- Fifteen to 60 years old, man or non-pregnant women.
- To have a positive diagnosis of *P. vivax* malaria by thin and thick blood smear (TBS).
- Not to have malarial coinfections, such as with *P. falciparum* o *P. malariae*.
- To have a parasitemia > 0.1% in TBS by microscopic exam and minimum 0.1% of gametocitemia.

- Hemoglobin levels ≥ 9 g/dL, at the moment of diagnosis (by HemoCue).
- He (she) should have the capacity to sign an ICF freely and voluntarily. For subjects aged 15 to 17 years, ICF will be signed by the attending parent or legal tutor and a witness, whereas assent will be obtained from the participant.

Subjects must not meet any of the following exclusion criteria in order to be eligible for participation in this study:

- To have chronic or acute diseases, other than *P. vivax* malaria.
- To have history of illness or clinical conditions, which could substantially increase the risk of complications or adverse outcomes associated with their participation in this study, as judged by the trial's clinicians.
- To have hemoglobin less than 9 g/dL, at the moment of recruitment
- He (she) has received anti-malarial treatment before carrying out the diagnosis.

7.1.2 Step 2 and 3. Sporozoite immunization and challenge
7.1.2.1 Subject selection for steps 2 and 3.
Volunteers will be recruited in Cali, a non-malaria-endemic city, using posters, public conferences and individual meetings explaining the aims of the study. All the information used for recruitment has to be previously approved by the IRB. Once the participant manifests his intention to participate, inclusion and exclusion criteria will be evaluated and a blood sample will be drawn for screening tests. If the candidate is considered to be eligible, all procedures, risks, benefits, and commitments involved in the research process have to be clearly understood by the participant. If this is the case informed consent has to be signed by him, and witnesses. If the participant is found to be Fy(+), they will be randomly assigned to one of the experimental groups. If the participant is found to be Fy(-), they will be entered consecutively in a separate group.

7.1.2.2 Inclusion and exclusion criteria for steps 2 and 3
Subjects must meet all of the following inclusion criteria in order to be eligible for participation in this study:

- Healthy 18 to 45 years old man or non-pregnancy women.
- To have the capacity to sign an informed consent freely and voluntarily.
- Have an acceptable understanding of the clinical trial through the approval of a questionnaire about the information given in the consent process.
- Agree to use an effective anti-conceptive throughout the whole duration of the study.
- Not to have chronic diseases or acute diseases. These conditions will be determined by clinical history, physical exam and laboratory tests.
- To be willing not to travel to endemic areas during their participation in the trial.
- To have telephone through which they can be contacted at any time.
- To be willing to participated during both steps of the clinical trial.

Subjects must not meet any of the following exclusion criteria in order to be eligible for participation in this study:

- People with less than 18 old year or more than 45 old years.
- Pregnant and breastfeeding women will be excluded. Pregnancy will be determined both by interview, and by β-subunit chorionic gonadotrophin (β-hCG) urine test.
- History of moderate or severe insect, or food allergies.
- G-6PD deficiency or any hemoglobin genetic defect (for example; sickle cell disease).
- Previous malaria infection demonstrated by think smear, or PCR, or specific antibodies.
- If the subject has previously participated in a malarial vaccine trial or has received any type of malarial vaccine.
- Clinical record of allergies to drugs or insect bites.
- Symptoms, signs, or data from laboratory test that suggests to the physician any systemic disorder like renal, hepatic, cardiovascular, pulmonary, psychiatric disorders or other illnesses that could interfere with results of clinical trial or could result in any complication.
- If volunteer have antibodies against hepatitis C, VIH, or hepatitis B superficial antigen and/or hepatitis B core antibodies.
- Abnormalities in hematology, urine analyses or blood chemistry that may impair interpretation of safety tests in the follow up throughout the study.
- History of an auto-immune or immune-mediated diseases such as; Asthma, lupus, rheumatoid arthritis, Graves's disease, Hashimoto tyroiditis, and others.
- History of splenectomy.
- Volunteers with medical treatments known to alter the immune within the three months prior to recruitment, such as; cortico-steroids, chemotherapeutic agents, fludarabine, cyclosporine, tacrolimus, mycophenolate mofetil, rapamycine, ATG, alemtuzumab.
- Alcoholism or drug abuse that interfere with social relationships of individual.

8. Enrollment/randomization/masking procedures

8.1 Step1 Enrollment Procedures

Volunteers will be recruited from the *P. vivax* infected patients presenting to the MVDC or Asoclinic in Cali, and to the Instituto de Inmunología del Valle (Pacific Branch) in Buenaventura, as described before. Enrollment will be in a sequential fashion as the cases are diagnosed. No masking or randomization procedures will be applied.

8.2 Step 2 and 3 Enrollment, Allocation and Masking Procedures

Those individuals that assist to the recruitment visit and manifest their interest in participating, will receive a detailed description of the study procedures, risk and benefits. Afterwards , medical history will be obtained by the trial's physicians. If at this point the participants express their willingness to participate in the study and their medical history is uneventful, they will be asked to sign the ICF for both, participation in the study and HIV diagnosis. For female volunteers, a pregnancy urine test (qualitative B-hCG) will be taken and they will be excluded from the study should the test result positive. In case that the result is negative a sample of blood smear and urine will be taken. Blood chemistry, urine deep stick analysis, complete blood count, infectious disease screening and determination of antibodies against blood forms of *P. vivax*, and Fy antigen genotypification will also be performed to all participants. Clinical test will be analyzed by medical staff.

After having the results of this screening, and the subject is finally considered as eligible, Fy(-) individuals will be assigned consecutively as enrolled to be infected by mosquitoes. Fy(+) individuals will be randomly allocated into two parallel groups, one group that we will name as “Experimental or E” and the other as “Control or C”. Duffy antigen status and group allocation will be held from the volunteers but will be known by all the trial’s personnel.
Considering that sample size for this study is relatively small, we considered “minimization” as a better method for allocation than a restricted or an unrestricted randomization. Age groups (18-20, 21-45), and gender, will be used as factors to minimize imbalances among groups. Taver’s method of minimization will be applied. No masking procedure will be used. Allocation of the first subject will be made using random number list, from 0 to 9. Pair numbers will be for one group and impair to the other. Once the first participant has been assigned, the next participant will be assigned using marginal discrepancies or if the groups are balanced simple randomization, as described before, will be used. The allocation of the individuals will be made by a person of the research team, not directly in charge of participant recruitment, intervention of follow-up. This allocation will be centrally and telephone based..
If the participant quits the trial before completing at least three quarters of the immunization scheme, a new volunteer will be included. If the participant quits the immunization scheme after completing at least three quarters of the immunization scheme and accepts that blood samples are taken for immunological studies, they will not be replaced for step 3.

9. Study Procedures/Evaluation

9.1 Step 1. Parasite infected blood donation

Volunteer selection: Volunteers with malaria-related symptoms will be passively recruited in endemic areas (Instituto de Inmunología del Valle, Buenaventura). This site is certified by the Colombian government for performing malaria diagnosis. These patients are examined by a physician on site and a blood sample is taken for TBS and thin smears. Individuals who have been identified to have high levels of *P. vivax* gametocytemia (≥ 0.1%), with no evidence by blood smear of other *Plasmodium* species, will be informed about this study and, if interested, will be asked to provide a free and willing informed consent for screening and participation.

Informed Consent: Individuals who present to Buenaventura with positive blood smears for *P. vivax* will be informed about the study objectives, procedures, risks and benefits. Candidate volunteers will be requested to pass a comprehension test about the study and all items that may not be properly understood will be explained again and reassessed by the screener. All consent and test procedures will be documented in the participant’s chart. An additional informed consent form for HIV testing will be signed. All volunteers will receive a copy of the signed informed consent forms to keep.

Identification: Volunteers will be assigned an identification code consisting of five characters: First character is going to be letter A which is going to be used only for volunteers from step 1, second and third characters are going to be the initial letter of the volunteers name and family name respectively, followed by the number of enrolment. For example a volunteer named Mario

Perdomo, who was the tenth person to be screened, would be assigned the identification code of AMP10.

Blood donation: Once the volunteer has been identified, a medical history and physical exam will be completed by one of the physicians and data will be recorded in the CRF´s. If, after the medical history and exam, the volunteer does not meet enrollment criteria, he/she will be provided with anti-malarial therapy, instructed to return at 10 days as per routine, but will not be enrolled for blood donation. If the volunteer meets the criteria for enrollment, he/she will be asked to participate, and a total of 35 ml of whole blood will be drawn and fractionated into a 5 ml fraction to be used for infectious diseases screening at the blood bank (FCVL) and 30 ml in 3 heparin tubes of 10 ml each, that will be used for MFA at the MVDC insectary.

Volunteer treatment and follow-up: Immediately after blood donation, volunteers will be provided with the anti-malarial treatment recommended by the Colombian Health Ministry. Patients be given 600 mg of chloroquine (four 150 mg tablets) on the first day, 450mg (three tablets) on the second and third day, plus primaquine 30 mg (two 15mg tablets) per day for 14 days. Volunteers will be asked to return to the Buenaventura diagnosis site approximately 1 week later to receive the results of the blood bank screening. In the event of a positive result for any of the blood screening tests done, including HIV, the volunteer will be counseled about their diagnosis and advised to seek care with their health care providers in Cali or Buenaventura, with a copy of their results. They will also be asked to return 2 weeks later (Day 10) to have a TBS done to assure malarial cure. If the blood smear is positive at Day 10, he/she will be treated with Pyrimethamine/Sulfadoxine (PS) combination (Fansidar^{R}) as a single dose of 3 tablets (each tablet contains 25 mg of pyrimethamine plus 500 mg of sulfadoxine). If the patient has a contraindication to fansidar (ie sulfa allergy), he/she will receive amodiaquine and will then be asked to return 1 week later to obtain a smear to confirm cure.

Blood screening:

- *Plasmodium PCR:* A sample of 500 µl of donated blood will be used to set up a nested PCR to screen for *P. vivax*, *P. falciparum* and *malariae*.
- *Blood bank screening:* 15 ml of donated blood will be used to test for HIV antibody (MEIA-Abbot Microparticle immunoassay), HTVL-1 and -2 antibodies (MicroELISA Abbot Murex), anti-hepatitis B surface and core antibodies (MEI-Abbot), anti-hepatitis C antibodies by rapid test (MEIA-Abbot), Rapid diagnostic test for Chagas disease (MicroELISA Bioschile) and syphilis (RPR). Although it appears to be theoretically possible that mosquitoes could shed Hepatitis B virus within 72 hours after a blood meal (Blow 2002), we could find no evidence that any of these diseases are actually transmitted by *Anopheles* mosquitoes. In addition in this study there will be an incubation period of two weeks, which will make transmission of any of these infections unlikely.

- *Additional screening:* In addition to routine blood bank screening we considered all other human pathogens, which could be potentially transmitted inadvertently by *Anopheles* during the challenge. We considered other tropical diseases, which have been found (presently or in the past) in Colombia, which are known to or could possibly be transmitted by *Anopheles* mosquitoes.
- Filaria: Discussions with leading experts in the field, including Dr. Augusto Corredor formerly director of the Parasitology Unit of the National Institute of Health of Colombia and Dr. David Botero (parasitologist) former professor at Universidad de Antioquia (Medellin, Colombia) confirmed that there is no current evidence of transmission of any filarial species in Colombia and thus no need to screen for these parasites. This concept was confirmed by Dr. Dwayne Grubman, Chief of the CDC Section on Filarial Diseases.
- Other parasites: *Leishmania spp* are endemic to the Colombian Pacific Coast but are not transmitted by *Anopheles* mosquitoes.
- Viruses: Dr Jorge Boshell (virologist), current director of the National Institute of Health of Colombia and Dr. Robert Tesch (Galveston, Texas) were consulted regarding the potential need for screening donated blood for viruses other than those included in the Blood Bank screening, and both considered that this would not be necessary.

Confirmatory tests:

a. In case any of the rapid HIV tests result positive, a confirmatory test (Western Blot) will be carried out.
b. If RPR result positive (at any dilution), FTA –ABS will be performed.
c. If the HBsAg is positive, antibodies against HBcAg will be analyzed.

9.1.2 Preparation of sporozoites

9.1.2.1 Mosquito Infection

Female *Anopheles albimanus* mosquitoes will be prepared at the insectary of Caucaseco scientific consortium or Malaria Vaccine and Drug Development Center (MVDC) in Cali, under GLP-like conditions. The parasite (blood samples) collected in step A will be confirmed for the presence of *P. vivax* malaria parasites both by microscopic examination of thick blood smears (TBS) stained with Field stain and by PCR. While the latter allowed the precise identification of the parasite species and had greater sensitivity (8-10 parasites/ ml), the TBS allowed the quantification of the total parasitemia and gametocytemia. The parasitemia will be determined by TBS after reading of 300 microscopic fields, by 3 independent, well trained readers. Samples must have a gametocytemia above 0.1% to be included.

The blood samples will be used to infect an equal number of mosquito batches containing 10,000 mosquitoes per batch. Mosquitoes were 3-4 days old females and will be maintained fasting overnight. Before mosquito feeding, blood samples will be centrifuged at 3000 rpm for 5 min at room temperature and autologous plasma will be removed. The blood will be washed with RPMI1640 medium and reconstituted to 50% hematocrit with equal volumes of pooled AB non-immune human serum obtained from the Red Cross blood bank. The complement of the AB

serum will be inactivated by heating it at 56 ºC during 30 min. After blood washing mosquitoes will be feeding using a water-jacketed membrane apparatus at 37ºC as previously described (Hurtado et al, 1997). Cages will be labeled with a feeding code and date of infection. The day after feeding, those females that did not take blood will retired from the cage and the feed mosquitoes will maintained under strict bio-safety standards at temperature and humidity conditions described in the Investigator's Brochure.

Samples of feeding mosquitoes will be dissected on days 7 to 8 after feeding to determine the presence of oocysts in their midguts and on days 14 to 15 to assess the sporozoite load. 40 mosquitoes were dissected and midguts were stained with 2% mercurochrome. A sample of the surviving mosquitoes will be dissected to examine microscopically the presence of oocysts in midgut preparations. Midguts will be stained with 2% mercurochrome and will be examined as described by Eyles. The remaining mosquitoes will keep inside the bio-safety room for infected mosquitoes and maintained for another seven-day period. The dissected salivary glands (6 lobes) will be mounted on a glass cover slip in a drop of PBS, and will disrupt by applying pressure. Salivary glands will examine microscopically at 400X magnification to assess sporozoite (spz) density per paired gland. Each preparation will be graded as 1+ (1-10 spz), 2+ (11-100 spz), 3+ (101-1000 spz) and 4+ (> 1001 spz) (Chulay et al 1986). From the positive batches from volunteer with infectious disease will be discharge and other batches will be used to immunizations. Each mosquito sample for oocyst infection will be calculated according to the equation Nx79/N+79, where N is the number of mosquitoes alive on the day of mosquito dissection. Results will be expressed as the percentage of mosquitoes infected and the arithmetic mean number of oocysts per gut of dissected mosquitoes. To determine the sporozoite presence in the mosquito salivary glands, 38 mosquitoes from each batch will be dissected and microscopically examined on day 14.

9.1.3 Criteria to excluded batches to volunteers' immunization

- Batches infected with blood from exposed volunteer with mixed malaria, HIV, HTVL-1 - 2, Hepatitis B, C, Chagas and Syphilis
- Batches with percentage of sporozoites infected mosquitoes less than 50%.

9.1.4 Irradiation

Batches of infected mosquitoes will be irradiated with 15k to 20K cGys using a linear particle accelerator of 6 meV at the Radiotherapy Unit of the Centro Medico Imbanaco or Hospital Universitario del Valle in Cali.

9.2 Study Procedures for steps 2 and 3

9.2.1 Step 2. Irradiated sporozoite immunization

9.2.1.1 Recruitment

Informed consent: Interested individuals will be informed of the study objectives, procedures, risks and benefits. A comprehension test will be administered to volunteers who wish to be screened for the study to ensure their free and willing informed consent. All items that are not properly understood via the test will be properly explained and reassessed by the screener. All consent and test procedures will be documented in the participant's chart. An additional

informed consent form for HIV testing will be signed. All volunteers will receive a copy of the signed informed consent forms to keep.

Identification: Volunteers will be assigned an identification code consisting of five characters: First character is going to be letter B corresponding to volunteers of the step 2; second and third characters are will be the initial letters of the volunteers first name and family name respectively, followed by the number of enrolment. For example a volunteer named Mario Perdomo, who was the third person to be screened, would be assigned the identification code of BMP03.

Screening: The screening procedures (a medical history, performance of a physical exam, and blood draw) will be performed after volunteers sign the consent form. In the case that a volunteer has been screened and the immunization has not been initiated within 6 weeks, all screening tests will be repeated. At the screening visit, relevant medical history and concomitant therapy will be documented by one of the clinical investigators. A complete physical examination, which includes HEENT, cardiovascular, pulmonary, neurological, gastrointestinal, and dermatological systems, will be performed, and 25 ml of blood will be collected for screening labs. Women who are considered suitable for the study will be geared to avoid pregnancy during the study by using any effective method of contraception. The method of contraception will be chosen in consultation with the clinical trial's physicians.

Screening laboratories: Screening test for challenge volunteers will be performed at ASOCLINIC laboratory, within the 6 weeks before immunization:

Hematological tests: CBC, reticulocyte count, G-6-PD determination, Duffy phenotype, hemoglobin electrophoresis and VES.

Renal function tests: Urinalysis, creatinine, and BUN.

Infectious diseases other than *P. vivax*: HIV, HBsAg, Anticore Hepatitis B, HCV, HTLV-1 and -2, RPR for syphilis and Chagas.

Confirmatory tests: In case any of the rapid HIV test result positive, a confirmatory test (Western Blot) will be realized. If RPR result positive (at any dilution), FTA –ABS will be realized. If the HBsAg is positive, antibodies against HepB core will be analyzed.

Hepatic function tests: ALT, AST, Total bilirrubin, conjugated bilirrubin, non-conjungated bilirubin, LDH, alkaline phosphatase, PT and PTT.

Pregnancy test: Determination of β-HCG in serum.

Others: Glucose, EKG and ANAS.

Non-clinical laboratories

Blood smears: MVDC/Asoclinic have been designated by the State Health Department (Secretaria de Salud Departamental del Valle) as a national diagnostic center for identification of malarial infection. Each smear will be read independently by two microscopists. 200 oil immersion (x 1,000) fields will be examined before reporting that no parasites are found.

- Thick smear (done at screening and for follow-up post challenge or post treatment): Approximately 2 drops of blood will be collected by finger-prick and Field's staining method will be employed as per direction of SOP CD-POE-001-03 (Asoclinic) and recommended by the Ministry of Health of Colombia.
- Thin smears (done at time of screening only): One drop of blood will be collected and SOP CD-POE-001-03 of Asoclinic will be used.

Real time PCR for the diagnosis of malaria: DNA will be extracted from peripheral blood and used for *Plasmodium* diagnosis by RT-PCR. This technique is recognized for its high analytical sensitivity level, allowing to detect 1 parasite/µl. Primers Plasmo 1 and Plasmo 2 and the specific Taqman probes for *P. vivax* y *P. falciparum* will be used for this analysis. For each species and testing, positive and negative controls will be used. The standard curve will be designed for both species in order to establish and quantify the number of copies per sample.

***P. vivax* IFAT:** Antibody titers against *P. vivax* will be assessed by indirect immunofluoresence assay (IFAT) using air-dried *P. vivax* (sporozoites and throphozoite) containing slides. A goat anti-human IgG-FITC conjugated will be used as secondary antibody.

Blood unit donation schedule to obtain PBMCs: This procedure will be performed 30 days before first immunization at the CMI, around the 4 to 6 immunization session, and then around 3 to 5 weeks after the last immunization. Blood unit donation (150-200 ml) will be done using the standard procedures in a blood bank, using a peripheral blood access. A total of $2x10^9$ PBMCs from each donor will be required for evaluation of IFN-γ responses to 2 known and 10 novel *P. vivax* proteins as well for immune response studies. PBMCs will be purified from the blood unit samples and will be cryopreserved in a LN2 freezer at MVDC until use.

9.2.1.3 Pre-immunization

On the day prior to immunization or challenge females will come to the MVDC to have a serum pregnancy test performed and she will be excluded if the result is positive. In such a case the volunteer(s) should be replaced with alternative volunteer(s) before the fourth immunization.

A new physical examination will be performed immediately before the initiation of the immunization phase. This will include ENT system, cardiovascular, pulmonary, neurological, gastrointestinal, urogenital, and dermatology systems. Weight and vital signs (Blood pressure, cardiac frequency, and axillary's temperature) will be measured again at this time and during the rest of the study.

9.2.1.4 Immunization

Volunteers will be assigned to one of three groups:

- Duffy negative group (Fy-): Fy- volunteers will undergo immunization with non-attenuated sporozoites through bites of *P. vivax* infected mosquitoes.

- Duffy positive group (Fy+): Fy+ Volunteers will be randomly allocated in an E group and C group. E group will undergo immunization with radiation-attenuated sporozoites. They will be immunized with bites of *P. vivax* infected mosquitoes irradiated with 15-20Krads of gamma radiation. The aim with the proposed immunization scheme is to reach at least 500 bites in 5 sessions. Each session will be about 4 weeks apart, but with some flexibility allowed (±1 week) due to the difficulties of this intervention.

- C group will be exposed to the bites of non-infected mosquitoes (mock immunized) in parallel to the exposures of the E group. These volunteers will provide samples that can serve as negative controls for the assays performed on immunized volunteers.

All three groups will proceed to step 3 which is an infectious challenge with non-irradiated infective mosquitoes. This step is designed to evaluate the primary endpoint of this research, which is to determine percentage of protection after challenge with infective mosquitoes.

9.2.1.5 Sporozoite Challenge
Sporozoite challenge will be performed under carefully controlled protocols and conditions. The procedure will be carried out in a safe room prepared for challenge inside the Entomology Unit. The "feeding cages" will be placed on the volunteers' forearms for 10 minutes with the aim of achieving the bite of 100 infective mosquitoes. The cages will have a biting window covered by a net; this side of the cage will be in contact with the skin surface.

After feeding, volunteers will remain in the insectary while trained entomologists dissect the mosquito salivary glands to confirm the presence of a blood meal (whether the mosquito fed), the number of infected mosquitoes in the cage, and the mosquito salivary gland score (sporozoite load). If biting rates (as ascertained by presence or absence of a blood meal) and infectivity rates (as ascertained by dissection and salivary gland) are determined to be less than 100, additional mosquitoes will be allowed to feed on the volunteer until a total of 100 infected mosquitoes with sporozoites in their salivary glands have fed.

9.2.1.6 Post vaccination Follow up

- Immediate Follow up: Volunteers will be under medical observation during one hour after each immunization, in order to detect any adverse reaction in the mosquitoes bite. After one-hour observation period, a topical ointment with Hydrocortisone 1% will be applied over the affected skin. Eight hours after the immunization, all the volunteers will receive a phone call to check the volunteer's condition. Any Adverse Event (EA) will be reported in a CRF.

- Post immunization Follow up: Further follow-ups will be done in person on the days 1 and 2 after each immunization. Following evaluations will be done each 15 days before

the next immunization. These follow ups will include a new clinic evaluation and AE recording.

- All the required information will be available to the volunteers to contact the members of the research group at any time (including cell phone number) also they will be encouraged to make questions in case of doubt. During this period, volunteers will be advised not to leave Cali.

9.2.2. Malarial treatment after immunization

One week after the last immunization, all participants included in Fy(+) Experimental group will receive treatment for *Plasmodium vivax* infection. Treatment will be done according to the Colombian Ministry of Health guidelines. This treatment consists of chloroquine (a total of 1500 mg chloroquine base orally in divided doses: 600 mg initially, followed by 450 mg given 24, and 48 hours after the first dose and primaquine for fourteen days (30 mg/day). All antimalarials will be administered with food, as they can all cause stomachache, nausea and vomiting if given on an empty stomach. Although standard dose of primaquine is 15 mg/day, sometimes it is not enough to prevent hypnozoite establishment, this is why volunteers will be treated double doses of primaquine. Participants included in Fy(+) control group (Mock infection) will not be exposed to this treatment, as well as participants of the Fy(-) group.

9.2.3 Last follow-up before step 3

After last immunization dose and the initiation of step 3 "*P. vivax* challenge", a window of time will be allowed of 4 to 6 weeks. During this window participants will be instructed not to leave the city. They will be contacted every two weeks by phone to evaluate their health status, as well as to avoid as much as possible desertions of the study. Before challenge, blood samples will be taken for immunological analyses.

9.3 Study Procedures for step 3

9.3.1 Step 3. Challenge to infected mosquitoes

Challenge will be performed under carefully controlled protocols and conditions already standardized in preliminary trials. The procedure will be carried out in a safe room prepared for challenge inside the Entomology Unit. "Feeding cages" containing three (3) infected mosquitoes will be placed on the volunteers' forearms during 10 minutes. The cages will have a biting window covered by a net. That side of the cage will be pressed to the skin surface. After mosquito feeding, volunteers will remain in the insectaria while trained entomologists dissect the mosquitoes and confirm the presence of a blood meal (whether the mosquito fed), confirm the infected mosquito dose (3±1) in the cage, and records the mosquito salivary gland score (spz load). If biting rates (as ascertained by presence or absence of a blood meal) and infectivity rates (as ascertained by dissection of salivary gland) are determined to be less than 100%, additional mosquitoes will be allowed to feed on the volunteer until a minimum of two (2) mosquitoes carrying *P. vivax* spz in their salivary glands have fed.

9.3.2 Post-challenge evaluation

Volunteers will be followed by phone eight hours after the challenge and the first five days. From Day 6 after malaria challenge until Day 28, volunteers will be evaluated daily at MVDC by

a study physician. The diagnosis will be carried out by TBS taken by finger-prick. If fever develops (axillary temperature > 38ºC), blood films will be obtained twice daily. In the event that a volunteer presents with symptoms compatible with malaria but his/her blood smear is repeatedly negative (three times), a *Plasmodium* diagnostic real time PCR [98] will be done as an immediate way of clarifying the diagnosis. However, the gold standard for treatment will be thick smear. Whole blood will also be collected at the time of each smear to perform quantitative parasitemia analysis by real time PCR for comparative purposes at the end of the trial. If any of the volunteers that develop the infection needs to be hospitalized, the care will be provided in a high-level complexity hospital in Cali. If after 28 days, volunteers are negative, they will continue their parasitological follow-up twice weekly until day 60. During this period volunteers will be followed up by phone every other day. It is expected that at least part of the volunteers develop sterile immunity, however, some may be only partially protected and develop longer prepatent periods. Not protected will be considered as those individuals that become infected and present similar prepatent periods to those of group C.

9.3.3 Malarial treatment

Once malaria infection has been demonstrated, volunteers will be treated with the standard antimalarial treatment approved by the Colombian Ministry of Health (MOH). The treatment for *P. vivax* consists of chloroquine (a total of 1500 mg chloroquine base orally in divided doses: 600 mg initially, followed by 450 mg given 24, and 48 hours later) and primaquine for fourteen days (30 mg/day, two pills/day) administered with food. All antimalarials will be administered with food, as they can all cause stomachache, nausea and vomiting if given on an empty stomach. Although standard dose of primaquine is 15 mg/day, sometimes it is not enough to prevent hypnozoite establishment, this is the reason to treat volunteers with a 30mg/day doses of Primaquine. Volunteers without parasitemia at day 60 will be treated with the same antimalarials scheme, since that day.

9.3.4 Follow up after initiation of antimalarial treatment

Blood smears will be taken every day after initiation of treatment with chloroquine until three consecutive TBS are negative. Follow-up TBS will be done on day 7, day 14 and day 21 after initiation of treatment to ensure cure. In case that any volunteer develops fever or any symptom compatible with malaria a TBS will be done. This therapeutic regime has proven in the two recent challenge trials, to be efficacious to fully control the infection within 1-2 days. On day 45 after malaria treatment is started, volunteers will be evaluated at MVDC by a study physician, and 10mL of blood will be collected to assess hematological, renal, and hepatic function and status, and to assess for pregnancy.

9.3.5 Follow up for relapses or recrudescence

There are no documented cases of relapse with supervised high dose primaquine (30 mg per day for 14 days). In our previous studies no relapses were observed during a 2-year follow up. All volunteers will be contacted at least by telephone at three months intervals after completion of directly observed primaquine therapy (two weeks) and negative blood smear.

9.3.5.1 Chloroquine resistance

Chloroquine resistant *P. vivax* malaria has been documented only rarely in Colombia (Soto 2001) and has not been observed with mixed treatment (chloroquine plus primaquine) (Soto 2001). However, in the unexpected case where the smear is noted to be positive at any time of the long TBS follow-up (days 7,14,28 after initiation of treatment), volunteer will receive alternative treatment with Fansidar^{R} (SP) as 3 tablets in a single dose (25 mg of pyrimethamine plus 500 mg of sulfadoxine per tablet). If the patient has a contraindication to Fansidar (i.e. sulfa allergy), he/she will receive amodiaquine as above and will then be subjected to additional TBS follow up to confirm cure.

9.3.5.2 Follow up for parasitemia for relapses or recrudescence:
There are no documented cases of relapse with supervised high dose primaquine (30 mg per day for 14 days) (Baird and Hoffman 2001). Volunteers in this study will be administered directly observed therapy, every day for fourteen days at MVDC. Any *P. vivax* relapses will be treated with chloroquine and a repeat course of primaquine (dosing identical to first treatment course) with follow-up identical to the first course of therapy as above. Long-term follow-up for possible relapses by *P. vivax* hypnozoites will be performed. All volunteers will be contacted at least by telephone at defined time-points after completion of directly observed primaquine therapy (2 weeks) and negative blood smear (Table 2).

9.4 Proposed phone follow-up post treatment

		Acceptable Window
Within one month	Weeks 1, 2, 3 and 4	+/- 3 days
Within two months	Weeks 6 and 8	+/- 5 days
Within three months	Weeks 10 and 12	+/- 7 days
Within six months	Weeks 16, 20, 24	+/- 10 days
Within a year	Weeks 30, 38, 46, and 52	+/- 10 days
Within 1 and 1/2 years	Weeks 52, 60, 68 and 76	+/- 14 days

In addition, all volunteers who have been challenged will be requested to contact the study physician, or to ask that their medical care providers contact the study physicians for advice concerning the diagnosis and treatment of malaria infection, in case a fever develops. If fever, shaking chills, or other symptoms possibly related to malaria develop at any time within 1 year and a half after challenge, the physician seeing the volunteer must be informed that they have been exposed to an experimental malaria infection and the volunteer MUST have a TBS and PCR checked

10 Clinical Evaluations
10.1 Clinical evaluations for Step 1
10.1.1 Recruitment
As described before, recruitment will be made from patients with febrile illnesses from one of the outpatient facilities designed in this study for malarial diagnosis and recruitment. The patient will have a complete medical history made and also a physical examination carried-out by a certified physician. All personal antecedents will be asked, taking in special consideration,

previous infectious illnesses, sexual transmission diseases, illicit-drug use, previous blood transfusions, and medication currently taken, (including prescribed and over-the-counter drugs) taken in the last week before the initial symptoms. A format specially designed to register medical history and with specific questions to assess these items will be applied. After this procedure, TBS will be taken and if identified with high levels of *P. vivax* gametocytemia are found, he (she) will ask to participate in this research.

10.1.2 Follow-up after malarial treatment

After malarial treatment, participants will be asked to return 2 weeks later (Day 10) to have a new physical examination and a TBS done to assure malarial cure. If malarial disease is present at this contact, new treatment will be given. At this time, new schedules for physical examination and TBS will be done base on individual cases.

If the volunteer did not attend at the 10 day to the medical appointment, participants will be called to their contact number provided in the informed consent, also active search will be done in their residence location. Some of them can live in dangerous armed conflict areas, therefore they will be not located in their place if the investigator security will be compromised.

10.2 Clinical evaluations for Step 2

10.2.1 Recruitment

Once volunteers sign informed consent, a medical history will be taken. History about current acute and chronic diseases will be asked. History about insect allergies, food allergies, Asthma, infectious diseases, surgery, medications used (prescribed and over-the-counter), previous inpatient treatments, illicit-drug use, alcohol usage, pregnancy, will be explicitly asked. All this will be done by a certified physician. Formats specifically constructed that includes all this items will be systematically applied. Also a complete physical exam (including body weight, blood pressure, cardiac frequency, and axillary's temperature) will be performed and documented. Also an EKG will be performed to exclude any rhythm abnormalities or previous ischemic events.

10.2.2 Pre-immunization

A new abbreviated medical questionnaire will be applied; specially searching for any clinical signs that will alter the immunization schedule or exclude the participant to it. Also a physical examination will be performed immediately before the initiation of the immunization phase. This will include ENT system, cardiovascular, pulmonary, neurological, gastrointestinal, urogenital, and dermatology systems. Body weight and vital signs (Blood pressure, cardiac frequency, and axillary's temperature) will be measured again at this time and during the rest of the study.

10.2.3 Immunization

Before each immunization, the same medical abbreviated questionnaire will be applied and a physical exam will be performed. The skin used for mosquito biting will be thoroughly examined. After each immunization on days 1 and 2, the same medical abbreviated questionnaire used before, will be applied. No standard physical exam will be performed, but vital signs and mosquito biting site over the skin will be examined. If necessary a 1% Hydrocortisone ointment and oral anti-histamine will be given (Loratadine 10 mgr/dose). All this follow-up visits will be recorded and Adverse Event report will be filled if necessary. During the immunization scheme,

and before the schedule visit, the participant will be contacted every 5 days by phone to apply a signs and symptoms questionnaire. The participant will be free to contact research personnel to report any sign and/or symptoms. Research personal will be assigned to answer for these calls and take action if necessary.

10.2.4 Malaria treatment after immunization

Before giving malarial treatment after immunization to Fy(+) E group, the same abbreviated medical questionnaire will be applied and a physical examination will be performed. Fifteen days (15) after treatment a new appointment will be made for physical examination and TBS.

10.3 Clinical evaluations for Step 3

10.3.1 Pre-Challenge

Once the window period after immunization has passed and before challenge, a new abbreviated medical questionnaire and physical examination will be performed. This will include ENT system, cardiovascular, pulmonary, neurological, gastrointestinal, urogenital, and dermatology systems. Body weight and vital signs (Blood pressure, cardiac frequency, and axillary's temperature) will be measured again at this time and during the rest of the study. The skin used for mosquito biting will be thoroughly examined.

10.3.2 Challenge

Before each immunization, the same medical abbreviated questionnaire will be applied and a physical exam will be performed. The skin used for mosquito biting will be thoroughly examined. Volunteers will be under medical observation for one hour after challenge to detect any adverse reaction to exposure to the mosquitoes. Eight hours after the challenge, all volunteers will receive a phone call to check their condition. After challenge, on days 1 and 2, the same medical abbreviated questionnaire used before, will be applied. No standard physical exam will be performed, but vital signs and mosquito biting place over the skin will be examined. If necessary a 1% Hydrocortisone ointment and oral anti-histamine will be given (Loratadine 10 mgr/dose). All this follow-up visits will be recorded and Adverse Event report will be filled if necessary.

10.3.3 Post-challenge follow up

Volunteers Fy(+) in both groups, will be evaluated by taking vital signs, mosquito biting zone inspection, thick blood smear, and real time-PCR from day 6 to day 28 after spz challenge. Then visits will continue two times per week until day 60; and weekly thereafter for 6 weeks. If after day 60 no infection can be demonstrated the proposed antimalarial scheme will be administered.

10.3.4 Follow up after antimalarial treatment for infection

Infected volunteers will be treated with chloroquine + Primaquine, and they will be evaluated by thick smear daily until three consecutive TBS are negative. Follow-up TBS will be done on day 7, day 14 and day 21 after initiation of treatment to ensure infection cure. After the last visit the volunteers will have telephone follow up through week 76.

10.3.5 Proposed phone follow-up post treatment

		Acceptable Window

Within one month	Weeks 1, 2, 3 and 4	+/-3 days
Within two months	Weeks 6 and 8	+/-5 days
Within three months	Weeks 10 and 12	+/- 7 days
Within six months	Weeks 16, 20, 24	+/-10 days
Within a year	Weeks 30, 38, 46, and 52	+/-10 days
Within 1 and 1/2 years	Weeks 52, 60, 68 and 76	+/- 14 days

10.3.6 Concomitant Medications/Treatments

All participants will eventually receive medications for malaria treatment, as described above. Those include; Chloroquine phosphate and Primaquine, administered as recommended by the guidelines of the Colombian Ministry of Health. If malarial tolerance or resistance is identified, quinine will be used.

We consider as permitted medications the use of oral contraceptives, oral antibiotics, anti-fungal agents, non-steroidal anti-inflammatory drugs, anti-histamine drugs, and corticosteroid low strength ointments for mosquito bites. For other kinds of medications that the participant is using before starting the intervention, that are not listed in the exclusion criteria, and that cannot be suspended safely, will be discussed by individually basis, with the principal investigator, to make a decision to accept them or not.

We will not allow the use of systemic corticosteroids, or other kinds of immune- or myelosuppressive agents. An exception for this will be in the case of an anaphylactic reaction, were systemic hydrocortisone is of mandatory use. If anti-malarial drugs different as those described before are needed, they should be prescribed directly by a certified physician and the reason for him to use them.

11 Laboratory Evaluations

11.1 Specimen Collection, Preparation, Handling and Shipping

11.1.1 Step 1: parasite donors

- TBS and thin smear for malarial detection, classification, and to establish gametocyte levels. (2-3 drops from finger puncture).
- Hemoglobin levels to document levels ≥ 9 g/dL (7 ml blood with EDTA)
- Infectious diseases screening, including; VIH, AgSHep B, antiCore Hep B, HCV, HTLV-1, and anti-Chagas.
- Seven mL of blood will be collected in a dry vacutainer tube (red cap) by venipuncture. Blood will be immediately fractionated into serum and cells by centrifugation at 500 g for 5 min. Vacutainers tube with cell fraction will be discarded and serum will be fractionated in identified eppendorf tubes. Information will be recorded in special forms and kept at the Center in Buenaventura following instruction of confidentiality. A copy will be send to the data management Unit located in Cali.

11.1.2 Step 2: immunized volunteers

- Laboratory screening for chronic and infectious diseases; including baseline renal and hepatic functional tests, complete CBC, and also baseline immunological evaluation (25 mL of blood)

- Blood sample for pregnancy test (1 mL of blood)
- Blood unit donation (150-200 mL of blood each time; per 2 times with about 8 to 10 months apart)
- Blood sample for immunological evaluation, CBC, renal and hepatic functional tests during immunization (20 mL of blood)
- If signs or symptoms suggestive of malarial diagnosis, repeated TBS every 12 hours or more as needed will be made to establish a diagnosis. If the diagnosis is strongly suspected and TBS has been repeatedly negative, sample for PCR will be taken.
- Tubes will be identified with the volunteer code and with the date the sample was taken. Whole blood with EDTA will be used for CBC, hemoglobin electrophoresis, and G-6-PD determination.
- Whole blood with citrate will be use for TP and TPT determination.
- Remaining blood will be immediately fractionated into serum and cells by Fycol-Percoll gradient separation and centrifugation at 500 g for 5 min. Mononuclear cells will be separated for DNA extraction to determine Duffy antigen, and remaining cells will be cryopreserved to do cellular immunological functional tests. Plasma will be used for the other proposed tests and also to humoral immunological tests (total antibodies titers, isotype titers, IFAT).
- Blood unit uses CPD as anticoagulant; the PBMC will be separated in different vials and cryopreserved using 10% DMSO and RPMI standard culture media.

11.1.3 Step 3

- Blood sample for immunological evaluation, CBC, renal and hepatic functional tests will be performed about 1 week before challenge (20 mL of blood).
- Sampling for parasite detection in blood by PCR will be started at day 5 after challenge and will be performed daily until a positive TBS (2-3 drops from finger puncture).
- Sampling for TBS for parasite detection in blood will be started at day 6 after challenge and will be performed daily. (2-3 drops from finger puncture).

12 Assessment of scientific Objectives

12.1 Specification of the appropriate outcome measures

12.1.1 Primary outcome measurement

The primary outcome of this study includes measurement of two different aspects of the intervention; those are protective efficacy, and safety.

12.1.1.1 Protective efficacy

The percentage of infected individuals will be compared among the experimental and control groups after challenge (step 3). This comparison will be used as the main measure of protective efficacy. An infected individual will be defined as an individual that presents at least one positive TBS any time after challenge and before day 60, were treatment will be given on a routine basis. As an ancillary way to measure protection we will use time from challenge to the appearance of a positive TBS; the prepatent period. This will be measured in days.

12.1.1.2 Safety

Solicited and unsolicited local and systemically adverse events will be recorded and it severity will be graded. Common terminology criteria for adverse events, version 3.0, from NIH, published August 2006, will be used for reporting them. (http://ctep.cancer.gov).

12.1.2 Secondary outcome measures

12.1.2.1 Study of humoral immune response

- Antibody responses to whole sporozoite forms by IFAT.
- Antibody responses to sporozoite antigens (e.g. PvCS, PvTRAP and PvMSP-1/200L).
- IgG isotype response profile by ELISA.
- Evaluation of the hepatocyte-sporozoite invasion and blocking ability of antibodies (In vitro test).

12.1.2.2 Evaluation on cellular immune response

- Changes in lymphocyte subpopulations and surface markers (CD3+CD4+, CD3+CD8+, CD3+CD56+, CD3-CD56+, CD3+CD4+CD25+FOXP3+, CD19/20+).
- Plasmatic levels of Th1, Th2 and Th17 cytokines.
- CD8+ T cell response after “ex-vivo” stimulation.

12.1.2.3 Novel antigen discovery

Western blotting will be used to determine novel antigens recognized by subjects’ plasma.

12.2 Methods and timing for assessing, recording and analyzing outcome measures

12.2.1 Protective efficacy measurement

As described before, a positive TBS will be used to define an infected individual. At day 6 after challenge (step 3) TBS will be routinely performed on a daily basis. Due to previous *P. vivax* challenge experiences in humans done by our group, we expect to find the first TBS to be positive around day 9 - 16 after exposure; so starting screening at day 6 appears to us as appropriate and safety. All these TBS results are going to be registered. The number of infected individuals divided by the total of participants challenged, in each group, will give us the percentage of infected individuals by group. To reject the null hypothesis of equal or more percentage of infected individuals in the experimental group compared with the control group, a one tail exact Fisher test will be performed.

Also as described before, time to the first positive TBS will be used as an ancillary way to measure protective efficacy. Time will be recorded in days. Comparison of median time to first positive TBS, and to reject the null hypothesis of equal or less median time to positive TBS, Kruskal-Wallis non-parametric one tailed test will be performed. Also the Nelson-Aalen cumulative hazard function will be estimated for each group. To test the null hypothesis the log-rank test will be performed.

12.2.2 Safety evaluation

Every time the participant assist to a mosquito bite session for immunization, and the in the follow-up, a form for solicited adverse events will be fill. Also the participants will be asked for unsolicited adverse events. Complete medical examination, CBC counts, renal and hepatic functional tests will be included in the safety parameters. Specifically laboratory results that will

be included in the safety monitoring are: WBC counts, neutrophil counts, lymphoid counts, eosinophil counts, hemoglobin levels, reticulocyte percentage, platelets counts, AST, ALT, Alkaline phosphatase, lactic deshydrogenase, total bilirubin, conjugated bilirubin, un-conjugate bilirubin, blood glucose, albumin, INR (International Normalized Ratio of prothrombine time), partial thromboplastin time (PTT), creatinine, Blood Nitrogen Urea (BUN), urine WBC, urine blood, urine proteins, urine nitrites.

12.3 Modification and discontinuation of study intervention product for a participant

We will consider modifying the immunization schedule if the participant presents a concomitant viral mild illness. We will allow postponing the immunization schedule, if there is another non-medical reason that does not precludes the continuing of the participant in the study. We allow postponing an immunization for a maximum time of 8 weeks between doses.
A study subject will be discontinued from further immunization schedule if:

- Develops malaria during immunization.
- Develops a severe allergic reaction to mosquito bites so as to put in danger his life.
- Develops a clinical condition that precludes his continuation; although the clinical condition is not related to the study protocol; including but not restricted to myocardial ischemia, viral hepatitis, an autoimmune disease, asthma, tuberculosis, HIV infection, cancer, among others.
- Any clinical condition that could preclude volunteer participation will be evaluated by a physician and decided in an individual basis.
- Other medical condition or situation occurs such that continued participation in the study would not be in the best interest of the subject.
- Refuses to comply with medical and study guidelines.
- Development of any exclusion criteria may be cause for discontinuation.
- Becomes pregnant.
- Develops a severe adverse event.

If at discretion of the DSMB (Data safety monitor board review), or the IP, intervention should be discontinued.
It is important to note that subject will continue to be followed with subject's permission if the immunization schedule is discontinued. Modification of the study intervention scheme will be discussed with the involved subject.

13 Assessment of safety

13.1 Adverse events (AE)

We will consider an AE any untoward medical occurrence in a participant included in this research. The event does not necessarily have a causal relationship with the planned intervention. Malaria infection due to irradiated sporozoites (step 2) will be considered as an AE and severity graded accordingly; malaria infection after challenge (step 3) will <u>not</u> be considered an AE.

Common terminology criteria for adverse events v3.0 (CTCAE) will be used to classify the event in a scale of 1 to 5. If the event is not registered in this document, it will be grade as followed:

1=Mild AE (awareness of a symptom but the symptom is easily tolerated)
2=Moderate AE (discomfort enough to cause interference with usual activity)

3=Severe AE (incapacitating; unable to perform usual activities; requires absenteeism or bed rest)
4=Life-threatening or disabling AE
5=Death related to AE

13.2 Serious adverse event (SAE)

We will consider as serious adverse event the following experiences:

- Death.
- Life-threatening event (subject at immediate risk of death).
- Requires in-patient hospitalization.
- Results in a persistent or significant disability or incapacity.
- Important medical events that may not result in death, be life-threatening, or require hospitalization may be considered a serious adverse event when, based upon appropriate medical judgment, they may jeopardize the subject and may require medical or surgical intervention to prevent one of the outcomes listed in this definitions. (Generally a grade 3 abnormality will be considered as a SAE).

14 Reporting procedures

14.1 Serious adverse event detection and reporting

For those events meeting the previously described definition of Serious Adverse Events (SAE), the completion of a SAE report form is required. All SAEs will be recorded on the appropriate SAE case report form, followed through resolution by a study physician, and reviewed by the principal investigator. All SAEs will be followed until satisfactory resolution or until the principal investigator or co-investigator deems the event to be chronic or the patient to be stable. Principal investigator or co-investigator will notify the monitor of the study, NIH safety officer (as sponsor), and FVL IRB/Ethical Committee within 24 hours if the SAE is death, and within 72 hours of becoming aware of the event. Notification will be made in the SAE case report form, and also a detailed description of the case.

14.2 Reporting of subject death

The death of any subject during the intervention or follow-up regardless of the cause must be reported to the NIH (sponsor) safety officer, and the IRB/Ethical Committee within 24 working hours of first becoming aware of the death. If an autopsy is performed, the report must be provided to the NIH (sponsor) and to the IRB/Ethical Committee.

14.3 Procedures to be followed in the event of abnormal findings

If during the study an abnormal laboratory test value or an abnormal clinical finding is detected, an adverse event report will be filled up by the study physician and its severity will be determined. If the event is considered as mild, treatment if needed will be administered by the study physician and the report of case report form will be filled up. The participant will continue in the study unless he denotes the contrary. If this mild abnormality is only an abnormal test

value, with other clinical manifestations, the laboratory will be repeated to confirm abnormality, before taking it as an AE. This is especially relevant for coagulation tests and for urinary tests.

If the event is considered as moderate, initial treatment will be given by the study physician, but if necessary, consultation to a specialist will be done; also the principal investigator will be informed within first 72 hours of becoming aware of it, and AE form will be filled up. Decision to exclude the participant of the research protocol will be made taken by the principal investigator.

If the event is detected by an unsolicited symptom by phone communication, the participant will have a compulsory medical examination within 12 hours of the communication. Laboratory tests and appropriate treatment will be done if considered necessary by the study physician.

The principal investigator will inform the medical monitor of any significant AE, and depending upon the nature of the AE, the medical monitor will make a decision regarding the study, or will convene the IRBs to make a decision regarding the study. Volunteers will be informed of unexpected AE that appear during the course of the investigation and which could potentially affect their safety or assessment of risk. The study will comply with national and international requirements for studies involving human subjects. The principal investigators will coordinate the interaction between the research group and the medical monitor, and other external monitoring groups. The study will not begin until approval by the IRBs.

14.4 Type and duration of the follow-up of subjects after AE

Local AE are expected in almost all participants in every one of the immunization sessions. We expect participants to have local pain, swelling, erythema, and itching; in the skin exposed to mosquito biting. Less probably we expect to see local skin or deeper infections. Severity is expected to vary among participants. Within systemically AEs we expect are, nauseas, fatigue, transient fever, and headache. Less probably we expect exanthema, severe allergic reaction (anaphylaxis) or renal function tests alterations. Malaria as AE in the immunization phase can be observed, and appropriate treatment will be given.

After one hour in place observation after mosquito biting, further follows up will be done personally on the days 1 and 2. Following evaluations will be done each 15 days before the next immunization. These follow up will include a new clinic evaluation and the AE report. Phone contact is planned to be done about 8 hours after each immunization session. Follow-up after challenge (step 3) will be similarly done, but with the difference that at day 6 the participant have to personally assist to TBS, PCR tests. All the required information will be available to the volunteers to contact the members of the research group at any time (including mobile phone number); also they will be encouraged to make questions in case of doubt.

All AEs, including onset and end dates, severity and relationship to study drug must be recorded in the subject's study records and report forms. The AEs summary CRF will include the total duration of the AEs, but not the dates. The severity of the AE and relationship to study drug will be assessed by the principal investigator or designee according to specific guidelines (CTCAE v3.0). Any action or outcome will also be recorded for each AE.

14.5 Reporting of pregnancies

Although not considered a SAE (unless an event occurs with a serious outcome), pregnancy information on clinical study subjects will be collected, and reported to the NIH safety officer. If as subject should become pregnant during the course of the study, the principal investigator or designee must contact the sponsor safety officer within 5 working days of the PI or designee first becoming aware of the pregnancy. The NIH safety officer will provide instructions via fax, phone or email, on how to collect pregnancy information.

15 Clinical monitoring structure

15.1 Site monitoring plan

An independent clinical monitor will be auditing compliance of the trial and specifically procedures considered critical to the development of the study and safety of the participants. Critical procedures will be considered; compliance in the selection criteria, informed consent administration, allocation procedures, safety visits and tests, control and reporting AEs, preservation of confidentiality of the information, data handling and preservation.

16 Statistical considerations

16.1 Overview and study objectives

To demonstrate that it is possible to safely protect human volunteers immunized with P *vivax* irradiated sporozoites from P *vivax* challenge with live sporozoites. This will show that the development of a pre-erythrocytic stage vaccine is feasible and also will give important insights about the development of an effective immune response in human beings.

Data entry will be manually verified by the Clinical Monitor and corrected if it is necessary by an authorized investigator. Data sets will then be printed and a subset will be compared to source documents in order to determine error rate. All inconsistencies of information will be reported to the Clinical Monitor for review and correction.

16.2 Study population

Malaria naïve healthy volunteers between the ages of 18 and 45 years old will be selected as participants. These volunteers will be initially divided in a group Fy(+) and Fy(-). Fy(-) individuals will be sequentially allocated to intervention. Fy(+) participants will be randomly allocated to an Experimental group (E) and a Control group (C). E group will be exposed to the immunization schedule with irr-spz, C group with mock intervention, Fy(-) group with non-irradiated sporozoites. After this a challenge with non-irradiated sporozoites will be made, and the development of malarial infection will be recorded.

16.3 Study outcome measures

Protective efficacy and safety are included as primary outcomes. Humoral and cellular immune responses evaluation are considered as secondary outcomes.

16.4 Sample size considerations

Step 3 and groups Fy(+) of this project are the ones that drives the estimation of the sample size, for the entire research process. We expect that about 80% or more of the experimental Fy(+)

group challenged with malaria vivax infected mosquitoes, do not present blood parasitemia and clinical symptoms of malarial disease. On the other hand, we expect that about 95% or more of the Fy(+) control group will present blood parasitemia and clinical symptoms of malarial disease, after challenge with infected mosquitoes. So our null and alternative hypotheses are as follows:

$$H_O : P(I|E) \geq P(I|C)$$
$$H_A : P(I|E) < P(I|C)$$

If:
I = Volunteers positive for blood parasitemia
E= Volunteers in the Experimental group
C= Volunteers in the Control group

Fixing an **a** error = 0.05, and a **b** error = 0.10, with an allocation of 2:1 for E vs C, we use the following formulae to estimate the sample size:

$$n = \frac{\left(Z_{1-\alpha}\sqrt{(c+1)^{*}p(1-p)} + Z_{1-\beta}\sqrt{cp_1(1-p_1)+p_2(1-p_2)}\right)^2}{c(p_1-p_2)^2}$$

Where:
p=(p1 +p2)/2: Average be among experimental and control group proportion.
m: required sample size for the C group
n: required sample size for the E group
c = n/m: Ratio between the number of controls for each sample.
$Z_{1-\alpha}$ = Confidence level.
$Z_{1-\beta}$ = Power of the study.
P_1 = assumed event proportion for the outcome of interest in the E group
P_2 = assumed event proportion for the outcome of interest in the C group

Confidence level and power of study is present on Table 1 and Table 2:

Table 1. Confidence level distribution.

**α**	**Z_1**
0.200	0.84
0.150	1.04
0.100	1.28
0.050	1.65
0.025	1.96
0.010	2.33

Table 2. Power of the study distribution.

β	Power of the study	Z_1
0.01	0.99	2.33
0.05	0.95	1.65
0.10	0.90	1.28
0.15	0.85	1.04
0.20	0.80	0.84
0.25	0.75	0.67
0.30	0.70	0.52
0.35	0.65	0.39
0.40	0.60	0.25
0.45	0.55	0.13
0.50	0.50	0.00

The sample size is estimate for different p_1 values, with a constant value for p_2 with assignations for *n: m* 1:1 and *2:1* respectively (Table 3 and Table 4) Where $p2$=0.95, α=0.05, β=0.20.

Table 3. Sample size according to the protection study for a 1: 1 control and experimental group

c	Protection	p_1	P_2	*p*	*n*	m	Total sample
1	0.40	0.57	0.95	0.76	15	15	29
1	0.45	0.52	0.95	0.74	12	12	24
1	0.50	0.48	0.95	0.71	10	10	20
1	0.55	0.43	0.95	0.69	9	9	17
1	0.60	0.38	0.95	0.67	7	7	15
1	0.70	0.29	0.95	0.62	5	5	11
1	0.80	0.19	0.95	0.57	4	4	8

Table 4. Sample size according to the protection study for a 2: 1 control and experimental group

c	Protection	p_1	P_2	*p*	*n*	m	Total sample
2	0.40	0.57	0.95	0.76	20	10	30
2	0.45	0.52	0.95	0.74	17	8	25
2	0.50	0.48	0.95	0.71	14	7	21
2	0.55	0.43	0.95	0.69	12	6	18
2	0.60	0.38	0.95	0.67	10	5	15
2	0.70	0.29	0.95	0.62	8	4	12
2	0.80	0.19	0.95	0.57	6	3	9

This gives us around 7 volunteers in the C group and 14 volunteers in the E group, for a total sample size of 21 participants for step 3. Because of the duration and difficulties of the immunization scheme, we expect to have about 20% of drop-off the study. Making that the sample size for step 2 should be around 28; 14 participants in the E group and 7 participants for C group.

Taking into account that we expect to have ≤50% blood parasitemia in the E group, and considering exposing the minimum participants to an ineffective intervention; we considered that when half the sample size have been recruited an interim analysis will be done. If in this analysis blood parasitemia in the E group is present in >50% of the group, the study will stop recruitment for intervention step 3, but follow-up of enrolled participants will continue as proposed.

16.5 Participant enrollment and follow-up

The expected total number of participants to be recruited will be of 28; 21 Fy(+) and 7 Fy(-). Volunteers will be recruited from Cali, a non-endemic malaria city, through diverse activities such as conferences, open meetings and posters. The material for this phase will be previously approved by the Institutional Review Board (IRB) – CECIV. The time estimated for this activity is approximately six months. Adherence of the participants to the study schedule will be enhanced using recalling phone calls, and assuring their transportation to the study site. Clinical and laboratory follow-up will be carried out as described before.

16.6 Variables

16.6.1 Dependent variables

Malarial infection after challenge: It will be considered as the presence of a positive TBS after challenge. It is a dichotomical variable. From this, protective efficacy will be obtained.

Time to first positive TBS after challenge: It is the time from challenge to first positive TBS. It will be managed in the analyses as a numerical continuous variable, in days.

Adverse events: Number, type, duration and causation of the AE will be collected.

Immune response measures: Immune response measures are described before. They will be managed initially as continuous variables, and transformed to normality if necessary. Afterwards some will be managed as categorical variables as stated above, and as necessary.

16.7 Independent variables

Intervention group: Three groups are considered to be intervening. Experimental, Control and Fy(-) groups. Comparison will be done between E and C groups for protective efficacy. For immune response tests E vs Fy(-), E vs Fy(-), and Fy(-) vs C, pairs will be compared.

16.8 Covariables

Age:Time from born date to the recruitment visit will be taken as age; it will be managed as a numerical continuous variable, and also as a categorical variable with 3 groups; 18-26, 27-35, 36-45

Gender: Gender will be classified as male or female, based on phenotype in the first clinical examination.

Afrocolombian: Afrocolombian as dychotomic variable (yes/no) will be based on phenotype in the first clinical examination.

Weight: Body weight will be taken at recruitment visit. Will be use as a numerical continuous variable in the analyses. Weight will be also use as a categorical variable using tertiles for classification.

16.9 Analysis plan

Clinical data will be entered into a database program name REDCap. Data entry will be manually verified by the Clinical Monitor and corrected if it is necessary by an authorized investigator. Data sets will then be printed and a subset (15%) will be compared to source documents in order to determine error rate. All inconsistencies of information will be reported to the Clinical Monitor for review and correction.

A flow diagram will be constructed to describe number of screened individuals, number of eligible participants, and number of subjects that finally accepted participation. Number allocated to each group, number of subjects that were lost during the intervention (and causes of desertion), and finally number of participants that continued to challenge and follow-up.
Univariated analysis will be done to obtain descriptive statistics; such as central tendency summary estimates, dispersion estimates, absolute and relative frequencies.

The number of participants with positive TBS will be described by group and percentages and 95%CI intervals will be estimated. Percentage of malarial infection in group E and group C will be compared, and null hypothesis of no difference or greater infection in group E, will be rejected using an exact Fisher's one tail test. P-value less than 0.05, will be taken as enough evidence to reject the null hypothesis proposed.

The period of time between challenge and the first positive TBS will be used as an ancillary way to measure protective efficacy. Time will be recorded in days. Comparison of median time to first positive TBS among groups will be made to reject the null hypothesis of equal or less median time to positive TBS in the E group. Kruskal-Wallis non-parametric one tailed test will be performed. Also the Nelson-Aalen cumulative hazard function will be estimated for each group. To test the null hypothesis the log-rank test will be performed.

The number of subjects reporting any AEs, the occurrence of specifics AEs, and discontinuation due to AEs will be tabulated. Frequency of AEs will be cross-tabulated by group for each immunization session. Although no inferential analysis of safety data is planned, if a substantial different in specific AEs should be seen, comparison of tabular data will be done using two-tailed Fisher exact test. A table with cumulative AEs for all immunization session is planned; again if a substantial different in an specific AEs should be seen, percentage comparison will be

made, taking into account that these are not independent data and so a cluster appropriate statistical analysis will be performed.

Whisker and box graphs will be performed to evaluate immune response tests distributions, among groups, and also by basal demographic variables; for each time immune response tests were measured.

Geometric means of total, specific antibodies and isotype titers will be compared among the groups at each point of time, in a transversal fashion; if normality presumption is maintained ANOVA will be used, in not a non-parametric test such as Wilcoxon paired t test or Kruskal-Wallis will be performed. Also geometric means of total, specific antibodies and isotype titers will be compared within each group at different points of time, to evaluate its trends (longitudinal analysis). As we expect a non-linear relationship of antibody responses and other immunological tests with time, graphical display will be used for initial exploration of the data. Median splines and/or Lowess smoothing will be used for constructing these graphs. Depending on the type of the relationship found in these graphics, an appropriate statistical model will be proposed taking into account that has to be repeated measure analysis.

Mean levels of anti-sporozoite antibodies will be compared among groups at different points of time. Also the percentage of individuals with anti-sporozoite antibodies >1:20 in each group at different points of time will be compared

Comparison of percentage of inhibition will be done among the groups at each point of time, in a transversal fashion. Rejection of one-tail null hypothesis of equal inhibition of sporozoite invasion to hepatoma cells among groups will be performed, using Fisher's exact tests.

17 Potential pitfalls

The maximal immunization doses for humans (~10 inoculations) may not be protective in any (or in most = 8/14) of the volunteers. In this case, we will treat the volunteers and repeat the challenge two to three weeks after treatment. Challenge will presumably boost immune responses.

Irradiation doses may be either too low and end up in infection, or induce no immunity because of complete spz inactivation (death). In the first case (if >4 immunized volunteers develop parasitemia), the irradiation dose will be increased; in the second case, the dose will be decreased. The second case will be presumed to apply if no immune responses are detected after several (> 4) immunizations.

Patients may withdraw spontaneously, or due to secondary reaction to mosquito bites. In this case, new volunteers will be engaged from the group of alternate volunteers.

Every attempt will be made to coordinate challenge and sample collection so that data will be comparable among volunteers. We cannot guarantee each volunteer's availability on challenge day. In the previous studies, however, volunteers were very cooperative. It is also difficult to guarantee dates for the availability of sporozoites due to the fact that mosquito infections are

sometimes suboptimal. However, in general, back-up mosquito batches will be prepared, resulting in only minimal delays in challenge. These uncertainties may lead to small differences between the challenge schedules of volunteers, but we are confident that these will not compromise the trial endpoints.

Sporozoite load are insufficient blood donor’s inclusion criteria will be improved (higher percentage of parasitemia) and short cultures to gametocyte production will be performed.

Real time PCR and may not be able to detect parasites (merozoites) released from the liver. For this we are currently analyzing DNA samples collected during a close follow up based on the pre-patent period of the recent spz challenge study. These results will provide guidance before initiation of the proposed studies.

The maximal immunization dose for Fy-/- human volunteers (1,500 infected bites/total doses) may not be protective. However, immune responses detected will be very valuable to understand the immunity development process.

18 Ethics/protection of human subjects

18.1 Declaration of Helsinki

The investigator will ensure that this study is conducted in full conformity with the current revision of the Declaration of Helsinki and guidelines; whichever afford the greater protection to subjects or with the International Conference on Harmonization (ICH)

18.2 Institutional review board

This protocol and its associated informed consent documents and recruitment materials will be presented and approved by the CECIV (IRB). This ethics committee is considered independent of the research study group, and is registered with the OHRP. Any amendments to the protocol or consent materials must also be approved before they are placed into use.

18.3 Informed Consent Process

Informed consent is a process that is initiated prior to individual’s agreeing to participate in the study and continuing throughout the individual’s study participation. Extensive discussion of risks and possible benefits of participation in this study will be provided to the subjects and their families. This documents will be presented and approve by the Institutional Review Board (IRB) in human research of Centro Internacional de Vacunas (CECIV). This committee belongs to our institution. Any modification to the protocol should be approved before their application.

Written informed consent will be obtained from the subject. The informed consent will describe the purpose of the study, the procedures to be followed, and the risks and benefits of participation. A copy of the consent form will be given to the subject. The study may be discontinued at any time by the NIAID.

The following information relates to the primary research site, which is located in the in Colombia endemic areas for malaria *P.vivax*. The Institutional Review Board (IRB) will conduct the review, approval and continuing review of the investigation, and no subject will be admitted

to the study before this IRB issues its written favorable opinion of the study. Composition, functions and operation of this commission are in consonant with national (Colombian Science Council) and international regulations on clinical research.

Individuals will be enrolled outpatient of different clinics in the endemic areas. At that time, information will be presented to enable persons to voluntarily decide whether or not to participate as a research subject. They will receive complete information on the benefits and risks of participating in the study, the confidentiality of the collected information, and a thorough explanation of the procedures that she/he will be involved in case of accepting to participate, including clinical examinations and blood sampling. The physician of the research team will also make clear to potential participants that during the study they have the right to withdraw without any justification or any penalties. Informed consent will be documented in a written form that will be read to the subject but, in any event, they will also have adequate opportunity to read the form before it is signed. The written consent form will need to be previously approved by the CECIV and signed and dated by the subject or the subject's parent/guardian at the time of consent. A copy will be given to the person signing the form.

In the case that an illiterate person will be interested in participating in the study, two witnesses who can read and write will be present during the reading aloud of the consent. It will be certified that all questions have been resolved and all procedures of the clinical trial are already understood and

18.4 Subject Confidentiality

Information obtained in the study will be managed based on principles of confidentiality. Subject confidentiality will be strictly held in trust by the investigator and authorized personnel. The study protocol, documentation, data, and all other information generated will be held in strict confidence and will be protected in the locked boxes. No information concerning the study or its data will be released to any unauthorized third party without prior written approval of the sponsor.

The study monitor or authorized representatives of sponsor may inspect all documents and records required to be maintained by the investigator including, but not limited to, medical records (office, clinic, or hospital), and pharmacy records for the patients in this study. The clinical study site will permit access to such records. Records may be examined by monitors, audits, and/or health authorities and regulators as needed. All individuals reviewing the records are bound by the rules of strict confidentiality.

Subjects will not be identified by name on any study documents. Subjects will be identified by the Participant Identification Number (PID) and Study Identification Number (SID). During data entry and validation, database files will be accessible by designated data entry personnel only, and will be password protected.

18.5 Future use of stored specimens

Blood samples will be obtained and handled by the Clinical Research Coordinators according to the MVDC Standard Operating Procedures. After adequate labeling with the study code for each

volunteer (described in section 6.1) all samples will be immediately transferred to the laboratory for storage. If residual specimens will be maintained after the study is complete, we will include provisions for consent and the options that are available for the volunteer to agree to the future use of his/her specimens. Specimens will be coded maintained in secure refrigerators at the MDVC or Immunology Institute if the CECIV will review future studies. Genetic testing will not be performed if required by the CECIV.

19 Data handling and record keeping

19.1 Data Collection

All information about the participants in the study will be collected in source documents. The information needed to perform analysis of the primary and secondary outcomes will be recorded on case report forms (CRF). All data on the CRF will be legibly recorded in black ink. Corrections will be made by striking through the incorrect entry with a single line and entering the correct information adjacent to it. Corrections will be initialed and dated by the investigator or assigned study staff. Any requested information that is not obtained as specified in the protocol will have an explanation noted on the CRF as to why the required information was not obtained.

Protocols have been set up for the collection and transfer of samples and data from the field. Standard operating procedures (SOPs) have been implemented for physicians, lab technicians, and entomologists. Clinical and laboratory forms will be completed and signed by study investigators, stored in bound folders while in use, and then secured in locked metal file cabinets. Source documents, including lab reports and patient records, should be maintained in the participant's medical chart or study file and should be available for review. As required, this original protocol, all reports, consent forms, questionnaires, and other pertinent protocol records will otherwise and thereafter be kept in the MVDC once the study has been completed. These records will be stored for 10 years.

19.2 Data entry

Data will be double entered by two separate individuals using REDCap software, and checked for entry error and range error. Access to the system will be restricted to authorized study personnel. Data analysis will be performed electronically using the statistics program STATA® (Version 8.0).

19.3 Data queries

The data management unit will do centralized editing of all data, by weekly manual generation of edit queries and corrections. In addition, regular staff meetings and telephone conference calls will be held to review progress and to discover and solve problems.

After entry, the data files will be converted to Stata 10.0 (Version 10.0) using Stat-transfer, and every week programs will be run to verify the consistency of responses within each questionnaire. All detected inconsistencies will be resolved by correction against original CRF or lab books. A 10% random sample of the blood films for measuring parasitemia and gametocytemia will be re-examined by technicians at CIC to validate diagnosis.

19.4 Timing/Reports

No interim analyses are planned as the number of participants is to low. Although monitoring of malaria associated to immunization will be done, so to decide if it is necessary to increase radiation dose. Reports of study activities and advances solicited by the sponsor will be send at due time.

19.5Study records retention

Records, including source data documents and CRFs will be stored as a minimum of 3 years after completion of the trial.

20 Protocol Deviations

A protocol deviation is any noncompliance with the clinical trial protocol, GCP, or manual of procedures requirement. The noncompliance may be either on the part of the subject, the investigator, or the study site staff. It is the responsibility of the site to use continuous vigilance to identify and report deviations within 5 working days of identification of the protocol deviation, or within 5 working days of the scheduled protocol-required activity. All deviations must be promptly reported to CECIV.

All protocol deviations should be record in the database of the study. A complete copy of the Deviation of Protocol format should be stored in the regular field and in the participant's database. The protocol deviations should be sent to the IRB according to the guidelines. The principal investigator and their crew research members are responsible to known and follow up the guidelines of the CECIV.

It is the responsibility of the site to use continuous vigilance to identify and report deviations within 5 working days of identification of the protocol deviation, or within 5 working days of the scheduled protocol-required activity. All deviations must be promptly reported to the Division of Microbiology and Infectious Diseases (DMID).

All deviations from the protocol must be addressed in study subject source documents. A completed copy of the DMID Protocol Deviation Form must be maintained in the regulatory file, as well as in the subject's source document. Protocol deviations must be sent to the local IRB/IEC per guidelines. The site primary investigator and study staff are responsible for knowing and adhering to their IRB requirements.

21 Publication policy

The results of this study are confidential and will be published only after authorization of the Principal Investigators and the sponsor institutions (NIHLB/NIH). It is anticipated that results from this protocol will be presented to scientific community via oral presentations at meetings and written publications in scientific journals.

22 References

1. Wang, R., et al., Immune responses to Plasmodium vivax pre-erythrocytic stage antigens in naturally exposed Duffy-negative humans: a potential model for identification of liver-stage antigens. Eur J Immunol, 2005. 35(6): p. 1859-1868.

2. Coban, C., et al., Manipulation of host innate immune responses by the malaria parasite. Trends Microbiol, 2007. 15(6): p. 271-278.

3. Hoffman, S.L., et al., Protection of humans against malaria by immunization with radiation attenuated Plasmodium falciparum sporozoites. J Infect Dis, 2002. 185(8): p. 1155-1164.

4. Graves, P. and H. Gelband, Vaccines for preventing malaria. Cochrane Database Syst Rev, 2003(1): p. CD000129.

5. Richie, T.L. and A. Saul, Progress and challenges for malaria vaccines. Nature, 2002. 415(6872): p. 694-701.

6. Doolan, D.L., et al., Degenerate cytotoxic T cell epitopes from P. falciparum restricted by multiple HLA-A and HLA-B supertype alleles. Immunity, 1997. 7(1): p. 97-112.

7. Mellouk, S., et al., Protection against malaria induced by irradiated sporozoites. Lancet, 1990.

335(8691): p. 721.

8. Nussenzweig, R.S., et al., Protective immunity produced by the injection of x-irradiated sporozoites of plasmodium berghei. Nature, 1967. 216(111): p. 160-162.

9. Seguin, M.C., et al., Induction of nitric oxide synthase protects against malaria in mice exposed to irradiated Plasmodium berghei infected mosquitoes: involvement of interferon gamma and CD8+ T cells. J Exp Med, 1994. 180(1): p. 353-358.

10. Weiss, W.R., Host-parasite interactions and immunity to irradiated sporozoites. Immunol Lett, 1990. 25(1-3): p. 39-42.

11. Weiss, W.R., et al., CD8+ T cells (cytotoxic/suppressors) are required for protection in mice immunized with malaria sporozoites. Proc Natl Acad Sci U S A, 1988. 85(2): p. 573-576.

12. Collins, W.E. and P.G. Contacos, Immunization of monkeys against Plasmodium cynomolgi by Xirradiated sporozoites. Nat New Biol, 1972. 236(67): p. 176-177.

13. Collins, W.E., et al., Immunization of Saimiri sciureus boliviensis with recombinant vaccines based on the circumsporozoite protein of Plasmodium vivax. Am J Trop Med Hyg, 1989. 40(5): p. 455-464.

14. Gwadz, R.W., et al., Preliminary studies on vaccination of rhesus monkeys with irradiated sporozoites of Plasmodium knowlesi and characterization of surface antigens of these parasites. Bull World Health Organ, 1979. 57 Suppl 1: p. 165-173.

15. Zavala, F., et al., Circumsporozoite proteins of malaria parasites contain a single immunodominant region with two or more identical epitopes. J Exp Med, 1983. 157(6): p. 1947-1957.

16. Egan, J.E., et al., Humoral immune responses in volunteers immunized with irradiated Plasmodium falciparum sporozoites. Am J Trop Med Hyg, 1993. 49(2): p. 166-173.

17. Clyde, D.F., Immunity to falciparum and vivax malaria induced by irradiated sporozoites: a review of the University of Maryland studies, 1971-75. Bull World Health Organ, 1990. 68 Suppl: p. 9-12.

18. Clyde, D.F., et al., Specificity of protection of man immunized against sporozoite-induced falciparum malaria. Am J Med Sci, 1973. 266(6): p. 398-403.

19. Clyde, D.F., et al., Immunization of man against sporozite-induced falciparum malaria. Am J Med Sci, 1973. 266(3): p. 169-177.

20. Clyde, D.F., Immunization of man against falciparum and vivax malaria by use of attenuated sporozoites. Am J Trop Med Hyg, 1975. 24(3): p. 397-401.

21. Rieckmann, K.H., Human immunization with attenuated sporozoites. Bull World Health Organ, 1990. 68 Suppl: p. 13-16.

22. Rieckmann, K.H., et al., Letter: Sporozoite induced immunity in man against an Ethiopian strain of Plasmodium falciparum. Trans R Soc Trop Med Hyg, 1974. 68(3): p. 258-259.

23. Rieckmann, K.H., et al., Use of attenuated sporozoites in the immunization of human volunteers against falciparum malaria. Bull World Health Organ, 1979. 57 Suppl 1: p. 261-265.

24. Herrington, D., et al., Successful immunization of humans with irradiated malaria sporozoites: humoral and cellular responses of the protected individuals. Am J Trop Med Hyg, 1991. 45(5): p. 539-547.

25. Krzych, U., et al., T lymphocytes from volunteers immunized with irradiated Plasmodium falciparum sporozoites recognize liver and blood stage malaria antigens. J Immunol, 1995. 155(8): p. 4072- 4077.

26. Glynn, J.R., Infecting dose and severity of malaria: a literature review of induced malaria. J Trop Med Hyg, 1994. 97(5): p. 300-316.

27. Glynn, J.R., et al., Infecting dose and severity of falciparum malaria. Trans R Soc Trop Med Hyg, 1995. 89(3): p. 281-283.

28. Powell, R.D. and J.V. McNamara, Infection with chloroquine-resistant Plasmodium falciparum in man: prepatent periods, incubation periods, and relationships between parasitemia and the onset of fever in nonimmune persons. Ann N Y Acad Sci, 1970. 174(2): p. 1027-1041.

29. Chulay, J.D., et al., Malaria transmitted to humans by mosquitoes infected from cultured Plasmodium falciparum. Am J Trop Med Hyg, 1986. 35(1): p. 66-68.

30. Church, L.W., et al., Clinical manifestations of Plasmodium falciparum malaria experimentally induced by mosquito challenge. J Infect Dis, 1997. 175(4): p. 915-920.

31. Bejon, P., et al., Calculation of liver-to-blood inocula, parasite growth rates, and preerythrocytic vaccine efficacy, from serial quantitative polymerase chain reaction studies of volunteers challenged with malaria sporozoites. J Infect Dis, 2005. 191(4): p. 619-626.

32. van Dijk, M.R., et al., Genetically attenuated, P36p-deficient malarial sporozoites induce protective immunity and apoptosis of infected liver cells. Proc Natl Acad Sci U S A, 2005. 102(34): p. 12194- 12199.

33. Hurtado, S., et al., Regular production of infective sporozoites of Plasmodium falciparum and P. vivax in laboratory-bred Anopheles albimanus. Ann Trop Med Parasitol, 1997. 91(1): p. 49-60.

34. Zapata, J.C., et al., Reproducible infection of intact Aotus lemurinus griseimembra monkeys by Plasmodium falciparum sporozoite inoculation. J Parasitol, 2002. 88(4): p. 723-729.

35. Herrera, S., et al., Successful Sporozoite Challenge Model in Human Volunteers with*Plasmodium vivax* Strain Derived from Human Donors. Am J Trop Med Hyg, 2009. 81 (5):740-746.

36. Herrera, S., et al., Implication of Plasmdium vivax sporozoite challenge system in vaccine development, in International Research in Infectious Diseases. 2007. p. 53.

37. Herrera, S., et al., Antigenicity and immunogenicity of multiple antigen peptides (MAP) containing P. vivax CS epitopes in Aotus monkeys. Parasite Immunol, 1997. 19(4): p. 161-170.

38. Herrera, S., et al., Use of long synthetic peptides to study the antigenicity and immunogenicity of the Plasmodium vivax circumsporozoite protein. Int J Parasitol, 2004. 34(13-14): p. 1535-1546.

39. Jordan-Villegas, A., et al., Cellular and humoral responses to Plasmodium vivax irradiated sporozoites in Aotus monkeys. (In preparation), 2007.

40. Hollingdale, M.R. and U. Krzych, Immune responses to liver-stage parasites: implications for vaccine development. Chem Immunol, 2002. 80: p. 97-124.

41. Yoshida, N., et al., Hybridoma produces protective antibodies directed against the sporozoite stage of malaria parasite. Science, 1980. 207(4426): p. 71-73.

42. Hollingdale, M.R., et al., Inhibition of entry of Plasmodium falciparum and P. vivax sporozoites into cultured cells; an in vitro assay of protective antibodies. J Immunol, 1984. 132(2): p. 909-913.

43. Millet, P., et al., A monoclonal antibody directed against the sporozoite stage of Plasmodium vivax binds to liver parenchymal cells. Immunol Lett, 1992. 33(3): p. 289-294.

44. Romero, P., et al., Cloned cytotoxic T cells recognize an epitope in the circumsporozoite protein and protect against malaria. Nature, 1989. 341(6240): p. 323-326.

45. Tsuji, M., et al., CD4+ cytolytic T cell clone confers protection against murine malaria. J Exp Med, 1990. 172(5): p. 1353-1357.

46. Tsuji, M., et al., Gamma delta T cells contribute to immunity against the liver stages of malaria in alpha beta T-cell-deficient mice. Proc Natl Acad Sci U S A, 1994. 91(1): p. 345-349.

47. Renggli, J., et al., Elimination of P. berghei liver stages is independent of Fas (CD95/Apo-I) or perforin-mediated cytotoxicity. Parasite Immunol, 1997. 19(3): p. 145-148.

48. Calvo-Calle, J.M., et al., Binding of malaria T cell epitopes to DR and DQ molecules in vitro correlates with immunogenicity in vivo: identification of a universal T cell epitope in the Plasmodium falciparum circumsporozoite protein. J Immunol, 1997. 159(3): p. 1362-1373.

49. Ferreira, A., et al., Inhibition of development of exoerythrocytic forms of malaria parasites by gamma-interferon. Science, 1986. 232(4752): p. 881-884.

50. Maheshwari, R.K., et al., Recombinant human gamma interferon inhibits simian malaria. Infect Immun, 1986. 53(3): p. 628-630.

51. Weiss, W.R., et al., A T cell clone directed at the circumsporozoite protein which protects mice against both Plasmodium yoelii and Plasmodium berghei. J Immunol, 1992. 149(6): p. 2103-2109.

52. Sedegah, M., F. Finkelman, and S.L. Hoffman, Interleukin 12 induction of interferon gammadependent protection against malaria. Proc Natl Acad Sci U S A, 1994. 91(22): p. 10700-10702.

53. Hoffman, S.L., et al., Sterile protection of monkeys against malaria after administration of interleukin- 12. Nat Med, 1997. 3(1): p. 80-83.

54. Photini, S. and E. Nardin, Sporozoite Antigens: Biology and Immunology of the Circumsporozoite Proteina dnd Thrombosponding related Anonymous. Malaria Immunology, ed. Karger. 2002: Chemical Immunology. 97-124.

55. Fidock, D.A., et al., Plasmodium falciparum liver stage antigen-1 is well conserved and contains potent B and T cell determinants. J Immunol, 1994. 153(1): p. 190-204.

56. Good, M.F., et al., Human T-cell recognition of the circumsporozoite protein of Plasmodium falciparum: immunodominant T-cell domains map to the polymorphic regions of the molecule. Proc Natl Acad Sci U S A., 1988. 85(4): p. 1199-1203.

57. Guebre-Xabier, M., R. Schwenk, and U. Krzych, Memory phenotype CD8+ T cells persist in livers of mice protected against malaria by immunization with attenuated Plasmodium berghei sporozoites. European Journal of Immunology, 1999. 29(12): p. 3978-3986.

58. López, J.A., et al., Immunogenicity of synthetic peptides corresponding to the nonrepeat regions of the P. falciparum circumsporozoite protein. Vaccines 96. Cold Spring Harbor Laboratory 1996: p. 255-260.

59. Perlaza, B.L., et al., Immunogenicity of four Plasmodium falciparum preerythrocytic antigens in Aotus lemurinus monkeys. Infect Immun, 1998. 66(7): p. 3423-3428.

60. Perlaza, B.L., et al., Immunogenicity and protective efficacy of Plasmodium falciparum liver-stage Ag-3 in Aotus lemurinus griseimembra monkeys. Eur J Immunol, 2003. 33(5): p. 1321-1327.

61. Herrera, S., et al., Efficiency of human Plasmodium falciparum malaria vaccine candidates in Aotus lemurinus monkeys. Mem Inst Oswaldo Cruz, 1992. 87 Suppl 3: p. 423-428.

62. Valderrama-Aguirre, A., et al., Antigenicity, immunogenicity, and protective efficacy of Plasmodium vivax MSP1 PV200l: a potential malaria vaccine subunit. Am J Trop Med Hyg, 2005. 73(5 Suppl): p. 16-24.

63. Arevalo-Herrera, M., et al., Immunogenicity and protective efficacy of recombinant vaccine based on the receptor-binding domain of the Plasmodium vivax Duffy binding protein in Aotus monkeys. Am J Trop Med Hyg, 2005. 73(5 Suppl): p. 25-31.

64. Arevalo-Herrera, M., et al., Induction of transmission-blocking immunity in Aotus monkeys by vaccination with a Plasmodium vivax clinical grade PVS25 recombinant protein. Am J Trop Med Hyg, 2005. 73(5 Suppl): p. 32-37.

65. Jordan-Villegas, A., et al., Aotus lemurinus griseimembra monkeys: a suitable model for Plasmodium vivax sporozoite infection. Am J Trop Med Hyg, 2005. 73(5 Suppl): p. 10-15.

66. Herrera, S., et al., Safety and elicitation of humoral and Cellular responses in Colombian Malaria-Naive Volunteers by a Plasmodium vivax Circumsporozoite Protein-Drived Synthetic Vaccine. Am. J.Trop. Med. Hyg, 2005. 73: p. 3-9.

67. Fernández, O., et al. Development of a sporozoite challenge model for Plasmodium vivax in human volunteers. in Annual meeting 54th American Society of Tropical Medicine and Hygiene. December 11-15. 2005. Washington: Am Trop J Med. Hyg. .

68. Arevalo-Herrera, M. and S. Herrera, Plasmodium vivax malaria vaccine development. Mol Immunol, 2001. 38(6): p. 443-455.

69. Castellanos, A., et al., Plasmodium vivax Trombospondin Related Adhesion Protein: Inmunogenicity and protective efficacy in rodents and Aotus monkeys. Memórias do Instituto Oswaldo Cruz 2007. 102(3): p. 411-416.

70. Rogers, W.O., K. Gowda, and S.L. Hoffman, Construction and immunogenicity of DNA vaccine plasmids encoding four Plasmodium vivax candidate vaccine antigens. Vaccine, 1999. 17(23-24): p. 3136-3144.

71. Stowers, A.W., et al., Efficacy of two alternate vaccines based on Plasmodium falciparum merozoite surface protein 1 in an Aotus challenge trial. Infect Immun, 2001. 69(3): p. 1536-46.

72. Collins, W.E., et al., Testing the efficacy of a recombinant merozoite surface protein (MSP-1(19) of Plasmodium vivax in Saimiri boliviensis monkeys. Am J Trop Med Hyg, 1999. 60(3): p. 350-6.

73. Oliveira-Ferreira, J., et al., Immunogenicity of Plasmodium vivax merozoite surface protein-9 recombinant proteins expressed in E. coli. Vaccine, 2004. 22(15-16): p. 2023-30.

74. Yazdani, S.S., et al., Evaluation of immune responses elicited in mice against a recombinant malaria vaccine based on Plasmodium vivax Duffy binding protein. Vaccine, 2004. 22(27-28): p. 3727-37.

75. Kocken, C.H., et al., High-level expression of Plasmodium vivax apical membrane antigen 1 (AMA- 1) in Pichia pastoris: strong immunogenicity in Macaca mulatta immunized with P. vivax AMA-1 and adjuvant SBAS2. Infect Immun, 1999. 67(1): p. 43-9.

76. Malkin, E.M., et al., Phase 1 vaccine trial of Pvs25H: a transmission blocking vaccine for Plasmodium vivax malaria. Vaccine, 2005. 23(24): p. 3131-8.

77. Hisaeda, H., et al., Antibodies to malaria vaccine candidates Pvs25 and Pvs28 completely block the ability of Plasmodium vivax to infect mosquitoes. Infect Immun, 2000. 68(12): p. 6618-23.

78. Arevalo-Herrera, M., et al., Plasmodium vivax: transmission-blocking immunity in a malaria-endemic area of Colombia. Am J Trop Med Hyg, 2005. 73(5 Suppl): p. 38-43.

79. Manzano, M.R., et al., Standardization of Anopheles albimanus infection with Plasmodium vivax for sporozoite challenge of naive volunteers. (In preparation), 2005.

80. Snounou, G., Detection and identification of the four malaria parasite species infecting humans by PCR amplification. Methods Mol Biol, 1996. 50: p. 263-291.

81. Baird, J.K., et al., Chloroquine for the treatment of uncomplicated malaria in Guyana. Ann Trop Med Parasitol, 2002. 96(4): p. 339-348.

82. Cavasin, C., et al., Plasmodium vivax infection among Duffy antigen-negative individuals from the Brazilian amazon region: an exception? Trans R Soc Trop Med Hyg 2007. (in press).

83. Ryan, J., et al., Evidence for transmission of Plasmodium vivax among a Duffy antigen negative population in western Kenya. Am J Trop Med & Hyg, 2006. 75: p. 581-775.

84. Arevalo-Herrera, M., et al., Mapping and comparison of the B-cell epitopes recognized on the Plasmodium vivax circumsporozoite protein by immune Colombians and immunized Aotus monkeys. Ann Trop Med Parasitol, 1998. 92(5): p. 539-551.

85. Herrera, S., et al., Human recognition of T cell epitopes on the Plasmodium vivax circumsporozoite protein. J Immunol, 1992. 148(12): p. 3986-3990.

86. Arevalo-Herrera, M., et al., Identification of HLA-A2 restricted CD8(+) T-lymphocyte responses to Plasmodium vivax circumsporozoite protein in individuals naturally exposed to malaria. Parasite Immunol, 2002. 24(3): p. 161-169.

87. Perlaza, B.L., et al., Long synthetic peptides encompassing the Plasmodium falciparum LSA3 are the target of human B and T cells and are potent inducers of B helper, T helper and cytolytic T cell responses in mice. Eur J Immunol, 2001. 31(7): p. 2200-2009.

88. Miller, L.H., et al., Erythrocyte receptors for (Plasmodium knowlesi) malaria: Duffy blood group determinants. Science, 1975. 189(4202): p. 561-3.

89. Herrera, S., et al., Antibody response to Plasmodium vivax antigens in Fy-negative individuals from the Colombian Pacific coast. Am J Trop Med Hyg, 2005. 73(5 Suppl): p. 44-49.

90. Ocana-Morgner, C., M.M. Mota, and A. Rodriguez, Malaria blood stage suppression of liver stage immunity by dendritic cells. J Exp Med, 2003. 197(2): p. 143-51.

91. Coban, C., et al., Toll-like receptor 9 mediates innate immune activation by the malaria pigment hemozoin. J Exp Med, 2005. 201(1): p. 19-25.

92. Engwerda, C.R. and M.F. Good, Interactions between malaria parasites and the host immune system. Curr Opin Immunol, 2005. 17(4): p. 381-7.

93. Scheller, L.F. and A.F. Azad, Maintenance of protective immunity against malaria by persistent hepatic parasites derived from irradiated sporozoites. Proc Natl Acad Sci U S A, 1995. 92(9): p. 4066-4068.

94. Mueller, A., et al., Genetically modified Plasmodium parasites as a protective experimental malaria vaccine. Nature, 2005. 433(7022): p. 164-167.

95. Ampudia, E., et al. Estrategia genomica para la identificacion de candidatos a vacuna in Sexto Congreso Colombiano de Alergia, Asma e Inmunologia. 2007. Medellin, Colombia.

96. Hernández, M.A., et al., Polymorphism of the Plasmodium vivax circumsporozoite gene and its implication for vaccine design. (In preparation), 2006.

97. Zuñiga, E., et al., Polymorphism of fragment 200L of gen MSP-1 of Plasmodium vivax in clinical isolated of the Colombia Pacific Coast (In preparation), 2006.

98. Mangold, K.A., et al., Real-time PCR for detection and identification of Plasmodium spp. J Clin Microbiol, 2005. 43(5): p. 2435-2440.

99. Baird, J.K., et al., Randomized, parallel placebo-controlled trial of primaquine for malaria prophylaxis in Papua, Indonesia. Clin Infect Dis, 2001. 33(12): p. 1990-1997.

100. Nussenzweig, R., J. Vanderberg, and H. Most, Protective immunity produced by the injection of xirradiated sporozoites of Plasmodium berghei. IV. Dose response, specificity and humoral immunity. Mil Med, 1969. 134(10): p. 1176-1182.

101. Chatterjee, S., et al., Immunity to Plasmodium berghei exoerythrocytic forms derived from irradiated sporozoites. Parasitol Res, 1996. 82(4): p. 297-303.

102. Malkin, E., F. Dubovsky, and M. Moree, Progress toward the development of malaria vaccines. Trends Parasitol, 2006. 22(7): p. 292-295.

103. Beier, J.C., et al., Quantitation of Plasmodium falciparum sporozoites transmitted in vitro by experimentally infected Anopheles gambiae and Anopheles stephensi. Am J Trop Med Hyg, 1991. 44(5): p. 564-570.

104. Doolan, D.L. and S.L. Hoffman, Pre-erythrocytic-stage immune effector mechanisms in Plasmodium spp. infections. Philos Trans R Soc Lond B Biol Sci, 1997. 352(1359): p. 1361-1367.

105. Doolan, D.L. and N. Martinez-Alier, Immune response to pre-erythrocytic stages of malaria parasites. Curr Mol Med, 2006. 6(2): p. 169-85.

106. Hoffman, S.L., et al., Attacking the infected hepatocyte. In Malaria Vaccine Development: A Multi- Immune Response Approach, ed. S.L. Hoffman. 1996, Washington, DC: ASM Press. 35-75.

107. Nussenzweig, R.S., et al., Specificity of protective immunity produced by x-irradiated Plasmodium berghei sporozoites. Nature, 1969. 222(192): p. 488-489.

108. Nardin, E.H., et al., Cellular and humoral immune responses to a recombinant P. falciparum CS protein in sporozoite-immunized rodents and human volunteers. Bull World Health Organ, 1990. 68 Suppl: p. 85-87.

109. Procell, P.M., W.E. Collins, and G.H. Campbell, Circumsporozoite protein of the human malaria parasite Plasmodium ovale identified with monoclonal antibodies. Infect Immun, 1988. 56(2): p. 376-379.

110. Rathore, D., et al., An immunologically cryptic epitope of Plasmodium falciparum circumsporozoite protein facilitates liver cell recognition and induces protective antibodies that block liver cell invasion. J Biol Chem, 2005. 280(21): p. 20524-20529.

111. Cox, R.J., et al., An early humoral immune response in peripheral blood following parenteral inactivated influenza vaccination. Vaccine, 1994. 12(11): p. 993-999.

112. Crotty, S., et al., Tracking human antigen-specific memory B cells: a sensitive and generalized ELISPOT system. J Immunol Methods, 2004. 286(1-2): p. 111-122.

113. Malaspina, A., et al., Compromised B cell responses to influenza vaccination in HIV-infected individuals. J Infect Dis, 2005. 191(9): p. 1442-1450.

114. Palmer, D.R. and U. Krzych, Cellular and molecular requirements for the recall of IL-4-producing memory CD4(+)CD45RO(+)CD27(-) T cells during protection induced by attenuated Plasmodium falciparum sporozoites. Eur J Immunol, 2002. 32(3): p. 652-61.

115. Mills, K.H., Regulatory T cells: friend or foe in immunity to infection? Nat Rev Immunol, 2004. 4(11): p. 841-55.

116. Di Fabio, S., et al., Quantitation of human influenza virus-specific cytotoxic T lymphocytes: correlation of cytotoxicity and increased numbers of IFN-gamma producing CD8+ T cells. Int Immunol, 1994. 6(1): p. 11-19.

117. Wang, R., et al., Induction of CD4(+) T cell-dependent CD8(+) type 1 responses in humans by a malaria DNA vaccine. Proc Natl Acad Sci U S A, 2001. 98(19): p. 10817-10822.

118. Wang, R., et al., Induction in humans of CD8+ and CD4+ T cell and antibody responses by sequential immunization with malaria DNA and recombinant protein. J Immunol, 2004. 172(9): p.5561-5569.

119. Nijman, H.W., et al., Identification of peptide sequences that potentially trigger HLA-A2.1-restricted cytotoxic T lymphocytes. Eur J Immunol, 1993. 23(6): p. 1215-1219.

120. Calvo-Calle, J.M., G.A. Oliveira, and E.H. Nardin, Human CD4+ T cells induced by synthetic peptide malaria vaccine are comparable to cells elicited by attenuated Plasmodium falciparum sporozoites. J Immunol, 2005. 175(11): p. 7575-7585.

121. Dieringer, D. and C. Schlotterer, Microsatellite analyser (MSA): a platform independent analysis tool for large microsatellite data sets. Molecular Ecology Notes, 2002. 3(1): p. 167-169.

122. Pritchard, J.K., M. Stephens, and P. Donnelly, Inference of Population Structure Using Multilocus Genotype Data. Genetics 155 p. 945-959.
