# Supplementary figures and images for: Protective Efficacy of Plasmodium vivax Radiation-Attenuated Sporozoites in Colombian Volunteers: A Randomized Controlled Trial
Source: PLoS Negl Trop Dis. 2016 Oct 19;10(10):e0005070. doi: 10.1371/journal.pntd.0005070 (PMC5070852; doi:10.1371/journal.pntd.0005070)

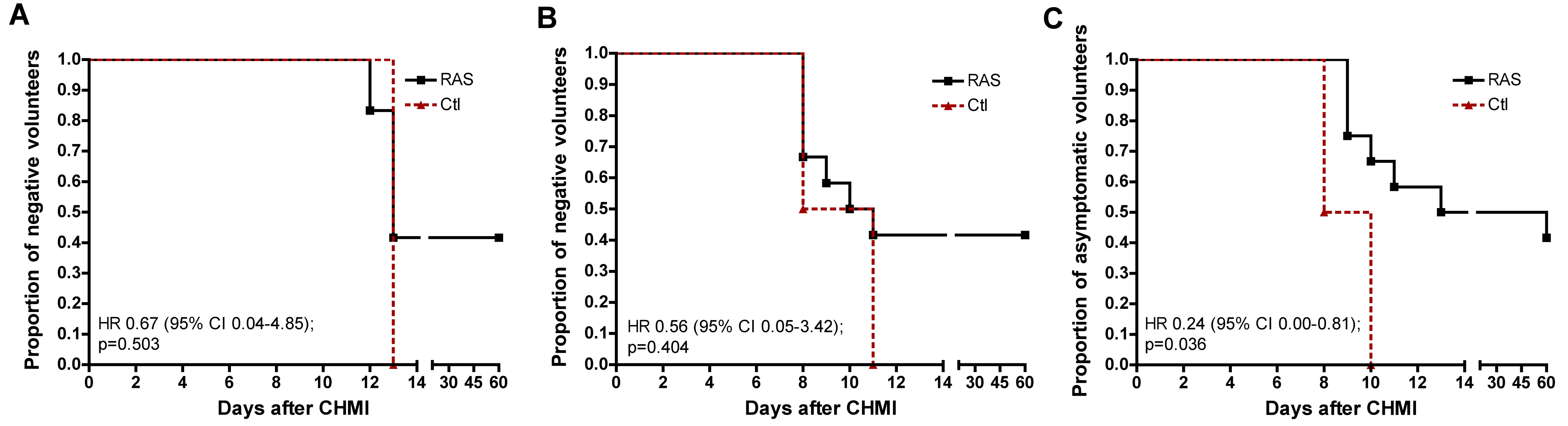

Supplement: S1 Fig — Days after CHMI to detect parasites by microscopy (A) or RT-qPCR (B) and onset of symptoms (C). (TIF) [file pntd.0005070.s003.tif]

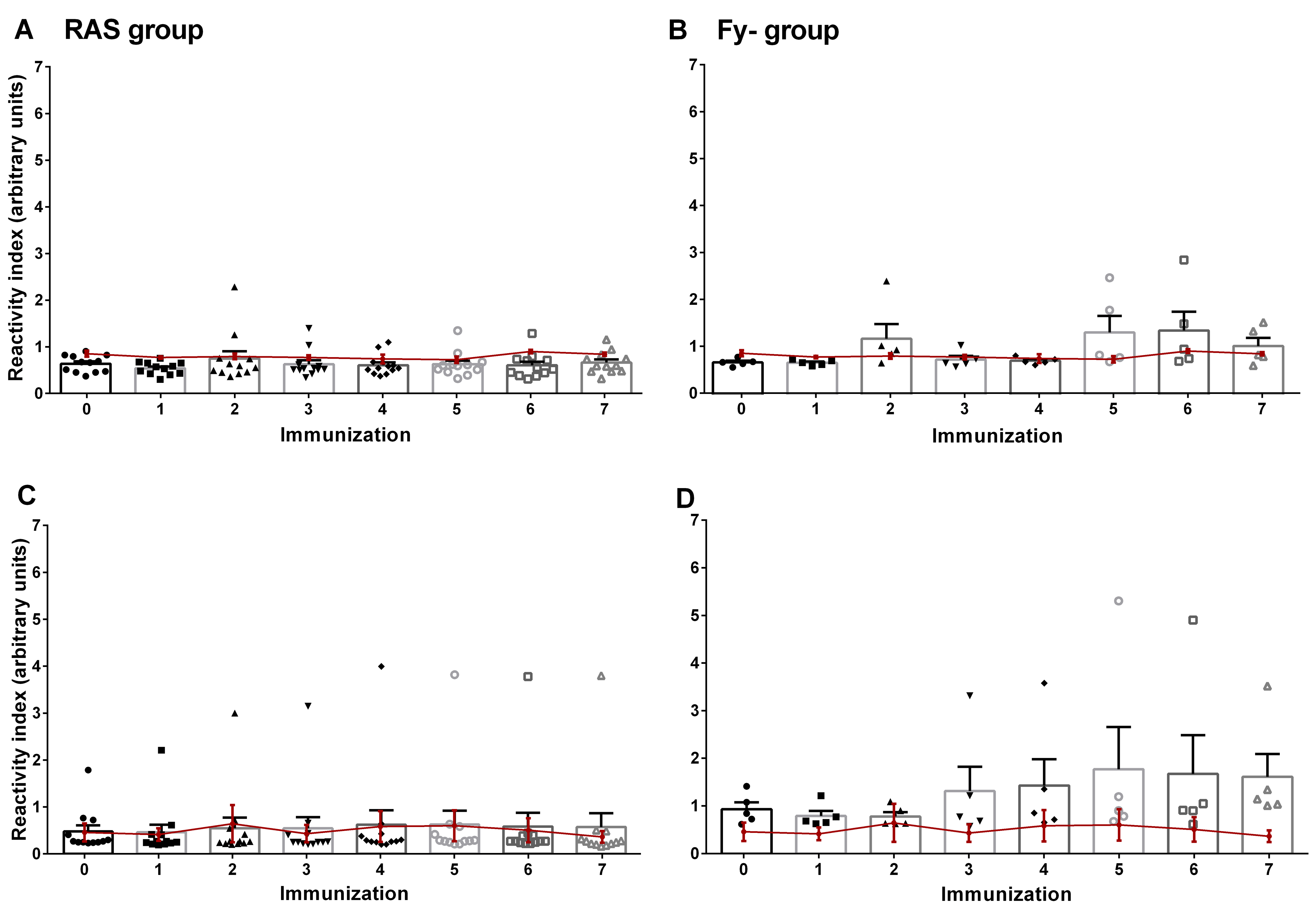

Supplement: S2 Fig — ELISA antibody response in RAS group (n = 12; A and C) and in Fy- group (n = 5; B and D) as well as in Ctl group (n = 2; red line in A to D) against PvCS-N (A-B) and PvMSP-1 (C-D) are shown. Values are expressed as reactivity index (RI) defined as sample OD at 1:200 serum dilutions divided by the cut-off value. Mean ± SEM are shown. (TIF) [file pntd.0005070.s004.tif]

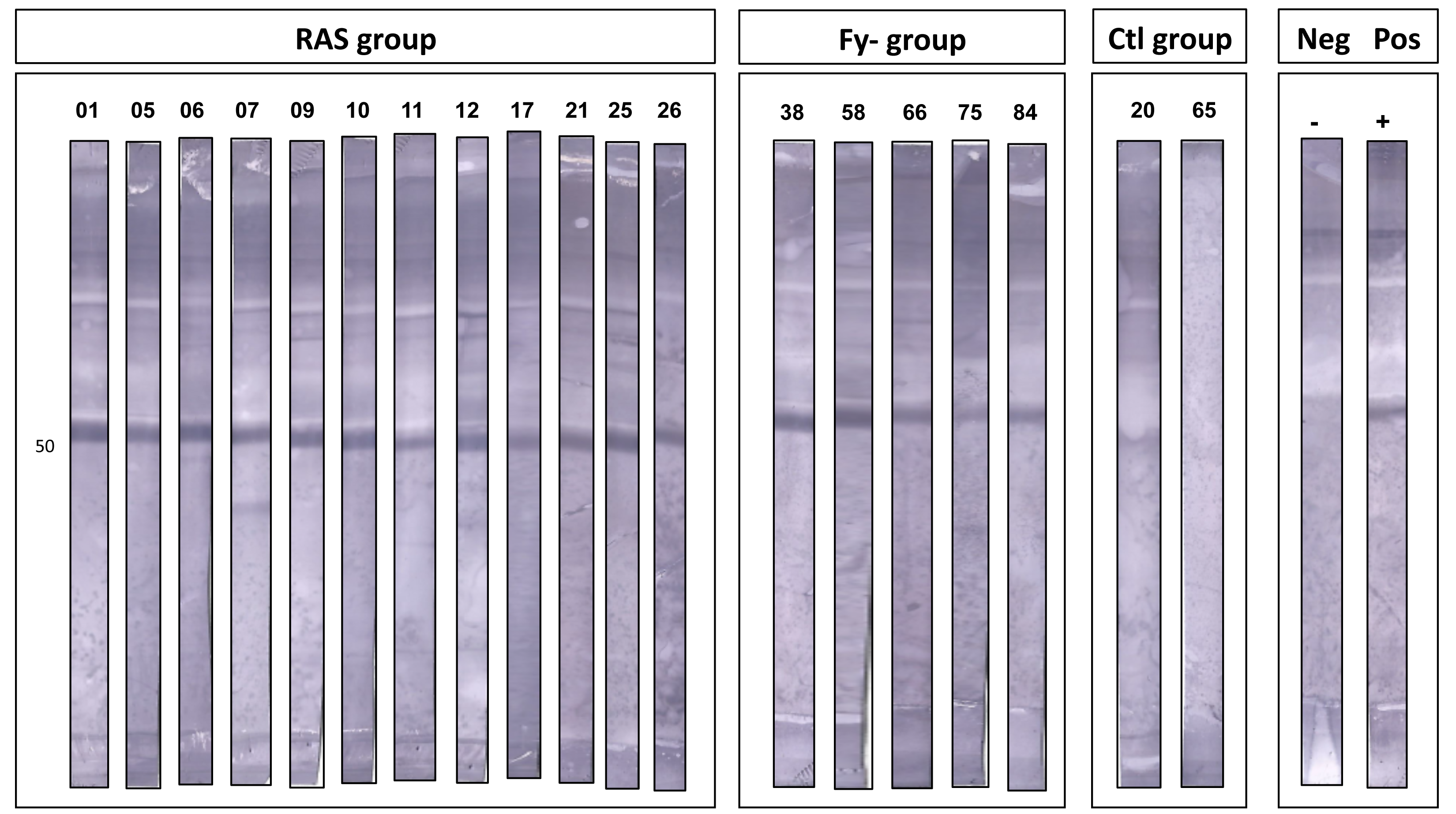

Supplement: S3 Fig — Western blot analysis of P. vivax sporozoites lysate separated on 12% SDS-PAGE under non-reducing conditions. Sera from RAS group (n = 12), Fy- group (n = 5), and Ctl group (n = 2) are shown. Negative (naïve volunteers) and positive (volunteers immunized with PvCSP) controls are also shown. Relative size standards are indicated on the left in KDa. (TIF) [file pntd.0005070.s005.tif]
