# Supplementary material for: Protective Efficacy of Plasmodium vivax Radiation-Attenuated Sporozoites in Colombian Volunteers: A Randomized Controlled Trial
Source: PLoS Negl Trop Dis. 2016 Oct 19;10(10):e0005070. doi: 10.1371/journal.pntd.0005070 (PMC5070852; doi:10.1371/journal.pntd.0005070)
Supplement: S1 Table — (DOC) [file pntd.0005070.s006.doc]

**Protective efficacy of *Plasmodium vivax* radiation-attenuated sporozoites in Colombian volunteers: a randomized controlled trial**

## S1 Table. Clinical and laboratory exclusion criteria, assessment techniques and excluded volunteers.

| **Criterion** | **Technique** | **Reference values for inclusion** | **Commercial brand** | **Patients with this criteria** | |
| --- | --- | --- | --- | --- | --- |
|  |  |  |  | **#** | **Description** |
| Pregnancy | Immunochromatography | Negative | Abon Biopharm Company | 0 | NA |
| Complete blood cell counts | Automated hematology analyzer | - | KX-21N, Sysmex | 5 | Anemia (4)  Thrombocytopenia (1) |
| Urinalysis | Urine dipstick and Microscopic examination | - | SD UroColor10 Standard Diagnostics | 6 | Trichomoniasis (1)  Hematuria (1), Glycosuria (1)  Other abnormalities (3) |
| Renal function (creatinine, BUN) | Colorimetry | Creat <1.1 mg/dL  BUN < 21 mg/dL | Biosystems | 0 | 0 |
| Hepatic function (ALT, AST, ALP, LDH) | Colorimetry | ALT ≤31 U/L; AST ≤32 U/L  ALP 98-279 U/L,  LDH 207-414 U/L | Biosystems | 0 | 0 |
| Others (CRP, casual glycemia) | Colorimetry | CRP ≤5 mg/L  Gluc <140 mg/dL | Biosystems | 1 | Diabetes (1) |
| Coagulation tests | Coagulometry | PTT 22·9-30.6s, PT 8.0-10.8s | Siemens |  |  |
| G6PDH deficiency | U.V Kinetic method | 4.6 – 13.5 U/g Hb | Trinity Biotech | 0 | NA |
| Hemoglobin variants | Liquid chromatography | 0% of S and C hemoglobin | . | 4 | Hemoglobinopathies (4) |
| Clinical assessment | - | - | - | 2 | Epididymitis (1)  Foot amputation (1) |
| Consent withdrawal before challenge/loss to follow-up | - | - | - | 14 | - |
| Other | - | - | - | 2 | Programmed surgery during the study (2) |

ALT, Alanine aminotransferase; AST, Aspartate aminotransferase; ALP, alkaline phosphatase; BUN, Blood urea nitrogen; CRP: C-reactive protein; LDH, Lactic dehydrogenase; G6PDH, Glucose-6-phosphate dehydrogenase; HBs Ag: Hepatitis B surface antigen; HTVL, human T-lymphotropic virus; PT, Prothrombin time; PTP, Partial thromboplastin time.
