# Supplementary material for: Protective Efficacy of Plasmodium vivax Radiation-Attenuated Sporozoites in Colombian Volunteers: A Randomized Controlled Trial
Source: PLoS Negl Trop Dis. 2016 Oct 19;10(10):e0005070. doi: 10.1371/journal.pntd.0005070 (PMC5070852; doi:10.1371/journal.pntd.0005070)
Supplement: S2 Table — (DOC) [file pntd.0005070.s007.doc]

**Protective efficacy of *Plasmodium vivax* radiation-attenuated sporozoites in Colombian volunteers: a randomized controlled trial**

## S2 Table. Screening of common infectious agents for recruited volunteers^a^

| **Criterion** | **Technique** | **Reference values for inclusion** | **Commercial brand** | **Individuals excluded for this criteria** |
| --- | --- | --- | --- | --- |
| Syphilis (*Treponema pallidum)*^b^ | RPR Carbon | Non reactive | Biosystems | 5 |
| HIV^c^ | Immunochromatography | Negative | Xerion/ Abon Biopharm Company | 1 |
| Chagas disease (*Trypanosoma cruzi)* | Immunochromatography | Negative | Standard Diagnostics | 0 |
| HTLV I/HTLV II | Inmunocomb | Negative | Orgenics | 0 |
| Hepatitis B virus (HBs Ag) | Immunochromatography | Negative | ad-bio | 0 |
| Hepatitis C virus | Immunochromatography | Negative | ad-bio | 0 |

^a^All tests had external monthly quality control by Progba/Cemic Bs Argentina. ^b^ Confirmatory tests were performed in external laboratories using FTA-ABS. ^c^Screening was performed using duplicate immunochromatographies of different commercial brands. Confirmatory tests are performed in external laboratory using Western Blot. HBs Ag: Hepatitis B surface antigen.
