# Supplementary material for: Protective Efficacy of Plasmodium vivax Radiation-Attenuated Sporozoites in Colombian Volunteers: A Randomized Controlled Trial
Source: PLoS Negl Trop Dis. 2016 Oct 19;10(10):e0005070. doi: 10.1371/journal.pntd.0005070 (PMC5070852; doi:10.1371/journal.pntd.0005070)
Supplement: S5 Table — (DOC) [file pntd.0005070.s010.doc]

**Protective efficacy of *Plasmodium vivax* radiation-attenuated sporozoites in Colombian volunteers: a randomized controlled trial**

## S5 Table. Indirect immunofluorescence using *Plasmodium vivax* sporozoites

| **Group** | **Volunteer code** | **5^th^ immunization**  **(titers)** | **7^th^ immunization**  **(titers)** |
| --- | --- | --- | --- |
| **RAS** | 001 | 1:40 | 1:20 |
|  | 005 | 1:160 | 1:160 |
|  | 006 | >1:160 | 1:40 |
|  | 007 | - | 1:40 |
|  | 009 | >1:160 | >1:160 |
|  | 010 | 1:160 | >1:160 |
|  | 011 | - | - |
|  | 012 | >1:160 | 1:80 |
|  | 017 | 1:40 | 1:20 |
|  | 021 | 1:160 | 1:80 |
|  | 025 | >1:160 | 1:160 |
|  | 026 | - | 1:80 |
| **Fy-** | 038 | 1:40 | 1:40 |
|  | 058 | - | >1:160 |
|  | 066 | - | 1:20 |
|  | 075 | 1:40 | 1:40 |
|  | 084 | - | - |
| **Ctl** | 020 | - | - |
|  | 065 | - | - |

Indirect immunofluorescence against whole *P. vivax* sporozoites using sera after fifth and seventh immunizations. Antibody titers were estimated using two-fold serial dilutions of the test sera starting at 1:20. Data shown are maximum dilution at which reactivity was present. In gray volunteers that were protected of infectious *P. vivax* CHMI.
