# Supplementary material for: Protective Efficacy of Plasmodium vivax Radiation-Attenuated Sporozoites in Colombian Volunteers: A Randomized Controlled Trial
Source: PLoS Negl Trop Dis. 2016 Oct 19;10(10):e0005070. doi: 10.1371/journal.pntd.0005070 (PMC5070852; doi:10.1371/journal.pntd.0005070)
Supplement: S1 Text — (DOC) [file pntd.0005070.s011.doc]

**Protective efficacy of *Plasmodium vivax* radiation-attenuated sporozoites in Colombian volunteers: a randomized controlled trial**

**S1 Appendix.** Additional methods

## Western blot analysis

Protein extracts from lysate *P. vivax* sporozoites (1 x 10^6^) were separated on 12% SDS-polyacrylamide gel under non-reducing conditions and electro-transferred to a PVDF membrane under cooling conditions overnight at 30 V. After blocking for 1 h with PBS/3% milk powder at room temperature, the membranes were incubated for 1 h first with the primary antibody solution (human sera, diluted 1:100 in PBS and 1·5% milk powder) and then with an alkaline phosphatase-conjugated goat anti-human IgG (1:1000, Sigma) as described . Bound Abs were visualized using BCIP/NBT substrate. Serum from all volunteers (RAS, Fy- and Ctl) was used at 1:100. Negative (sera pool from naïve volunteers) and positive (pool from volunteers immunized with *Pv*CSP controls were included.

## Hemoglobin analysis

Whole blood samples (100 µL) were washed with isotonic saline solution (NaCl 9g/L), the cells were lysed with three volumes of water and then 10 µL of hemolyzed samples were diluted in 1 mL mobile phase A. Analysis of hemoglobin and variants were performed with a liquid chromatographic system (Shimadzu, Kioto Japan) using a 3.5 x 0.46 cm polyCAT A^TM^ column (Poly LC, Columbia, MD). The chromatographic separation of all hemoglobin variants was achieved by gradient elution with two mobile phases: Mobile phase A (20mM Bis-tris + 2mM KCN, pH 6.96) and mobile phase B (20mM Bis-tris + 2mM KCN + 200mM NaCl, pH 6.55). Elution of hemoglobin was performed by increasing the mobile phase B from 10% to 40% and to 100% in 8 and 12 min respectively. The peak areas were measured at 415 mm. Results were reported as percentage of area of each hemoglobin (F, A, S or C) .

# References

1. Cespedes N, Arevalo-Herrera M, Felger I, et al. Antigenicity and immunogenicity of a novel chimeric peptide antigen based on the *P. vivax* circumsporozoite protein. Vaccine **2013**; 31:4923-30.

2. Herrera S, Fernandez OL, Vera O, et al. Phase I safety and immunogenicity trial of *Plasmodium vivax* CS derived long synthetic peptides adjuvanted with montanide ISA 720 or montanide ISA 51. Am J Trop Med Hyg **2011**; 84:12-20.

3. Ou CN, Rognerud CL. Rapid analysis of hemoglobin variants by cation-exchange HPLC. Clin Chem **1993**; 39:820-4.
